# LncRNA CTD-2555A7.2 promotes bone formation with LncRNA-specific cascade amplification strategy

**DOI:** 10.1038/s41598-025-05826-z

**Published:** 2025-07-01

**Authors:** Fanjin Meng, Kaiyuan Zheng, Meng Deng, Yuwen Ma, Yang Yu, Junxiong Li, Hong Chen, Shan Meng, Bin Guo, Xiaolan Guo, Li Jiao, Beilei Zeng, Chun Yang, Bing Yang, Chong Yin

**Affiliations:** 1 Department of Clinical Laboratory, Lab of Nucleic Acid Therapy, Department of Oncology, Department of Rehabilitation Medicine, Affiliated Hospital of North Sichuan Medical College, Nanchong, 637000 Sichuan China; 2 School of Laboratory Medicine, Translational Medicine Research Center, North Sichuan Medical College, Nanchong, 637000 Sichuan China; 3 School of Pharmacy, Tianjin Medical University, Tianjin, 300070 China; 4 College of Basic Medicine, Beihua University, Jilin, 132000 Jilin China; 5 National Kunming High-Level Biosafety Primate Research Center, Institute of Medical Biology, Chinese Academy of Medical Sciences and Peking Union Medical College, Kunming, Yunnan, 650000 China; 6 Department of Cell Biology, College of Basic Medical Sciences, Tianjin Medical University, 22 # Qixiangtai Road, Heping District, Tianjin, 300070 China; 7 Department of Public Health, International School, Krirk University, 3 # Soi Ramintra 1 Anusawari Subdistrict, Bang Khen District, Bangkok, 10220 Thailand; 8 Department of Oncology, Affiliated Hospital of North Sichuan Medical College, 1 # Maoyuan South Road, Nanchong City, 637000 Sichuan Province China; 9 College of Basic Medicine, Beihua University, 3999 # Binjiang East Road, Jilin City, 132000 Jilin Province China; 10 Department of Clinical Laboratory, Affiliated Hospital of North Sichuan Medical College, 1 # Maoyuan South Road, Nanchong City, 637000 Sichuan Province China

**Keywords:** Osteoporosis, LncRNA CTD-2555A7.2, Bone formation, Osteogenic differentiation, MiR-381-3p, Cell biology, Physiology

## Abstract

Osteoporosis poses a significant threat to human health. Long non-coding RNAs (LncRNAs) have been deemed as crucial regulators in the pathogenesis of osteoporosis. However, the accuracy and efficiency of LncRNA-mediated regulation of bone formation require further improvement. Our previous study identified a repeat sequence in the human-derived LncRNA CTD-2555A7.2, suggesting its potential role in osteoporosis regulation. To investigate this hypothesis, we conducted systematic functional analyses of CTD-2555A7.2 in osteogenesis and explored its mechanisms and potential therapeutic applications. Through over-expression, siRNA silencing and repeat sequence over-expression in vitro and in vivo, our research demonstrate that CTD-2555A7.2 enhances bone formation by sequestering multiple miR-381-3p molecules through its repeat sequence. Through Western blot, siRNA silencing and luciferase reporter assay, we illuminated miR-381-3p suppresses osteogenic differentiation by concurrently targeting four essential genes of the Wnt signaling pathway: *Apc*, *Lef1*, *wnt5a*, and *Lrp6*. Notably, the mRNA of CTD-2555A7.2 repeat sequence exhibited pronounced therapeutic efficacy in ovariectomy osteoporosis models. Taken together, we identified a dual-amplification osteogenic axis (CTD-2555A7.2-miR-381-Wnt) that demonstrates significant regulatory effects on osteoporosis. This study has established an important theoretical framework for understanding osteogenic LncRNA mechanisms and provides novel insights for developing targeted therapeutics against osteoporosis.

**Supplementary Information:**

The online version contains supplementary material available at 10.1038/s41598-025-05826-z.

## Introduction

As a chronic systemic skeletal disease, osteoporosis serious threat to middle-aged and elderly people through increased bone fragility and subsequent fractures^1^^,^^2^ At least 9 million Americans, 70% of them women, have clinically manifested osteoporosis, according to the most conservative estimates^3^. Osteoporosis is currently one of the leading causes of disability, reduced quality of life, and premature death in older people.

Osteoporosis involves an imbalance between bone formation and resorption^4^^5^ Overactivation of osteoclasts and/or undermining of osteoblast function leads to osteoporosis^6^. Medications used to treat osteoporosis include calcium, vitamin D, raloxifene, denosumab, teriparatide, and abaloparatide. Although effective, drug treatment can lead to side effects or drug resistance. An understanding of the molecular mechanism is essential for the development of new therapeutic approaches for the treatment of osteoporosis.

LncRNAs are a class of non-coding RNA longer than 200 nucleotides that are involved in physiological and pathological cascades^7^. They play essential roles in regulating gene expression at various levels, like epigenetic, transcriptional, and post-transcriptional levels^8^'^9^ Clinical studies reveal altered LncRNAs expression in osteoporotic patients, correlating with disease severity^10^. Circulating LncRNAs, encapsulated in exosomes, may serve as biomarkers or mediate intercellular communication, affecting distant skeletal sites^11^'^12^ These findings position LncRNAs as potential therapeutic targets, with interventions aiming to restore their expression levels or block pathogenic interactions. LncRNAs have been proposed as important regulators and potential biomarkers for osteoporosis, such as Neat1 and MALAT1^13–16^. However, most of the current research on osteogenic LncRNAs is still limited to the regulation of a single miRNA or a single protein. This severely restricts the regulatory efficacy of LncRNAs on bone formation.

Notably, we previously reported Lnc-DIF with a special sequence that can simultaneously sequester multiple miR-489-3p. Through the sequence, Lnc-DIF precisely targets miRNA and effectively regulates bone formation^17^. Therefore, if we can utilize similar repeat sequences to construct nucleic acid drugs, might simultaneously sequester multiple osteogenic miRNAs, achieving efficient and high-precisive therapeutic effects.

In this study, we found a human-derived LncRNA CTD-2555A7.2. Its gene is located on the long arm of chromosome 21 (q22.11), and the transcript length is 2154 nucleotides. CTD-2555A7.2 contains a similar repeat sequence that promotes bone formation by sequestering multiple miR-381-3p. miR-381-3p targets the Wnt signaling pathway, which is crucial for bone remodeling. This study uncovered a novel dual amplification regulatory effect of LncRNAs on bone formation and provided insights into the development of nucleic acid drugs for osteoporosis.

## Materials and methods

### Cell culture and mice model

Murine preosteoblast MC3T3-E1 cell line were purchased from Runde Biotechnology Co., Ltd. (Xi’an, China). AC16 (human cardiomyocytes, CL-0790), LX-2 (human hepatasteric cells, CL-0560), WI-38 (human embryonic lung cells, CL-0243), HEK-293 (human embryonic kidney cells, CL-0001), MO3.13 (human oligodendrocytes, CL-0772), HKb20 (human renal epithelial cells, CL-0750), and hMSC (human mesenchymal stem cell line, CP-H166) were purchased from Pricella Biotechnology Co., Ltd. (Wuhan, China). Cells were cultured in Dulbecco’s Modified Eagle Medium, High Glucose (DMEM, KeyGEN BioTECH, Nanjing, China, KGM12800-500) containing 10% Fetal Bovine Serum (FBS, OPCEL, Hohhot, China, BS-1101, 20% for MO3.13), 1% L-glutamine (Sigma, G8540, St Louis, MO), 1% penicillin (Amresco, 0242, Solon, OH) and streptomycin (Amresco, 0382). Cell cultures were maintained at 37 °C, 5% CO_2_.

Aging, hind limb unloaded (HLU) and ovariectomized (OVX) mice were adopted to establish the osteoporosis models. All mice were purchased from the Huafukang Bioscience Co., Ltd. (Beijing, China) and were treated as previously described^17^^,^^18^ Euthanasia was performed with CO_2_. We confirm that all procedures involving animals and their care were according to the ARRIVE guidelines, and approved by the Ethics Committee of North Sichuan Medical College (protocol code 2023024 and date 2023Y 06 M 07D of approval). We confirm that all methods were performed in accordance with the relevant guidelines and regulations. all efforts were made to reduce the number of the mice used and their suffering.

## Human bone specimen collection

We selected bone tissue samples from 24 elderly male patients and 33 elderly female patients, all with osteoporosis. Human femur samples were collected from Xi’an Honghui Hospital as previously described^19^. This study was approved by Biomedical Research Ethics Committee of Hong Hui hospital (3 March 2017) and Institution Review Board of the Northwestern Polytechnical University (12 September 2016). All participants were provided with informed written consent before their participation in this study. We confirm that all procedures involving human samles were in accordance with the Declaration of Helsinki.

## Isolation of bone marrow mesenchymal stem cells (BMSCs) and osteogenic staining

Mice derived bone marrow mesenchymal stem cells were isolated as previously described^19^. Alkaline phosphatase (ALP) staining and Alizarin red staining were performed for determine osteogenic differentiation of cells as previously described^20^.

ALP staining was performed using a staining kit (BCIP/NBT ALP colour development kit, Beyotime, China), cells were rinsed in PBS, fixed in neutral buffered formalin (10%) for 15 min, rinsed three times in PBS, and BCIP/NBT substrate added to wells. Finally, cells were washed in ddH2O after a blue/purple colour appeared. Plates containing cells were then scanned by a Scanner (9000 F Mark II, Canon, Japan) and recorded. Alizarin red staining was performed by 0.5% Alizarin red S (pH = 4.2, A5533, Sigma, St. Louis, MO, USA). Cells were cultured in osteogenic differentiation medium (culture medium with 50 µg·mL^−1^ Ascorbic acid (A7631, Sigma) and 5 mmol·L^−1^ β-glycerophosphate (G9422, Sigma)) for 21 days, after washed by PBS, mineralized nodules were stained with alizarin red for 30 min and them washed by tape water. Plates were then scanned and recorded.

## Real time PCR and Western blot

RT-PCR and Western blot was used to assess mRNA and protein expression levels of selected genes as previously described^20^. For RT-PCR, total RNA (E.Z.N.A.^®^ total RNA kit I, Omega Bio-TEK, R6834-02, GA, USA) was isolated, and then reverse transcribed to cDNA (HiScript^®^ II 1 st Strand cDNA synthesis kit, Vazyme, R211-02, Nanjing, China). Quantitative reverse transcriptase-PCR (q-PCR) was performed using the ChamQ Universal SYBR qPCR master mix (Vazyme, Q711-02-AA) and a LightCycler 480 II instrument (Roche, Basel, Switzerland). mRNA and miRNA expression levels were normalised to Gapdh and U6, respectively. Primers came from Tsingke, Inc. (Table 1, Beijing, China).

**Table 1:** Primers sequences for qRT-PCR.

Target gene	Sequences (5’→3’)
Human *Alp*-Forward	GGCCATTGGCACCTGCCTTA
Human *Alp*-Reverse	ACCCATCCCATCTCCCAGGAA
Human *Ocn*-Forward	GGTGCAGCCTTTGTGTCCAAGC
Human *Ocn*-Reverse	GTCAGCCAACTCGTCACAGTCC
Human *Sp7*-Forward	CCTCTGCGGGACTCAACAAC
Human *Sp7*-Reverse	AGGGTGGGTAGTCATTTGCAT
Human *Gapdh*-Forward	CATGGAGAAGGCTGGGGCTC
Human *Gapdh*-Reverse	CACTGACACGTTGGCAGTGG
Mouse *Alp*-Forward	GTTGCCAAGCTGGGAAGAACAC
Mouse *Alp*-Reverse	CCCACCCCGCTATTCCAAAC
Mouse *Runx2*-Forward	CGCCCCTCCCTGAACTCT
Mouse *Runx2*-Reverse	TGCCTGCCTGGGATCTGTA
Mouse *Gapdh*-Forward	TGCACCACCAACTGCTTAG
Mouse *Gapdh*-Reverse	GGATGCAGGGATGATGTTC
CTD-2555A7.2-Forward	TGCCTTCACCTCTGCTCTTC
CTD-2555A7.2-Reverse	AGGGTGGGTTGAGGTAGAGG
Human *Tcf7*-Forward	GCGGACATCAGCCAGAAG
Human *Tcf7*-Reverse	TCACAGTATGGGGGAGCTGT
Human *Smad4*-Forward	GCCGCATGAAGTGGATAAGG
Human *Smad4*-Reverse	TCTGGTCCGCGTGTTCTTT
hsa-miR-381-3p-RT	GTCGTATCCAGTGCAGGGTCCGAGGTATTCGCACTGGATACGACACAGAG
hsa-miR-381-3p-Forward	CGCGTATACAAGGGCAAGCT
hsa-miR-381-3p-Reverse	CCAGTGCAGGGTCCGAGGT
hsa-miR-15b-5p-RT	GTCGTATCCAGTGCAGGGTCCGAGGTATTCGCACTGGATACGACAGCCAT
hsa-miR-15b-5p-Forward	TAGCAGCACAATAGTGT
hsa-miR-15b-5p-Reverse	CCAGTGCAGGGTCCGAGGT
hsa-miR-21-5p-RT	GTCGTATCCAGTGCAGGGTCCGAGGTATTCGCACTGGATACGACTCAGAC
hsa-miR-21-5p-Forward	TAGCTTATCAGACTGATG
hsa-miR-21-5p-Reverse	CCAGTGCAGGGTCCGAGGT
hsa-miR-146a-5p-RT	GTCGTATCCAGTGCAGGGTCCGAGGTATTCGCACTGGATACGACACCAGT
hsa-miR-146a-5p-Forward	TGAGAACTGAATTCCAT
hsa-miR-146a-5p-Reverse	CCAGTGCAGGGTCCGAGGT
Mouse *Hes1*-Forward	CCAGCCAGTGTCAACACGA
Mouse *Hes1*-Reverse	AATGCCGGGAGCTATCTTTCT
Mouse *Tcf7*-Forward	CAGAATCCACAGATACAGCA
Mouse *Tcf7*-Reverse	CAGCCTTTGAAATCTTCATC
Mouse *Smad4*-Forward	TGCCTGGTCTGTGTGTTTGG
Mouse *Smad4*-Reverse	GCCACGGAGTTGTCATAGGA
Human *Apc*-Forward	TGACCTCCACTCGCCTGTCTTG
Human *Apc*-Reverse	GCCTTCTCGTTAGCCCTGAATGTC
Human *Lef1*-Forward	CTTTATCCAGGCTGGTCTGC
Human *Lef1*-Reverse	TCGTTTTCCACCATGTTTCA
Human *Lrp6*-Forward	GCTTCTGCGTGCTGCTGAGAG
Human *Lrp6*-Reverse	CCTCCAAGCCTCCAACTACAATCG
Human *wnt5a*-Forward	CAGGCGGTGGCAAGCAGAAC
Human *wnt5a*-Reverse	AGTGTGGGCAGGCAGTGGTC
LA16c-306A4.2-Forward	CAGGTGCTCAAGGTCTTCCT
LA16c-306A4.2-Reverse	TGGCTGAGACTGCTGTGATT
HP09025-Forward	GCTCCATGGAGACCTCAAGA
HP09025-Reverse	CAGGCTGTGTGGTTTGCTTT
TP73-AS1-Forward	GGGAAATACCCGACCTCAAC
TP73-AS1-Reverse	TCCAGGTCCCAAGTTCTTCC
AP001476.3–201-Forward	GCTGGGACTTCTGGACTGTG
AP001476.3–201-Reverse	CCAGGGTCTGGTGTCTGTTC
RP11-405M12.2-Forward	CACAGCCACCTCTTCCTCTC
RP11-405M12.2-Reverse	TGCTGAGGAAGGTGAGGAAG

For western blotting, cells were lysed (P0013, Beyotime, Shanghai, China), washed in pre-cooled PBS, and protein concentrations determined using bicinchoninic acid assays (23225, Thermo Fisher Scientific). Samples were electrophoresed, proteins transferred to nitrocellulose membranes (66485, Pall, Port Washington, NY, USA), membranes immersed in skimmed milk (5%), and incubated overnight at 4 °C with primary antibodies: ALP (Rabbit pAb, 1:2000; HUABIO, ET1601-21, Zhejiang, China), RUNX2 (Rabbit pAb, 1:2000; HUABIO, ET1612-47), APC (Rabbit pAb, 1:1000; HUABIO, ER1802-39), LEF1 (Rabbit pAb, 1:2000; HUABIO, HA500273), LRP6 (Mouse pAb, 1:500; HUABIO, RT1369), wnt5a (Mouse pAb, 1:1000; HUABIO, ET1706-33), GAPDH (Rabbit pAb, 1:2000; HUABIO, ET1601-4). After further washing the next day, an HRP-conjugated secondary anti-IgG (1:2000; CWBIO, CW0102, CW0103, Beijing, China) was added. Protein bands were visualised by enhanced chemiluminescence (BL520B, Biosharp, China) and exposed on an imager (Fusion FX, Vilber Lourmat, Colmar, France).

## Transfection of CTD-2555A7.2 plasmid, SiRNA and the miR-381-3p mimic and inhibitor

Full length of CTD-2555A7.2-001 sequence (Transcript: ENST00000537498.1), its non-binding region, binding region and mutant binding region were synthesized by TsingKe Int (Beijing, China) and inserted into pCDNA3.1 (+) plasmid (miaolingbio, P0157, Wuhan, China), respectively. miR-381-3p mimic (Table 2, B02001, Genepharm Int, Shanghai, China) or inhibitor (Table 2, B03001, Genepharm Int), and siRNAs targeting *wnt5a*, *Lrp6*, *Apc*,* Lef1* were synthesized by Genepharm Int (Table 2). miR-381-3p sgRNA inserted PX330 plasmid (miaolingbio, P0123, Wuhan, China) were uesd for construct miR-381-3p knock out osteogenic cell line. All transfections were performed as previously described^21^.

**Table 2:** siRNA, inhibitor and mimic.

Target gene	Sequences (5’→3’)
si-CTD-2555A7.2	GGAUAGAGAGGGAAACUGAGG
si-wnt5a	CGGATAACCTTGTAACATATT
si-LRP6	CCAAACTACAAGCCCTGCACTT
si-APC	GAATAAACATCTCCGTGAATT
si-LEF1	GUGAAGAGCAGGCUAAAUATT
mmu-miR-381-3p mimics sense	UAUACAAGGGCAAGCUCUCUGU
mmu-miR-381-3p mimics antisense	AGAGAGCUUGCCCUUGUAUAUU
mmu-miR-381-3p inhibitor sense	ACAGAGAGCUUGCCCUUGUAUA

In vitro transfections for plasmids were performed using Engreen Entranster™ H4000 Reagent (4000−6, Engreen Biosystem Co, Ltd., Beijing, China) in accordance with the provided protocol. siRNAs transfected in vitro using lipofectamine 2000 (11668030, Invitrogen, USA) according to the manufacturer’s instructions. In vivo transfections were performed using 40 µL periosteal injections of nucleic acids mixed with Entranster™ in vivo transfection reagent (18668−11−2, Engreen Biosystem Co, Ltd) into medullary cavities of mouse femurs or subcutaneously over the calvarial surface.

### Bone histomorphometric analyses

To measure mineral appositional rate (MAR) and bone formation rate (BFR/BS), double calcein labeling was performed as previously described^21^. Micro CT were performed to investigate the microstructure of mice calvaria^22^. For immunohistochemical staining analysis of OCN expression in calvarial osteogenic cells, antibody against OCN (Rabbit pAb, 1:200, Santa Cruz Biotech, sc-365797, Dallas, TX) were used^21^.

## Luciferase reporter assay

To detect interaction between CTD-2555A7.2, miR-381-3p and target genes, pMIR-Report Luciferase plasmid (miaolingbio, P0471,Wuhan, China) inserted with CTD-2555A7.2 full length, CTD-2555A7.2-miR-381-3p binding (or non-binding) sequence, and miR-381-3p- *wnt5a*/*Lrp6*/*Apc*/*Lef1* binding sequences were constructed, respectively. For analyze activities of osteogenic transcription factors, PNL1.1 plasmid (N1351, Promega, Fitchburg, WI) were inserted with optimized motif sequence of each transcription factors^23^. Transfections were performed using Engreen Entranster™ H4000 Reagent (4000−6, Engreen Biosystem Co, Ltd., Beijing, China) or Entranster™ in vivo transfection reagent (18668−11−2, Engreen Biosystem Co, Ltd) for in vivo. Luciferase reporter assay in vitro (with hMSC) and in vivo (with isolated BMSC) were performed as previously (E1910 and N1120, Promega, Fitchburg, WI, USA)^17^.

## Therapeutic CTD-2555A7.2 binding region and recombinant miR-381-3p inhibitor in OVX mice

To investigate the combinatorial therapeutic effect of CTD-2555A7.2 binding region and recombinant miR-381-3p inhibitor on osteoporosis, recombinant miR-381-3p inhibitor (nCAR/anti381) was manufactured by RQCON Biological Technology Co., Ltd. (Xi’an, China), mRNA sequence of CTD-2555A7.2 binding region were synthesized (Genepharm)^24–27^. OVX mice were injected into medullary cavity of femur at a dosage of 40 µL (including 1.2 µg RNA) at 8 and 15 days after OVX^20,22^. All mice received the same treatment as previously^17,22^.

### Establishment of calvarial defection mice model

Calvarial defection mice model were adopted to measure the in situ bone formation effect of hMSCs. Twenty 2-month-old male nude mice were purchased from Huafukang Bioscience Co., Ltd. (Beijing, China). After anesthetized, a 0.8 cm window was created on the mice calvaria, and the periosteum was exposed. The periosteum was scraped gently, and a 1.2 mm hole was created at the parietal bone using a dental drill. Matrigel (356234, BD, NJ) and hydroxyapatite (20 nm in diameter, DULY, Nanjing, China) were mixed on ice to form a hydrogel for embeding transfected hMSCs. Approximately 15 µl hydrogel was implanted onto the bone defect region of mice and keep at room temperature until coagulate. The wound was sutured after iodophor disinfection. 3 mice/group were sacrificed 1 week after the implantation for HE staining, 3 mice/group were sacrificed at week 2 for immunohistochemical staining of OCN, 4 mice/group were sacrificed at week 3 for microCT and MAR/BFR.

### Statistical analysis

All experiments were independently repeated at least three times with each done in triplicate. The statistical analyses of the data were performed with GraphPad Prism version 9.4.1 software (GraphPad Software Inc, La Jolla, CA), and ordinary one-way ANOVA was used for variance analysis with 3 or more groups. Significance between two groups was determined using Student’s t-test. The data are presented as mean ± standard deviation (SD). *P* values < 0.05 were considered statistically significant for all comparisons.

## Results

### CTD-2555A7.2 promoted osteogenic differentiation through Wnt signaling pathway

**Fig. 1:**
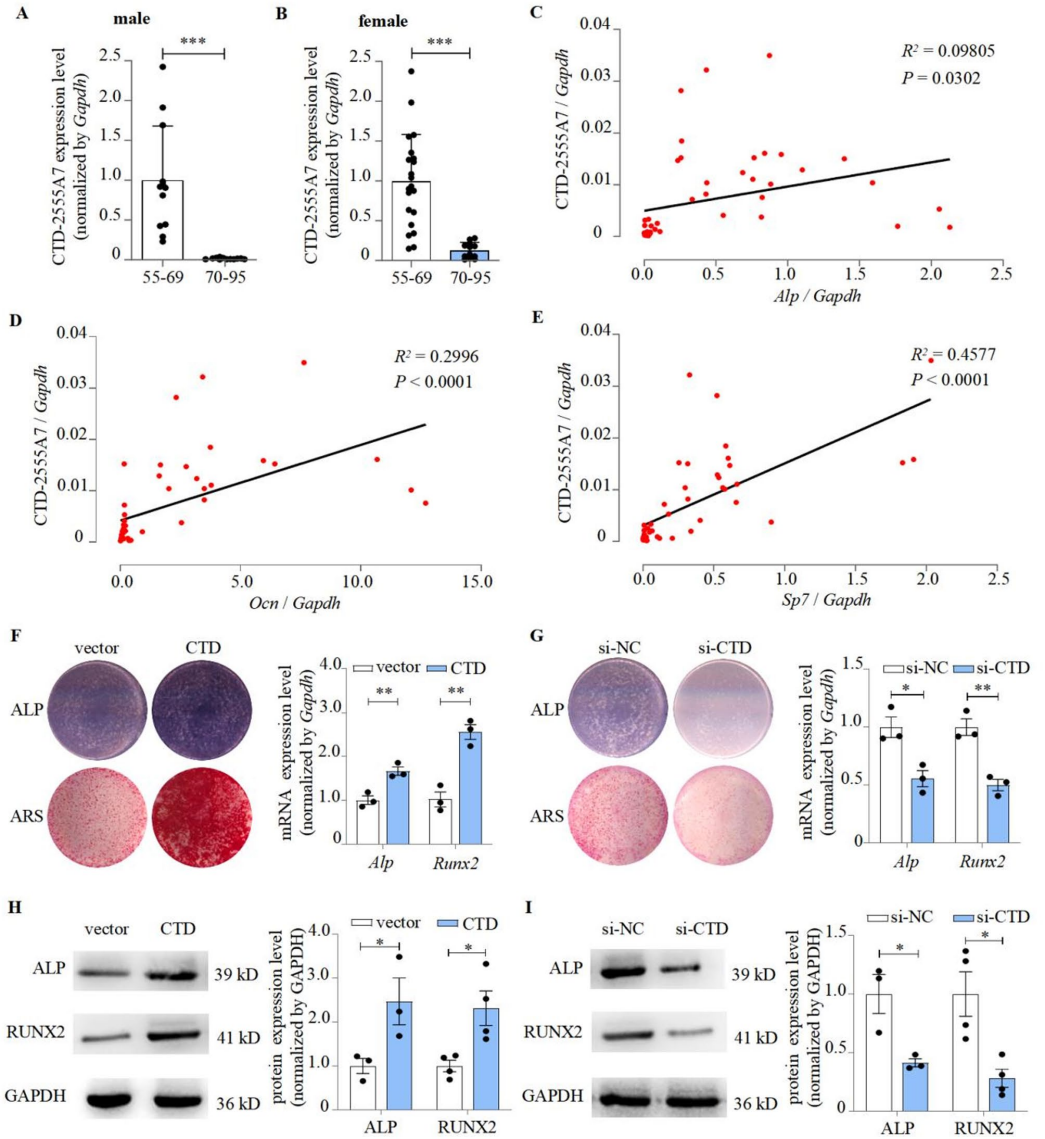
CTD-2555A7.2 promoted osteogenic differentiation. **A,B** Expression levels of CTD-2555A7.2 in 55-69 and 70-95 years old male (left) and female (right) patients with osteoporosis, as detected by RT-PCR (mean ± S.D., n > 12). 55-69: 55-69 years old osteoporosis patients. 70-95: 70-95 years old osteoporosis patients.**C-E** Correlation analysis between CTD-2555A7.2 level and *Alp*, *Ocn* or *Sp7* mRNA levels in bone tissues from osteoporosis patients, as detected by RT-PCR.**F** ALP and Alizarin Red staining of hMSCs treated with CTD-2555A7.2 over-expression plasmid (compared with pCDNA3.1 plasmid), as detected by ALP staining and Alizarin Red staining (left), and *Alp*/*Runx2* expression levels of CTD-2555A7.2 over-expression plasmid treated hMSCs, as detected by RT-PCR (right, mean ± S.D., n = 3). ALP: results of ALP staining. ARS: results of Alizarin Red staining. vector: pCDNA3.1 plasmid. CTD: CTD-2555A7.2 over-expression plasmid.**G** ALP and Alizarin Red staining (left), and *Alp*/*Runx2* expression levels (right) of hMSCs treated with CTD-2555A7.2 siRNA (compared with negative control siRNA, mean ± S.D., n = 3). si-NC: negative control siRNA. si-CTD: CTD-2555A7.2 siRNA.**H** ALP and RUNX2 protein levels of hMSCs treated with CTD-2555A7.2 over-expression plasmid, as detected by western blot. Representative western blots visualized by enhanced chemiluminescence method (left) and the quantification results with GAPDH as internal control (right, mean ± S.D., n> 3). I. ALP and RUNX2 protein levels of hMSCs treated with CTD-2555A7.2 siRNA (right, mean ± S.D., n > 3).*P < 0.05, **P < 0.01, ***P < 0.001

Our prior analysis collected bone tissue samples from aging osteoporosis patients across different age groups and identified several LncRNAs. Subsequent detection of their expression levels revealed that CTD-2555A7.2 exhibited the most significant expression decreased with age (Fig. 1A-B, Figure S1A). We detected CTD-2555A7.2 levels across multiple cell types representing distinct human tissues, including AC16 (human cardiomyocytes; heart), LX-2 (human hepatasteric cells; liver), WI-38 (human embryonic lung cells; lung), HEK-293 (human embryonic kidney cells; kidney), MO3.13 (human oligodendrocytes; nervus system), HKb20 (human renal epithelial cells; epithelium) and hMSC (human bone marrow mesenchymal stem cells; osteogenic tissue). Among these, CTD-2555A7.2 exhibited the highest expression in hMSCs, suggesting a high correlation between CTD-2555A7.2 and bone tissue (Figure S1B). CTD-2555A7.2 expression was positively correlated with osteogenic marker genes, including *Alp*, *Ocn*, and *Sp7* (Fig. 1C-E). The expression of the osteogenic marker genes ALP and RUNX2 was significantly increased by CTD-2555A7.2 overexpression (Fig. 1F, H). The blue-violet complexes (ALP) and the mineralized nodules stained with alizarin red (ARS) were also enhanced (Fig. 1F). This phenomenon could be reversed by the knockdown of CTD-2555A7.2 using siRNA (Fig. 1G, I). These findings indicated that CTD-2555A7.2 was able to promote osteogenic differentiation.

We further investigated the underlying mechanism using CTD-2555A7.2 overexpressed hMSCs. Luciferase reporter assay showed significantly increased activity of TCF7 and SMAD4 (Figure S3A-H)^28–31^. However, only Tcf7 expression was positively correlated with CTD-2555A7.2 (Figure S3I-J), suggesting that Wnt signaling is involved.

### CTD-2555A7.2 promoted bone formation through binding with multiple miR-381-3p

**Fig. 2:**
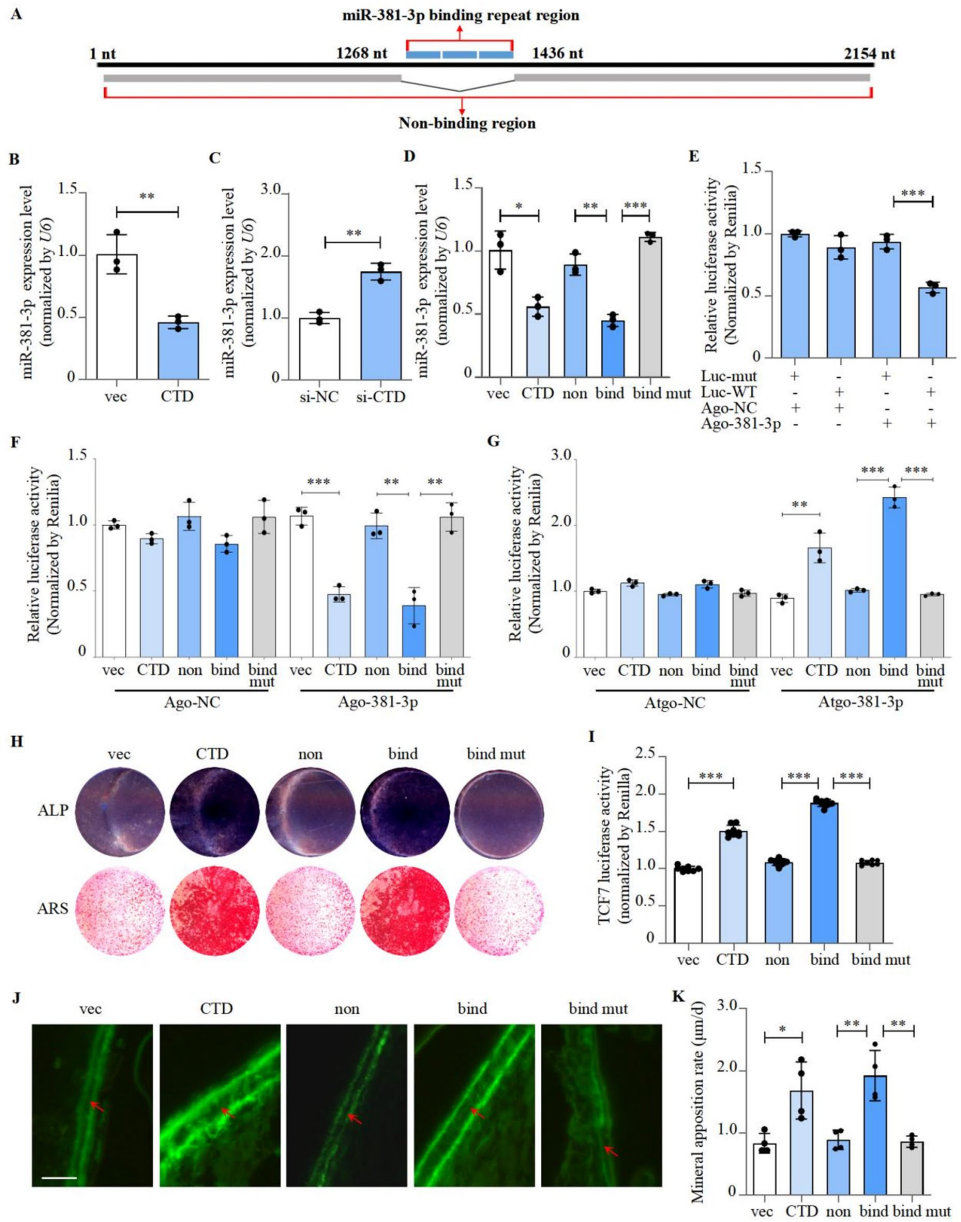
CTD-2555A7.2 bind with multiple miR-381-3p and promoted bone formation. **A** Sequence structure of CTD-2555A7.2.**B** miR-381-3p expression levels of hMSCs treated with CTD-2555A7.2 over-expression plasmid (compared with pCDNA3.1 plasmid), as detected by RT-PCR (mean ± SD, n = 3). vec: blank plasmid. CTD: plasmid containing CTD-2555A7.2 fell length sequence.**C** miR-381-3p expression levels of hMSCs treated with CTD-2555A7.2 siRNA (compared with negative control siRNA, mean ± SD, n = 3).**D** miR-381-3p expression levels of hMSCs treated with CTD-2555A7.2 full length, CTD-2555A7.2 non-binding region, CTD-2555A7.2 binding region and CTD-2555A7.2 mutant binding region over-expression plasmids respectively (compared with pCDNA3.1 plasmid), as detected by RT-PCR (mean ± SD, n = 3). non: plasmid containing CTD-2555A7.2 non-binding region. bind: plasmid containing CTD-2555A7.2 binding region. bind mut: plasmid containing CTD-2555A7.2 mutant binding region.**E** Binding effect of miR-381-3p and CTD-2555A7.2 binding region, as detected by luciferase reporter assay (mean ± SD, n = 3). Luc-mut: pMIR-Report luciferase reporter plasmid containing one mutant miR-381-3p binding repeat sequence of CTD-2555A7.2. Luc-WT: luciferase reporter plasmid containing one wild-type miR-381-3p binding repeat sequence of CTD-2555A7.2. Ago-NC: cells treated with agomiR-NC. Ago-381-3p: cells treated with agomiR-381-3p.**F,G** Binding effect of miR-381-3p and CTD-2555A7.2 different regions (compared with pMIR-Report luciferase reporter plasmid) and treated by agomiR-381-3p (left) or antagomiR-381-3p (right), as detected by luciferase reporter assay (mean ± SD, n = 3). Atgo-NC: cells treated with antagomiR-NC. Atgo-381-3p: cells treated with antagomiR-381-3p.**H** ALP and Alizarin Red staining of hMSC cell treated with CTD-2555A7.2 exprssion plasmids of different regions, as detected by ALP staining and Alizarin Red staining. I. TCF7 activities of hMSCs treated with CTD-2555A7.2 exprssion plasmids of different regions, as detected by luciferase reporter assay (mean ± S.D., n = 8).**J,K** Calvarial bone mineral apposition rates of C57BL/6 mice treated with CTD-2555A7.2 exprssion plasmids of different regions (mean ± SD, n = 4). Scale bar: 10μm.*P < 0.05, **P < 0.01, ***P < 0.001

CTD-2555A7.2 has a unique repeat sequence with three miR-381-3p binding sites (Fig. 2A), sequences were predicted to bind with miR-381-3p by RNA22 V2^32^. We found an inverse correlation between CTD-2555A7.2 and miR-381-3p expression (Fig. 2B-C). No regulatory effect on CTD-2555A7.2 by altering miR-381-3p (Figure S4A-B). Our analysis revealed that the repetitive sequences of CTD-2555A7.2 exhibit binding effects to multiple osteogenic miRNAs. Through RT-PCR assays, we further investigated the regulatory effects of CTD-2555A7.2 on these miRNAs and found that its modulation on miR-381-3p was the most pronounced (Figure S5A-F). We further construct the following plasmids: full-length CTD-2555A7.2 (CTD); non-binding region (non); binding region (bind) and mutated binding region (bind mut). As shown, only plasmids with the binding region could inhibit miR-381-3p (Fig. 2D).

CTD-2555A7.2 sequestered miR-381-3p through the binding sequence. AgomiR-381-3p significantly reduced luciferase activity in cells transfected with a luciferase reporter plasmid containing the binding site but did not affect cells with a mutated site (Fig. 2E). AgomiR-381-3p or antagomiR-381-3p was applied to hMSCs transfected with full-length CTD-2555A7.2 (CTD), non-binding region (non), binding region (bind) and mutated binding region (bind mut). In cells transfected with CTD-2555A7.2 and the binding region, AgomiR-381-3p was able to reduce luciferase activity (Fig. 2F). Similarly, antagomiR-381-3p increased luciferase activity in CTD-2555A7.2 and bound transfected cells, with no effect in other groups (Fig. 2G).

To fully understand this mechanism, we next continued our work with different regions of CTD-2555A7.2. Either the miR-381-3p binding region or the full length of CTD-2555A7.2 could activate osteogenic differentiation and Wnt signaling (Fig. 2H-I; Figure S6A-B). The binding region of CTD-2555A7.2 significantly increased the calvarial mineralization rate (MAR) in mice (Fig. 2J-K). These results provided evidence that the miR-381-3p binding region of CTD-2555A7.2 is required for the promotion of osteoblast differentiation and bone formation.

We then tested whether miR-381-3p was essential in this regulatory mechanism. miR-381-3p knockout (miR-381-3p-KO) hMSCs were constructed using the CRISPR/Cas9 system. Overexpression of CTD-2555A7.2 in miR-381-3p-KO cells failed to induce that effect (Figure S7A-C). Downregulated CTD-2555A7.2 expression also showed no effect on miR-381-3p-KO cells, but we found inhibition of osteogenic differentiation in the control group (Figure S7D-F). CTD-2555A7.2 might be a ceRNA for miR-381-3p and promoted osteogenic differentiation by sequestering miR-381-3p via the binding region.

### miR-381-3p inhibited osteogenic differentiation and bone formation

**Fig. 3:**
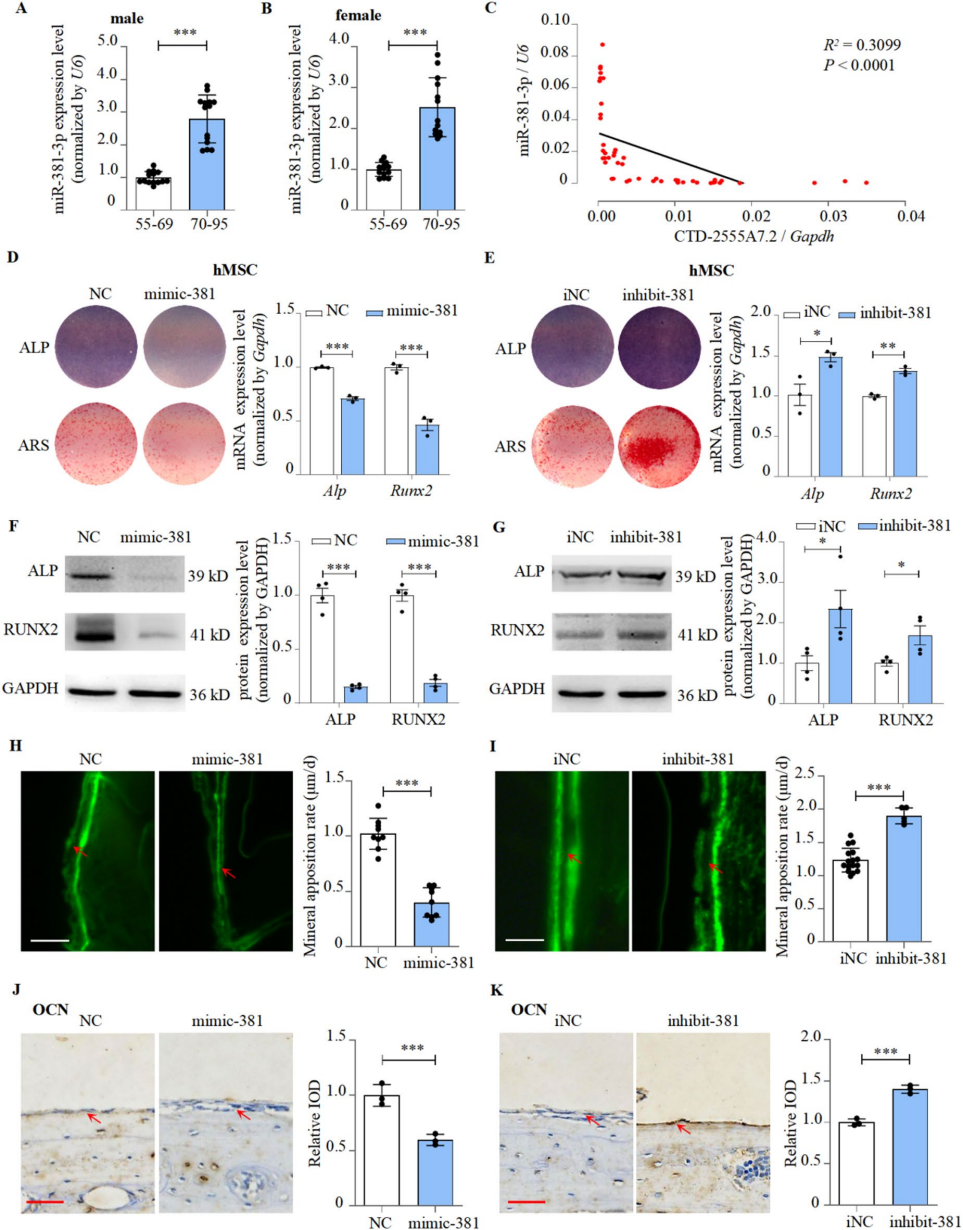
miR-381-3p inhibited osteogenic differentiation and bone formation. **A,B** Expression levels of miR-381-3p in 55-69 and 70-95 years old male (left) and female (right) patients with osteoporosis, as detected by RT-PCR (mean ± S.D., n > 13). **C** Correlation analysis between miR-381-3p level and CTD-2555A7.2 level in bone tissues from osteoporosis patients, as detected by RT-PCR.**D** ALP and Alizarin Red staining of hMSCs treated with mimic-381-3p, as detected by ALP staining and Alizarin Red staining (left), and *Alp*/*Runx2* expression levels of hMSCs treated with mimic-381-3p, as detected by RT-PCR (right, mean ± S.D., n = 3). NC: mimic-NC. mimic-381: mimic-381-3p. **E** ALP and Alizarin Red staining (left), and *Alp*/*Runx2* expression levels (right) of hMSCs treated with inhibitor-381-3p (mean ± S.D., n = 3). iNC: inhibitor-NC. inhibit-381: inhibitor-381-3p. **F** ALP and RUNX2 protein levels of hMSCs treated with mimic-381-3p, as detected by western blot. Representative western blots visualized by enhanced chemiluminescence method (left) and the quantification results with GAPDH as internal control (right, mean ± S.D., n = 4).**G** ALP and RUNX2 protein levels of hMSCs treated with inhibitor-381-3p (mean ± S.D., n = 4).**H,I** Calvarial bone mineral apposition rate of C57BL/6 mice treated with mimic-381-3p (left) or inhibitor-381-3p (right, mean ± S.D., n > 5). Scale bar: 10μm.**J** Expression and quantification of OCN in calvarial tissues of C57BL/6 mice treated with mimic-381-3p, as detected by immunohistochemical staining (left). Quantification of relative integrated optical density (IOD) values of OCN immunostaining using Image-Pro Plus 6.0 software (right, mean ± SD, n = 3). Scale bar: 25μm.**K** Expression and quantification of OCN in calvarial tissues of C57BL/6 mice treated with inhibitor-381-3p (mean ± SD, n = 3).*P < 0.05, **P < 0.01, ***P < 0.001

We investigated the expression of miR-381-3p in the bone tissue of aging osteoporosis patients of different ages. The expression of miR-381-3p was significantly increased with age (Fig. 3A-B). miR-381-3p negatively correlated with CTD-2555A7.2 expression and osteogenic marker genes, such as *Ocn* and *Sp7*, in osteoporosis patients with advanced age (Fig. 3C; S8A-B). miR-381-3p expression significantly increased in ovariectomized (OVX), hind limb unloading (HLU), and aging mice models (Figure S8C-F). Besides, miR-381-3p also showed a negative correlation with osteogenic markers in mice (Figure S8G-H). This suggests a positive link between miR-381-3p and osteoporosis.

We investigated the role of miR-381-3p in osteogenic differentiation by using miR-381-3p mimics or inhibitors in both in vitro (hMSCs and MC3T3-E1 cells) and in vivo systems (Figure S9C-E). After mimic-381-3p transfection, the expression levels of ALP/RUNX2 in the cells were downregulated. At the same time, ALP activity and ARS mineralized nodules were reduced (Fig. 3D, F; Figure S9A). Osteogenic differentiation could also be stimulated by inhibitor-381-3p (Fig. 3E, G; Figure S9B). Transfecting mimic-381-3p into the calvaria of mice significantly reduced the rate of mineral apposition rate and reduced OCN activity (Fig. 3H, J). Interestingly, inhibitor-381-3p increased the rate of that (Fig. 3I, K). Based on these results, miR-381-3p could inhibit osteogenic differentiation and bone formation.

### miR-381-3p regulated bone formation through Wnt signaling pathway

To identify miR-381-3p’s downstream signaling pathways, we analyzed its effect on osteogenic transcription factors. Changes in HES1, SMAD4, and TCF7 were consistent with miR-381-3p inhibiting bone formation (Figure S10A-L). HES1, SMAD4, and TCF7 respectively serves as critical downstream transcription factor of NOTCH, BMP, and Wnt signaling pathways, suggesting these signaling pathways may be affected by miR-381-3p. The result of co-expression analysis in aging osteoporosis patients suggested that miR-381-3p may correlate with the Wnt signaling pathway, but showed no association with either NOTCH or BMP signaling pathways (Figure S10M-O). Therefore, we hypothesized that miR-381-3p regulated bone formation through the Wnt signaling pathway.

**Fig. 4:**
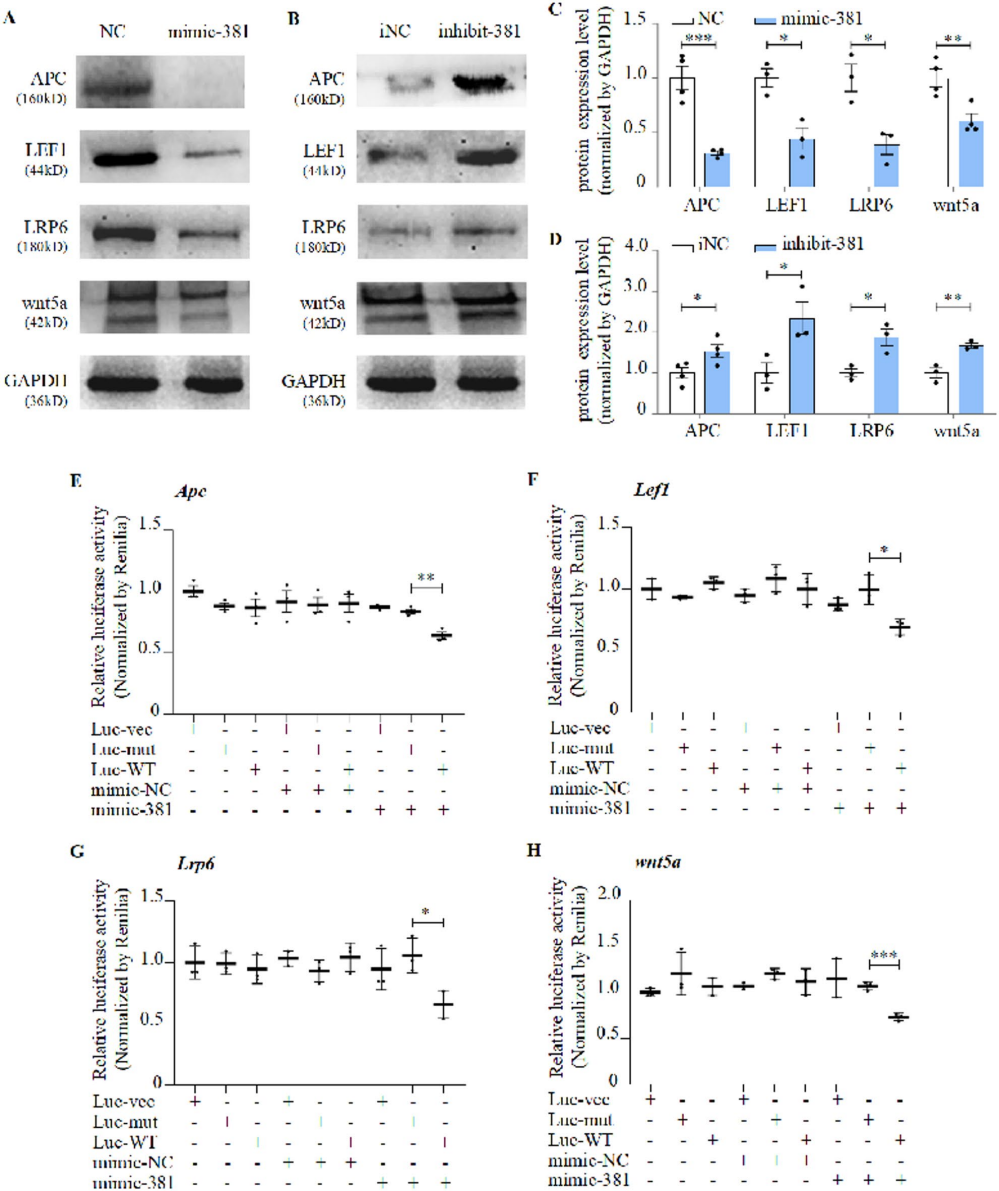
miR-381-3p inhibited four target genes of Wnt signaling pathway. **A, C** Protein expression of APC, LEF1, LRP6 and wnt5a of hMSCs treated with mimic-381-3p (compared with mimic-NC), as detected by western blot (mean ± S.D., n = 3). **B, D** Protein expression of APC, LEF1, LRP6 and wnt5a of hMSCs treated with inhibitor-381-3p, as detected by western blot(mean± S.D., n > 3). **E-H** Binding effect of miR-381-3p and 3′UTR sequences of *Apc*, *Lef1*, *Lrp6* and *wnt5a*, as detected by luciferase reporter assay (mean ± S.D., n = 3). Luc-vec: empty luciferase reporter plasmid. Luc-mut: luciferase reporter plasmid containing mutant 3′UTR. Luc-WT: luciferase reporter plasmid containing wild-type 3′UTR.*P < 0.05, **P < 0.01, ***P < 0.001

We tried to prove the MiRDB prediction that miR-381-3p can target four essential factors in the Wnt pathway: *Apc*, *Lef1*, *wnt5a*, and *Lrp6*. The expression of APC, LEF1, wnt5a, and LRP6 was decreased by mimic-381-3p but increased by inhibitor-381-3p (Fig. 4A-D). The luciferase reporter assay also provided some evidence that miR-381-3p could target these essential factors in the Wnt signaling pathway (Fig. 4E-H; Figure S11A-D).

To verify the relationship between miR-381-3p and Wnt signaling, we also constructed plasmids expressing the 3’-UTR of Apc, Lef1, wnt5a, and Lrp6. After co-transfection of mimic-NC or mimic-381-3p with the 3’-UTR expression plasmids into hMSCs, osteogenic differentiation and TCF7 activity decreased in the mimic-381-3p transfection group (Figure S12A-H). Next, siRNAs targeting these four genes were co-transfected into hMSCs with mimic-381-3p. Mimic-381-3p showed relatively weak regulatory effects on osteogenic differentiation and TCF7 activity, as gene expression was inhibited (Figure S13A-H). These results support our hypothesis that miR-381-3p regulates bone formation through Wnt signaling.

### CTD-2555A7.2 promoted miR-381-3p downstream signalling

We have shown some evidence that miR-381-3p could be regulated by LncRNA CTD-2555A7.2. We are still wondering whether the LncRNA CTD-2555A7.2 could affect Wnt signaling. APC, LEF1, wnt5a, and LRP6 protein levels were increased by CTD-2555A7.2 overexpression and decreased by CTD-2555A7.2 inhibition (Figure S14A-D). This suggests that CTD-2555A7.2 has the same effect as miR-381-3p inhibitor, both promoting the Wnt signaling pathway and regulating osteogenic differentiation.

### nCAR/anti381 and CTD-2555A7.2 binding sequence rescued osteoporosis

Based on our understanding of CTD-2555A7.2 and miR-381-3p, we have tried to find out more about their effect on bone regeneration.

**Fig. 5:**
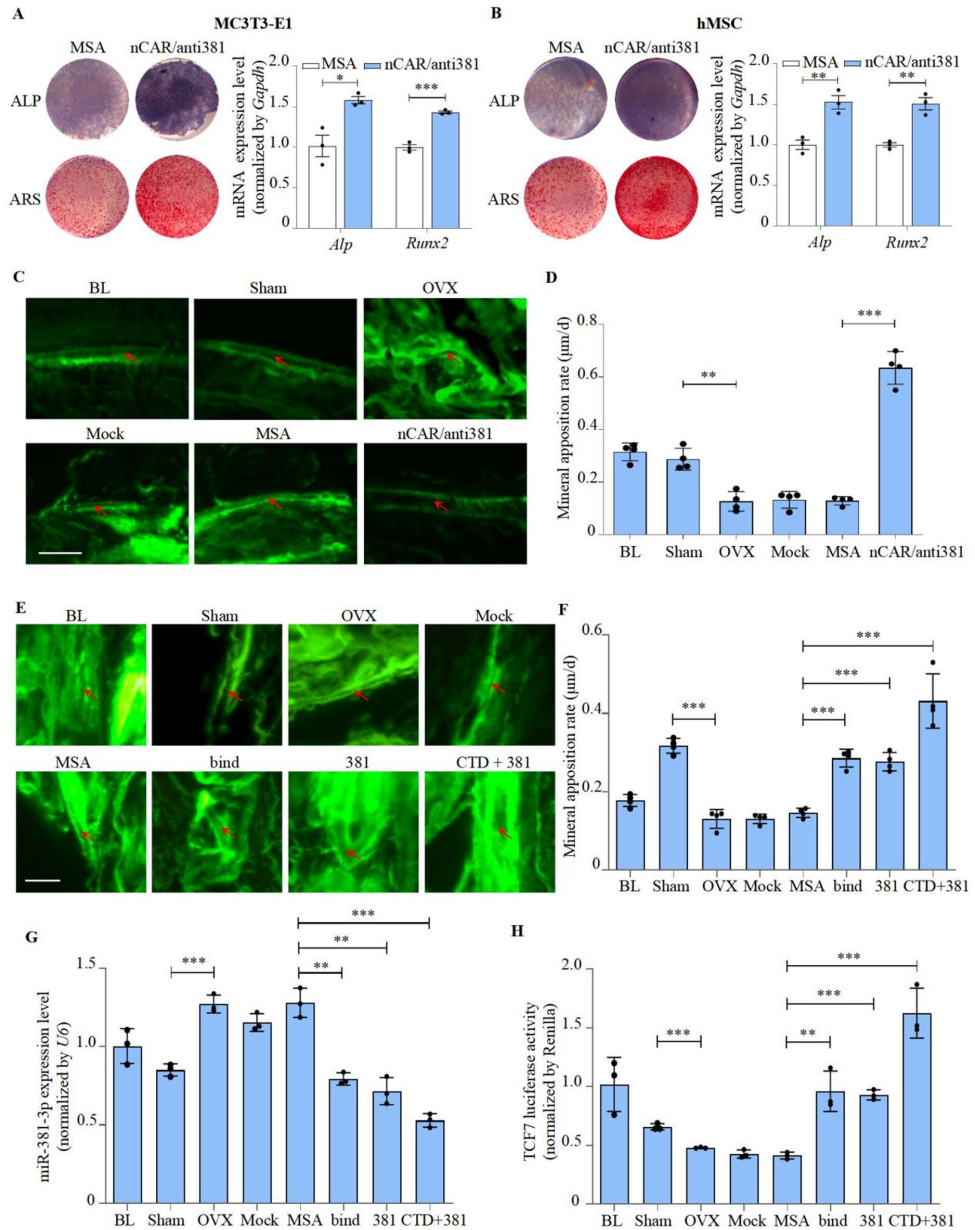
nCAR/anti381 and CTD-2555A7.2 binding sequence rescued osteoporosis. **A** ALP and Alizarin Red staining (left) of MC3T3-E1 cells treated with recombinant miR-381-3p inhibitor (compared with MSA), as detected by ALP staining and Alizarin Red staining, and *Alp*/*Runx2* expression levels of MC3T3-E1 cells treated with recombinant miR-381-3p inhibitor, as detected by RT-PCR (right, mean ± S.D., n = 3). MSA: tRNAMet fused Sephadex aptamer. nCAR/anti381: novel recombinant miR-381-3p inhibitor.**B** ALP and Alizarin Red staining (left), and *Alp*/*Runx2* expression levels (right) of hMSCs treated with recombinant miR-381-3p inhibitor (mean ± S.D., n = 3). **C,D** Femoral trabecular mineral apposition rate of C57BL/6 mice after OVX and recombinant miR-381-3p inhibitor treatment (mean ± S.D., n = 4). BL (Baseline): sacrifice before RNA treatment. Sham: sham OVX operation group. OVX: OVX group. Mock: transfection reagent control group. MSA: empty recombinant tRNA treated group. nCAR/anti381: novel recombinant miR-381-3p inhibitor treated group. Scale bar: 10μm. **E,F** Femoral trabecular mineral apposition rate of C57BL/6 mice after OVX and CTD-2555A7.2 binding sequence and recombinant miR-381-3p inhibitor treatment (mean ± S.D., n = 4). bind: CTD-2555A7.2 binding sequence treated group. 381: novel recombinant miR-381-3p inhibitor treated group. CTD+381: CTD-2555A7.2 binding sequence and recombinant miR-381-3p inhibitor combined treated group. Scale bar: 10μm. **G** Expression levels of miR-381-3p in primary BMSCs of OVX mice treated with CTD-2555A7.2 binding sequence and recombinant miR-381-3p inhibitor, as detected by RT-PCR (mean ± S.D., n = 3).**H** TCF7 activities of primary BMSCs of OVX mice treated with CTD-2555A7.2 binding sequence and recombinant miR-381-3p inhibitor, as detected by luciferase reporter assay (mean ± S.D., n = 3).*P < 0.05, **P < 0.01, ***P < 0.001

Firstly, we synthesised a special bioengineered recombinant miR-381-3p inhibitor, nCAR/anti381. The technique of bioengineered recombinant miRNA inhibitors have been proved on osteoporosis treatment^33^ nCAR/anti381 could promote osteogenic differentiation of hMSC and MC3T3-E1 cell lines (Fig. 5A-B). No hepatic or renal toxicity after femoral periosteal injection of nCAR/anti381 in the OVX osteoporosis mice model of osteoporosis (Figure S15A-C). We found that nCAR/anti381 could increase the mineral apposition rate of OVX mice by 393.2% with increased primary BMSC osteogenic differentiation (Fig. 5C-D; Figure S16A-B). In addition, TCF7 luciferase activity in MC3T3-E1, hMSC, and primary BMSC was significantly increased by nCAR/anti381 (Figure S16C-E). These results also confirmed that nCAR/anti381 promotes bone formation via the Wnt pathway.

Secondly, we tested the function of the synthesized mRNA of the CTD-2555A7.2 binding region on bone formation. We hope it will have a better effect with nCAR/anti381. In vitro, CTD-2555A7.2 binding sequence and nCAR/anti381 could reduce miR-381-3p expression, increase *Alp* and *Runx2* levels as well as elevate TCF7 luciferase activity. Interestingly, using the CTD-2555A7.2 binding sequence and nCAR/anti381 in combination might make this result more obvious than using them separately (Figure S17A-D). In OVX osteoporosis mice, CTD-2555A7.2 binding sequence and nCAR/anti381 were transfected into the femoral marrow cavity, with OVX mice injected with empty recombinant RNA (MSA) as control. No liver or kidney toxicity was observed in any of the mice (Figure S18A-C). The rescue effect and primary osteogenic differentiation of BMSCs were more pronounced in mice treated with the combinatorial treatment compared to those treated with CTD-2555A7.2 binding sequence or nCAR/anti381 (Fig. 5E-H; Figure S19A-B). The above results demonstrated that the CTD-2555A7.2 binding sequence and nCAR/anti381 may be a potential therapeutic agent for osteoporosis.

**Fig. 6:**
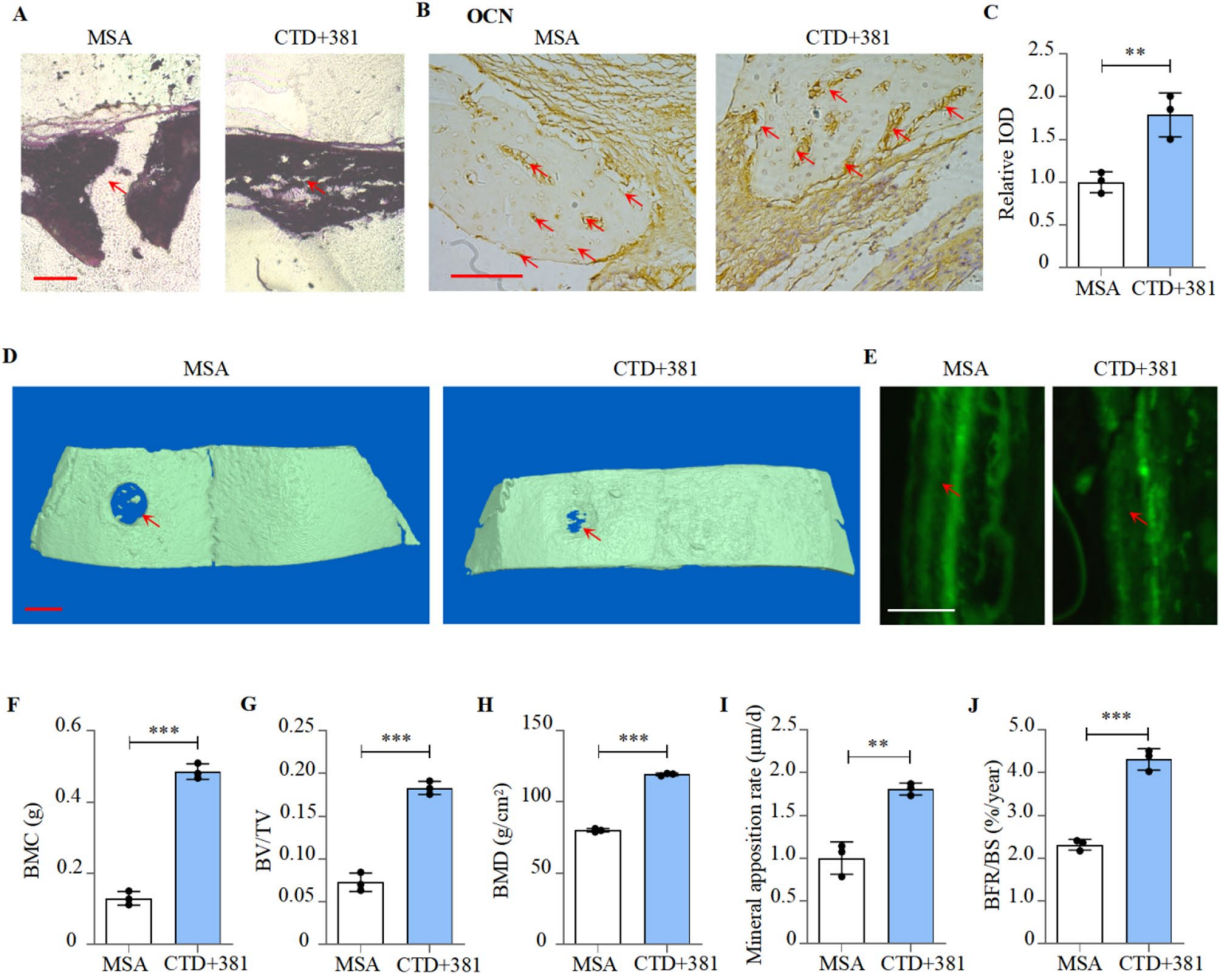
Combination of CTD-2555A7.2 binding sequence and bioengineered recombinant miR-381-3p inhibitor enhanced in situ bone formation of hMSCs. **A** Representative HE staining images of nude mice calvarial defection region implanted with CTD-2555A7.2 binding sequence and recombinant miR-381-3p inhibitor transfected hMSCs. Scale bar: 600 µm.**B,C** Expression of OCN in calvarial defection region of nude mice implanted with CTD-2555A7.2 binding sequence and recombinant miR-381-3p inhibitor transfected hMSCs, as detected by immunohistochemical staining (left). Quantification of relative integrated optical density (IOD) values of OCN immunostaining using Image-Pro Plus 6.0 software (right, mean ± SD, n = 3). Scale bar: 75μm.**D** Representative images showing nude mice calvarial defection region implanted with CTD-2555A7.2 binding sequence and recombinant miR-381-3p inhibitor transfected hMSCs, as detected by micro-CT. Scale bar: 1mm.**E** Representative images showing mineral apposition rate of nude mice calvarial defection region implanted with CTD-2555A7.2 binding sequence and recombinant miR-381-3p inhibitor transfected hMSCs. Scale bar: 10μm.**F-H** Calvarial bone mineral density (BMD), bone volume to tissue volume (BV/TV) and bone mineral content (BMC) of nude mice calvarial defection region implanted with CTD-2555A7.2 binding sequence and recombinant miR-381-3p inhibitor transfected hMSCs (mean ± SD, n = 3).**I,J** Calvarial mineral apposition rate (MAR) and bone formation rate (BFR/BS) of nude mice calvarial defection region implanted with CTD-2555A7.2 binding sequence and recombinant miR-381-3p inhibitor transfected hMSCs (mean ± SD, n = 3).**P < 0.01, ***P < 0.001

Finally, we further investigated the combinatorial therapeutic effect of the CTD-2555A7.2 binding sequence and nCAR/anti381 on the in situ bone formation of hMSCs. A mice calvarial bone defect model was constructed and co-transfected hMSCs were embedded in matrix gel and hydroxyapatite and then implanted at the bone defect site. The healing status of the bone defect site was monitored. One week after treatment, mice implanted with combined transfected hMSCs showed significantly faster collagen fiber coalescence in the bone defect area (Fig. 6A). Following two weeks of treatment, OCN levels around the bone defect were significantly higher compared to the control (Fig. 6B-C). Three weeks after treatment, hMSCs with combined transfection significantly enhanced bone mineral content (BMC), bone volume to tissue volume ratio (BV/TV), and bone mineral density (BMD) in the bone defect site (Fig. 6D, F-H). In addition, the mineral apposition rate (MAR) and bone formation rate (BFR/BS) around the bone defect site were also significantly improved compared to the MSA group (Fig. 6E, I-J).

These results demonstrated the promoting effect of the combinatorial use of CTD-2555A7.2 binding sequence and nCAR/anti381 on the in situ bone formation of hMSCs. They also suggested that the CTD-2555A7.2 binding sequence and nCAR/anti381 may have a stimulating effect on bone regeneration in osteoporotic patients.

## Discussion

The dynamic process of bone resorption and formation is carefully monitored by a sophisticated control system. For years, scientists have struggled to define and explain how it works. The discoveries of the past have not solved the central mystery of the bone: the regulatory mechanisms.

Several LncRNAs could promote osteoblast differentiation. They have great potential in the treatment of osteoporosis. HUVECs-derived exosomes enable transmitting NEAT1 to alleviate inflammation by inducing M2 polarization of macrophages, which finally contributes to osteogenesis^34^. LncRNA GM15416 has an inhibitory effect on the apoptosis of osteoblasts and acts as a preventive factor against osteoporosis^35^. In this study, we reported a new LncRNA, CTD-2555A7.2, promoted osteogenic differentiation and bone formation.

The LncRNA-miRNA network still plays a key role in promoting osteogenic differentiation by controlling osteogenic factors, even though miRNA and its LncRNA counterpart could not completely silence the target gene^36^^,^^37^ LncRNA AK039312 and AK079370 could inhibit bone formation by chelating miR-199b-5p and increasing GSK-3β expression^20^. MALAT1 promoted osteogenic differentiation and inhibited cell apoptosis via the miR-485-5p/WNT7B axis^38^.

The conventional way of LncRNA-miRNA regulation may have subtle changes when the LncRNA has a special repeat sequence. The LncRNA CTD-2555A7.2 sequence provides more binding sites for miR-381-3p due to the 58 nucleotide repeat sequence. Thus, LncRNA CTD-2555A7.2 could bind multiple miR-381-3p simultaneously. The extra-activated miR-381-3p amplifies the signal to regulate osteogenesis, achieving more efficient regulation. Lnc-DIF has also been shown to regulate bone formation more efficiently and precisely through a unique repeat sequence^17^. The same regulatory mechanism also applies to CTD-2555A7.2, making the regulation of osteoporosis even more efficient and precise.

MicroRNAs (miRNAs) play an essential role in the post-transcriptional regulation of genes. Unfortunately, our understanding of the complex mechanisms underlying miRNA-target interactions remains patchy. Their cellular regulatory effect is determined not only by their number or expression levels but also by the regulatory efficacy of individual miRNAs^39^. Instead of binding only one target, miR-30-5p and miR-12200-5p could regulate osteogenic differentiation more efficiently by binding to multiple factors in the Wnt pathway simultaneously^40^^,^^41^ We found another example that regulates osteogenic differentiation in the same way: miR-381-3p could simultaneously target four important factors in the Wnt signaling, including *wnt5a*, *Apc*, *Lrp6*, and *Lef1*. When we comprehensively analyzed the results of CTD-2555A7.2 and miR-381-3p, we were surprised to find that they can form a synergistic regulatory effect in multiple ways: achieving a dual enhancement effect in promoting bone formation.

As LncRNAs could act as potential new RNA drug candidates, they could aid in the diagnosis, treatment, and prognosis of disease. Scientists are constantly trying to find a way to target LncRNAs or develop LncRNA-based drugs. Unlike mRNA, most LncRNAs function in a folded conformation. Conservation of the 3D structure appears to be more important for LncRNA function than sequence conservation^42^. However, challenges remain, including low interspecies homology and unknown safety and efficacy risks^43^. We are still trying to find a new path for highly specific, highly effective, and long-lasting drugs based on LncRNAs. We are using the repeat sequence of CTD-2555A7.2, which binds with miR-381-3p, as a potential RNA drug to regulate bone formation and obtain enhanced rescue effects in osteoporotic mice. Our result also showed that by using the key sequence of the human LncRNA, the low interspecies homology of the LncRNA may not be a problem at all.

To summarize, we have shown that CTD-2555A7.2 can bind to multiple miR-381-3p with a special repeat sequence. miR-381-3p targets multiple genes in the Wnt signaling pathway and thereby promotes bone formation through a dual amplification regulation pattern. We also investigated the therapeutic potential of the repeat sequence of CTD-2555A7.2 for osteoporosis. This study proposed novel perspectives for further investigating the mechanism of osteogenic differentiation and verified the possibility of RNA therapeutics in preventing and treating osteoporosis. Understanding the intricate roles of LncRNAs in osteoporosis not only elucidates disease mechanisms but also opens avenues for innovative diagnostics and targeted therapies. This study explores the regulatory landscape of LncRNAs in osteoporosis, aiming to identify novel biomarkers and therapeutic strategies to counteract bone loss.

## Electronic supplementary material

Below is the link to the electronic supplementary material.

Supplementary Material 1

Supplementary Material 2

Supplementary Material 3

Supplementary Material 4

Supplementary Material 5

Supplementary Material 6

Supplementary Material 7

Supplementary Material 8

Supplementary Material 9

Supplementary Material 10

Supplementary Material 11

Supplementary Material 12

Supplementary Material 13

Supplementary Material 14

Supplementary Material 15

Supplementary Material 16

Supplementary Material 17

Supplementary Material 18

Supplementary Material 19

Supplementary Material 20

Supplementary Material 21

Supplementary Material 22

Supplementary Material 23

Supplementary Material 24

## Data Availability

The datasets used and/or analysed during the current study available from the corresponding author [Chong Yin, yinchong42@nsmc.edu.cn] on reasonable request.
