# Supplementary material for: LncRNA CTD-2555A7.2 promotes bone formation with LncRNA-specific cascade amplification strategy
Source: Sci Rep. 2025 Jul 1;15:20687. doi: 10.1038/s41598-025-05826-z (PMC12217230; doi:10.1038/s41598-025-05826-z)
Supplement: Supplementary file 1 — Supplementary Material 1 [file 41598_2025_5826_MOESM1_ESM.docx]

**Table 1 Primers Sequences for qRT-PCR**

| **Target gene** | **Sequences (5’→3’)** |
| --- | --- |
| Human *Alp*-Forward | GGCCATTGGCACCTGCCTTA |
| Human *Alp*-Reverse | ACCCATCCCATCTCCCAGGAA |
| Human *Ocn*-Forward | GGTGCAGCCTTTGTGTCCAAGC |
| Human *Ocn*-Reverse | GTCAGCCAACTCGTCACAGTCC |
| Human *Sp7*-Forward | CCTCTGCGGGACTCAACAAC |
| Human *Sp7*-Reverse | AGGGTGGGTAGTCATTTGCAT |
| Human *Gapdh*-Forward | CATGGAGAAGGCTGGGGCTC |
| Human *Gapdh*-Reverse | CACTGACACGTTGGCAGTGG |
| Mouse *Alp*-Forward | GTTGCCAAGCTGGGAAGAACAC |
| Mouse *Alp*-Reverse | CCCACCCCGCTATTCCAAAC |
| Mouse *Runx2*-Forward | CGCCCCTCCCTGAACTCT |
| Mouse *Runx2*-Reverse | TGCCTGCCTGGGATCTGTA |
| Mouse *Gapdh*-Forward | TGCACCACCAACTGCTTAG |
| Mouse *Gapdh*-Reverse | GGATGCAGGGATGATGTTC |
| CTD-2555A7.2-Forward | TGCCTTCACCTCTGCTCTTC |
| CTD-2555A7.2-Reverse | AGGGTGGGTTGAGGTAGAGG |
| Human *Tcf7*-Forward | GCGGACATCAGCCAGAAG |
| Human *Tcf7*-Reverse | TCACAGTATGGGGGAGCTGT |
| Human *Smad4*-Forward | GCCGCATGAAGTGGATAAGG |
| Human *Smad4*-Reverse | TCTGGTCCGCGTGTTCTTT |
| hsa-miR-381-3p-RT | GTCGTATCCAGTGCAGGGTCCGAGGTATTCGCACTGGATACGACACAGAG |
| hsa-miR-381-3p-Forward | CGCGTATACAAGGGCAAGCT |
| hsa-miR-381-3p-Reverse | CCAGTGCAGGGTCCGAGGT |
| hsa-miR-15b-5p-RT | GTCGTATCCAGTGCAGGGTCCGAGGTATTCGCACTGGATACGACAGCCAT |
| hsa-miR-15b-5p-Forward | TAGCAGCACAATAGTGT |
| hsa-miR-15b-5p-Reverse | CCAGTGCAGGGTCCGAGGT |
| hsa-miR-21-5p-RT | GTCGTATCCAGTGCAGGGTCCGAGGTATTCGCACTGGATACGACTCAGAC |
| hsa-miR-21-5p-Forward | TAGCTTATCAGACTGATG |
| hsa-miR-21-5p-Reverse | CCAGTGCAGGGTCCGAGGT |
| hsa-miR-146a-5p-RT | GTCGTATCCAGTGCAGGGTCCGAGGTATTCGCACTGGATACGACACCAGT |
| hsa-miR-146a-5p-Forward | TGAGAACTGAATTCCAT |
| hsa-miR-146a-5p-Reverse | CCAGTGCAGGGTCCGAGGT |
| Mouse *Hes1*-Forward | CCAGCCAGTGTCAACACGA |
| Mouse *Hes1*-Reverse | AATGCCGGGAGCTATCTTTCT |
| Mouse *Tcf7*-Forward | CAGAATCCACAGATACAGCA |
| Mouse *Tcf7*-Reverse | CAGCCTTTGAAATCTTCATC |
| Mouse *Smad4*-Forward | TGCCTGGTCTGTGTGTTTGG |
| Mouse *Smad4*-Reverse | GCCACGGAGTTGTCATAGGA |
| Human *Apc*-Forward | TGACCTCCACTCGCCTGTCTTG |
| Human *Apc*-Reverse | GCCTTCTCGTTAGCCCTGAATGTC |
| Human *Lef1*-Forward | CTTTATCCAGGCTGGTCTGC |
| Human *Lef1*-Reverse | TCGTTTTCCACCATGTTTCA |
| Human *Lrp6*-Forward | GCTTCTGCGTGCTGCTGAGAG |
| Human *Lrp6*-Reverse | CCTCCAAGCCTCCAACTACAATCG |
| Human *wnt5a*-Forward | CAGGCGGTGGCAAGCAGAAC |
| Human *wnt5a*-Reverse | AGTGTGGGCAGGCAGTGGTC |
| LA16c-306A4.2-Forward | CAGGTGCTCAAGGTCTTCCT |
| LA16c-306A4.2-Reverse | TGGCTGAGACTGCTGTGATT |
| HP09025-Forward | GCTCCATGGAGACCTCAAGA |
| HP09025-Reverse | CAGGCTGTGTGGTTTGCTTT |
| TP73-AS1-Forward | GGGAAATACCCGACCTCAAC |
| TP73-AS1-Reverse | TCCAGGTCCCAAGTTCTTCC |
| AP001476.3-201-Forward | GCTGGGACTTCTGGACTGTG |
| AP001476.3-201-Reverse | CCAGGGTCTGGTGTCTGTTC |
| RP11-405M12.2-Forward | CACAGCCACCTCTTCCTCTC |
| RP11-405M12.2-Reverse | TGCTGAGGAAGGTGAGGAAG |

**Table 2 siRNA, inhibitor and mimic**

| **Target gene** | **Sequences (5’→3’)** |
| --- | --- |
| si-CTD-2555A7.2 | GGAUAGAGAGGGAAACUGAGG |
| si-wnt5a | CGGATAACCTTGTAACATATT |
| si-LRP6 | CCAAACTACAAGCCCTGCACTT |
| si-APC | GAATAAACATCTCCGTGAATT |
| si-LEF1 | GUGAAGAGCAGGCUAAAUATT |
| mmu-miR-381-3p mimics sense | UAUACAAGGGCAAGCUCUCUGU |
| mmu-miR-381-3p mimics antisense | AGAGAGCUUGCCCUUGUAUAUU |
| mmu-miR-381-3p inhibitor sense | ACAGAGAGCUUGCCCUUGUAUA |

**
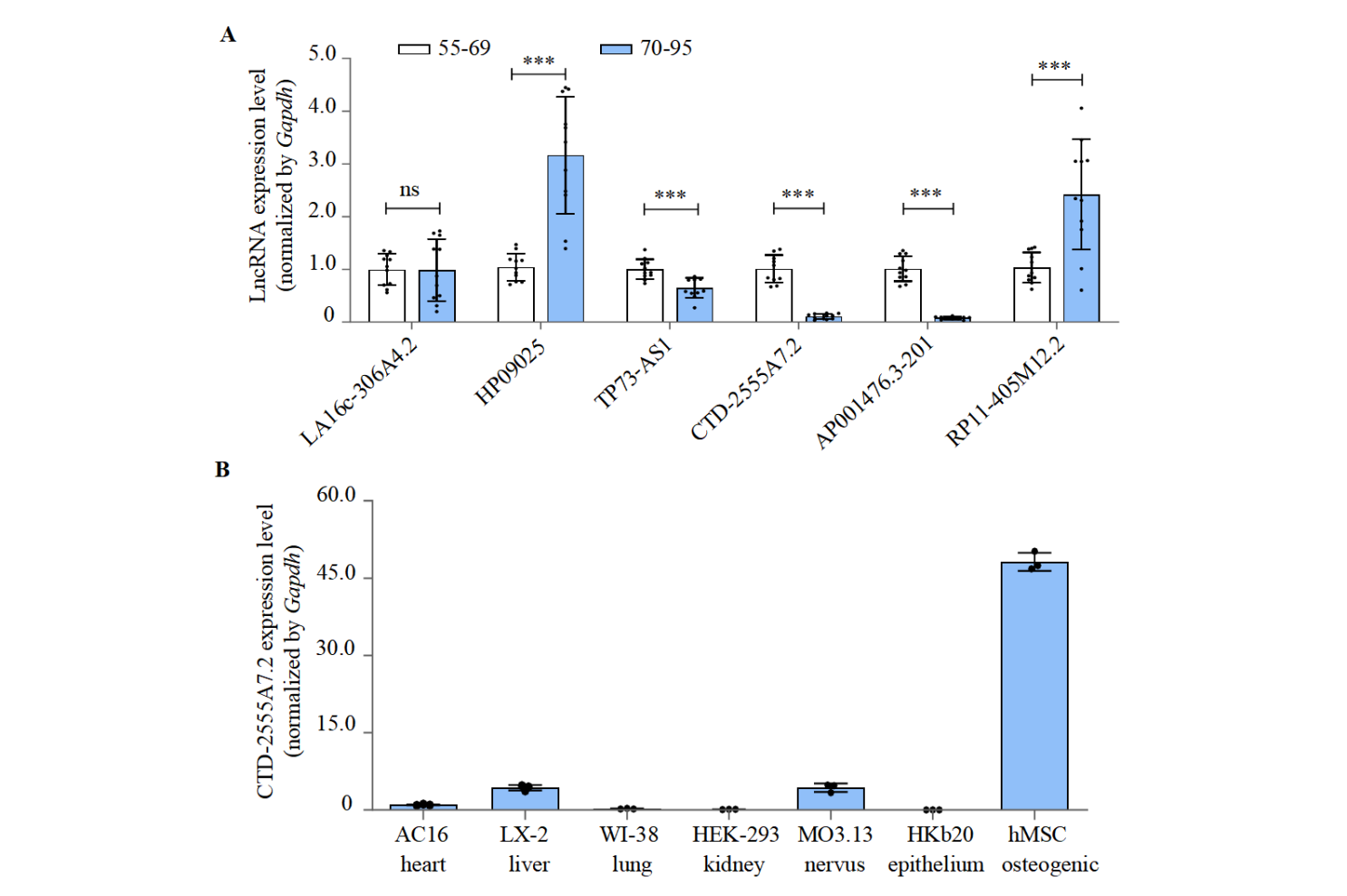
**

**Supplemental Figure 1. Screening of CTD-2555A7.2 in clinical samples**

A. Expression levels of multiple LncRNAs in bone tissue samples of elderly osteoporosis patients, as detected by RT-PCR (mean ± S.D., ****P* < 0.001, n > 3). 55-69: 55-69 years old osteoporosis patients. 70-95: 70-95 years old osteoporosis patients.

B. CTD-2555A7.2 levels across multiple cell types in human tissues were detected, including AC16, LX-2, WI-38, HEK-293, MO3.13, HKb20 and hMSC (n > 3).

**
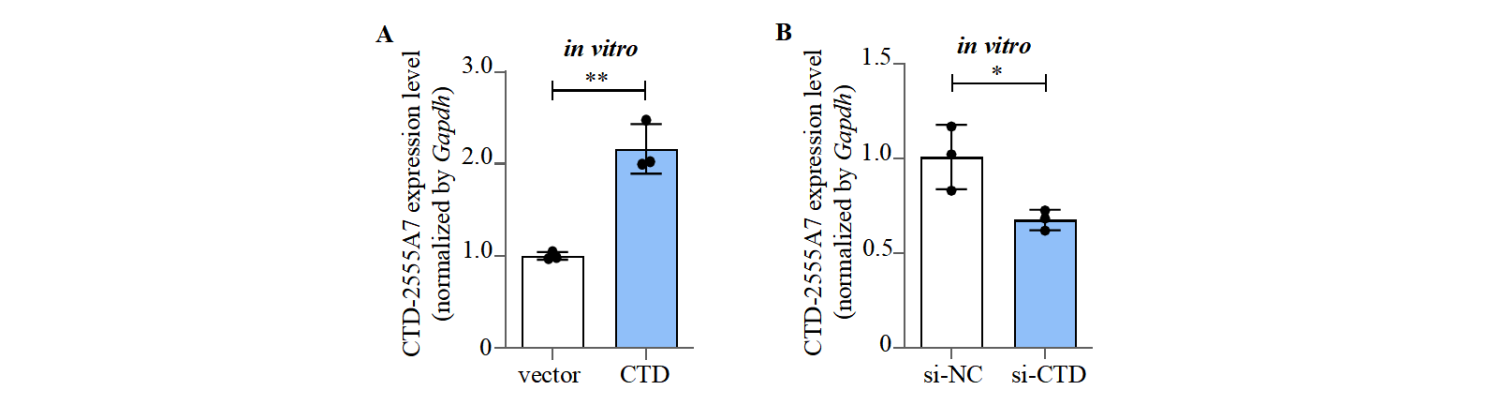
**

**Supplemental Figure 2. Effects of CTD-2555A7.2 over-expression plasmid and CTD-2555A7.2 siRNA**

A. CTD-2555A7.2 expression levels of hMSCs treated with CTD-2555A7.2 over-expression plasmid (compared with pCDNA3.1 plasmid), as detected by RT-PCR (mean ± SD, ***P* < 0.01, n = 3). vector: pCDNA3.1 plasmid. CTD: plasmid containing CTD-2555A7.2 full length.

B. CTD-2555A7.2 expression levels of hMSCs treated with CTD-2555A7.2 siRNA (compared with negative control siRNA, mean ± SD, **P* < 0.05, n = 3). si-NC: negative control siRNA. si-CTD: CTD-2555A7.2 siRNA.

**
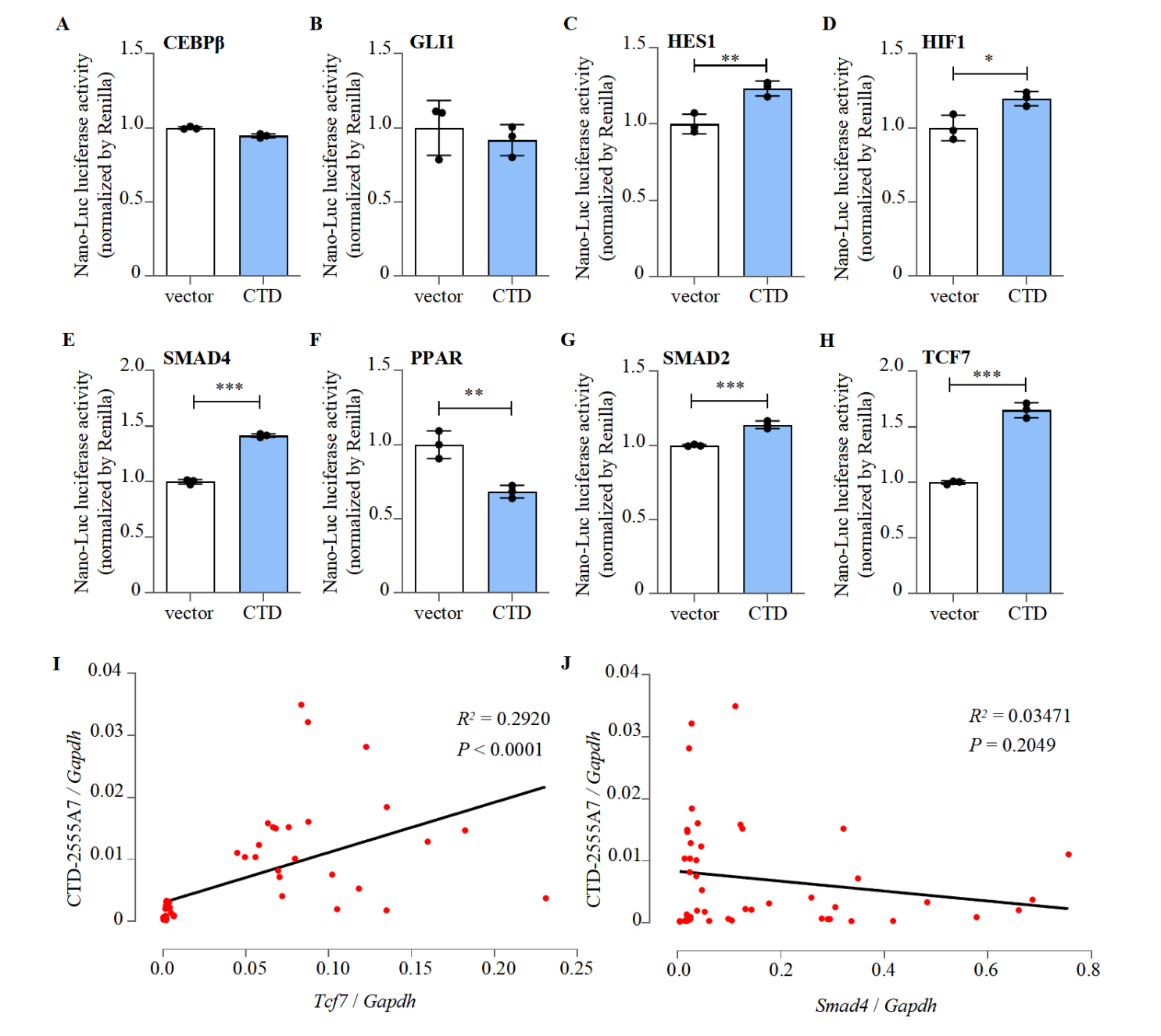
**

**Supplemental Figure 3. Screening of CTD-2555A7.2 downstream signaling pathway**

A-H. CEBPβ/ GLI1/ HES1/ HIF1/ SMAD4/ PPAR/ SMAD2/ TCF7 activities of hMSCs treated with CTD-2555A7.2 over-exprssion plasmid (compared with pCDNA3.1 plasmid), as detected by luciferase reporter assay (mean ± S.D., **P* < 0.05, ***P* < 0.01, ****P* < 0.001, n = 3). vector: pCDNA3.1 plasmid. CTD: CTD-2555A7.2 over-exprssion plasmid.

I-J. Correlation analysis between CTD-2555A7.2 level and *Tcf7* or *Smad4* mRNA levels in bone tissues from osteoporosis patients, as detected by RT-PCR.

**
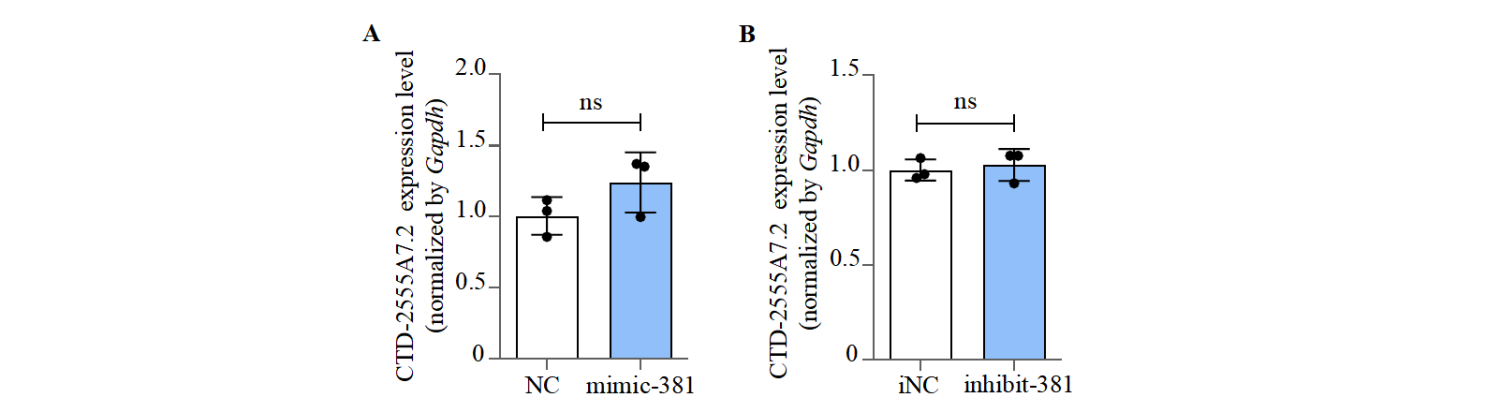
**

**Supplementary Figure 4. The regulation effect of miR-381-3p on CTD-2555A7.2**

A-B. CTD-2555A7.2 expression levels of hMSCs treated with mimic-381-3p (left) or inhibitor-381-3p (right), as detected by RT-PCR (mean ± SD, n = 3). NC: mimic-NC. mimic-381: mimic-381-3p. iNC: inhibitor-NC. inhibit-381: inhibitor-381-3p.

**
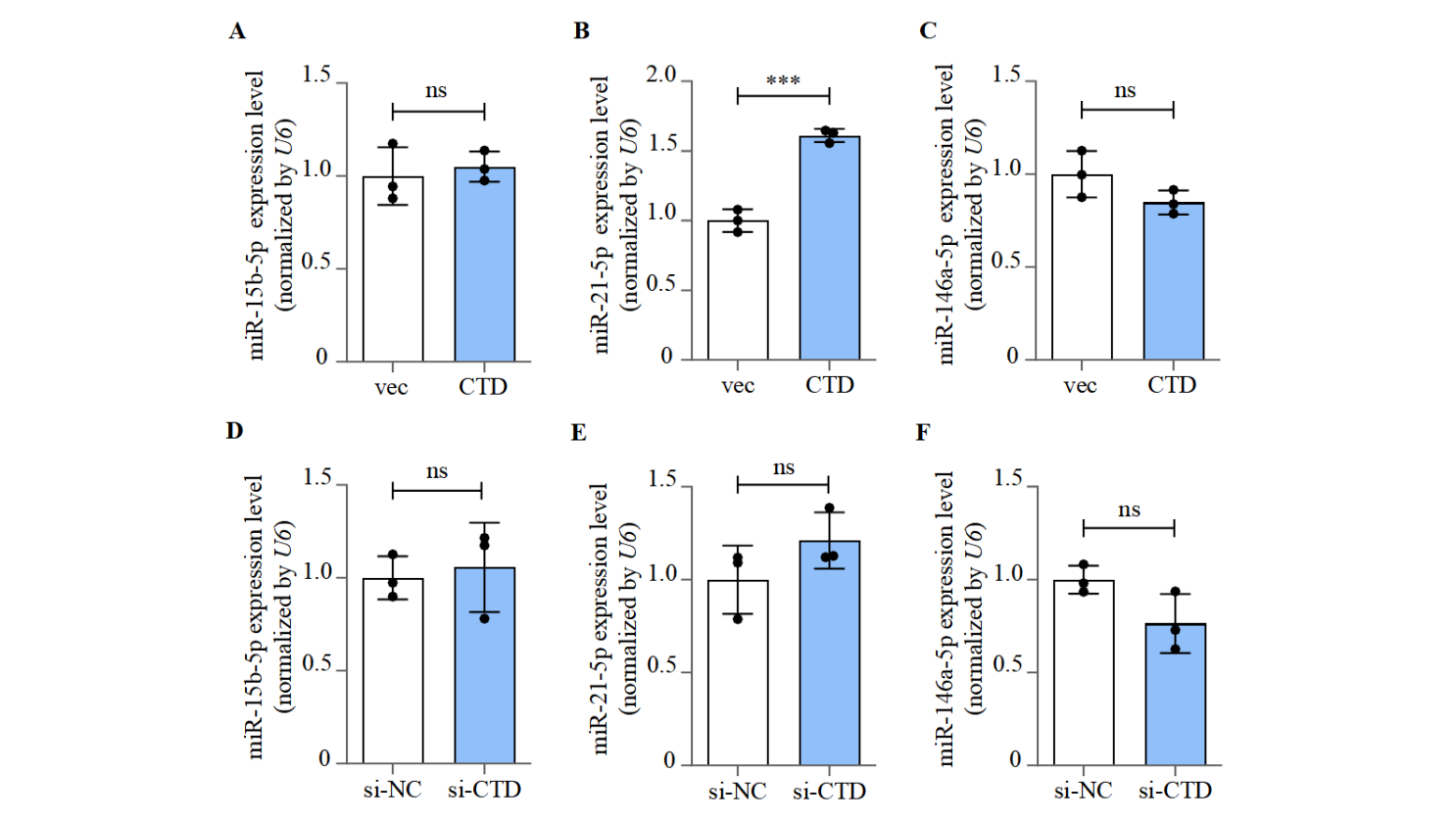
**

**Supplementary Figure 5. The regulatory role of CTD-2555A7.2 in other miRNAs**

A-C. miR-15b-5p, miR-21-5p and miR-146a-5p expression levels of hMSCs treated with CTD-2555A7.2 over-expression plasmid (compared with pCDNA3.1 plasmid), as detected by RT-PCR (mean ± SD, n = 3). vec: blank plasmid. CTD: plasmid containing CTD-2555A7.2 fell length sequence.

D-F. miR-15b-5p, miR-21-5p and miR-146a-5p expression levels of hMSCs treated with CTD-2555A7.2 siRNA (compared with negative control siRNA, mean ± SD, ****P* < 0.001, n = 3).

**
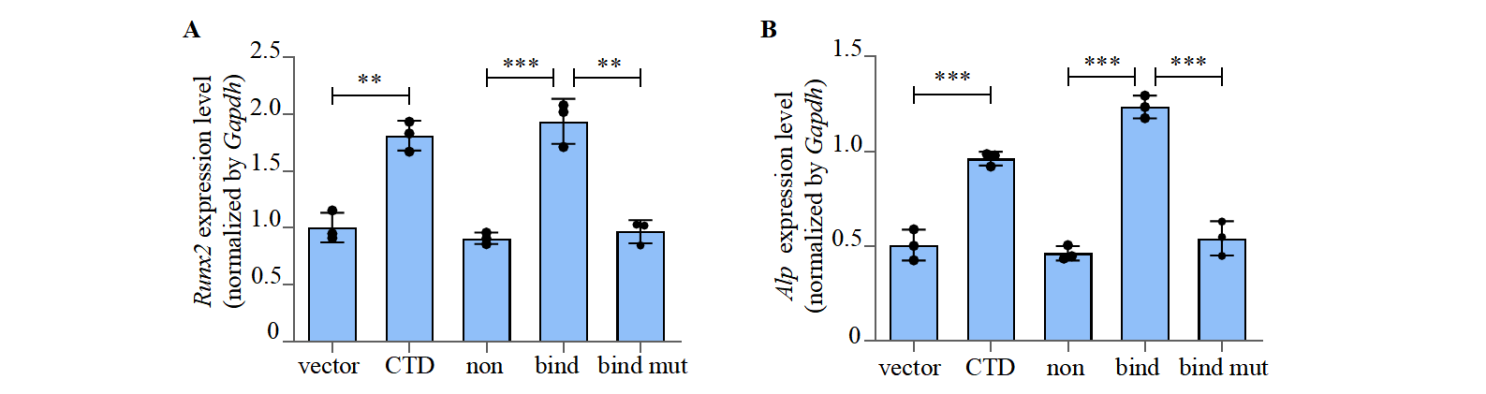
**

**Supplementary Figure 6. CTD-2555A7.2 binding region promoted osteogenic differentiation genes**

A-B. *Runx2* and *Alp* expression levels of hMSCs treated with CTD-2555A7.2 expression plasmids of different regions, as detected by RT-PCR (mean ± SD, ***P* < 0.01, ****P* < 0.001, n = 3). vec: pCDNA3.1 plasmid. CTD: plasmid containing CTD-2555A7.2 full length. non: plasmid containing CTD-2555A7.2 non-binding region. bind: plasmid containing CTD-2555A7.2 binding region. bind mut: plasmid containing CTD-2555A7.2 mutant binding region.

**
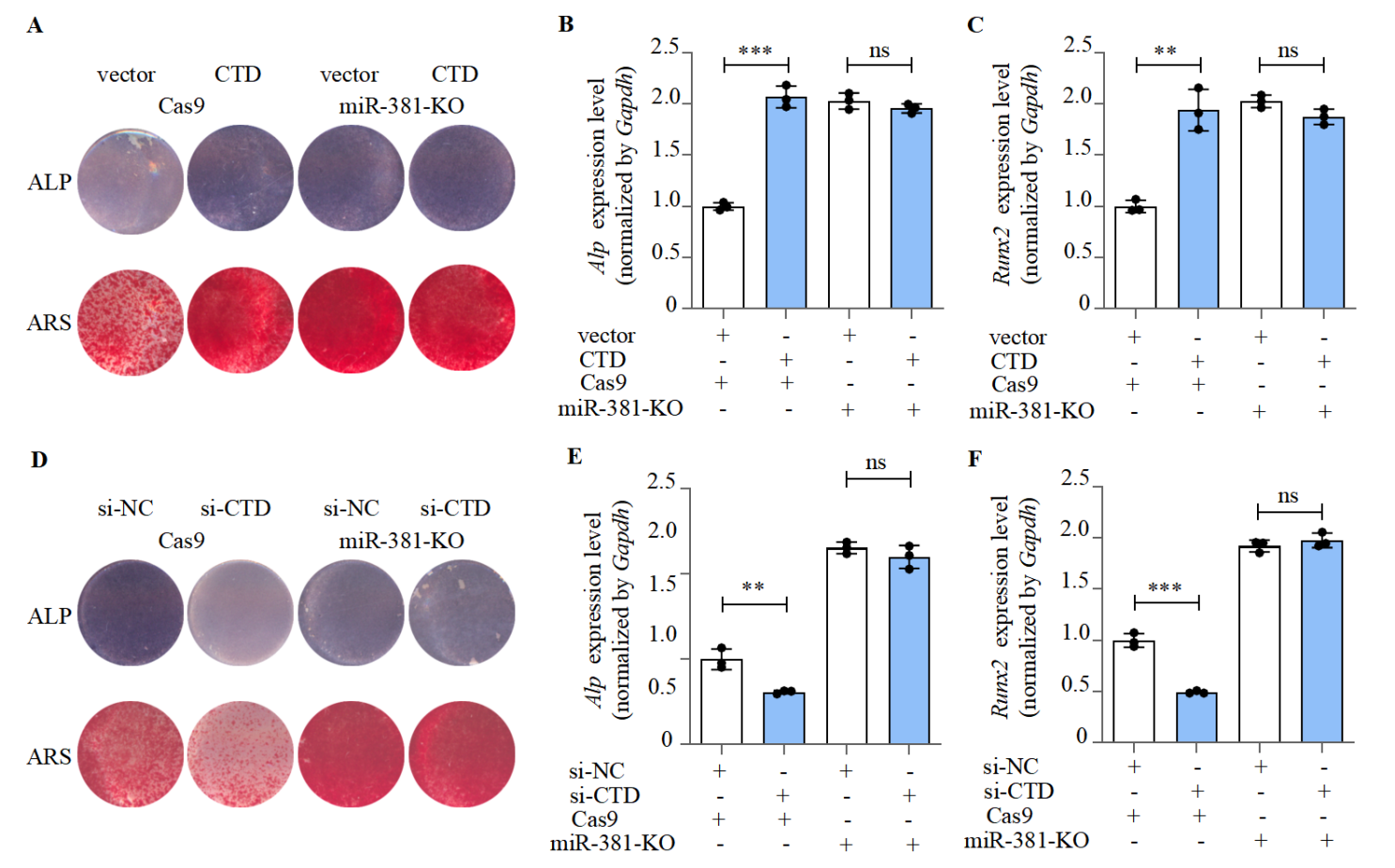
**

**Supplementary Figure 7. CTD-2555A7.2 promoted osteogenic differentiation through miR-381-3p**

A. ALP and Alizarin Red staining of miR-381-3p knock out hMSCs treated with CTD-2555A7.2 over-expression plasmids, as detected by ALP staining and Alizarin Red staining.

B-C. *Alp* and *Runx2* expression levels of miR-381-3p knock out hMSCs treated with CTD-2555A7.2 over-expression plasmids, as detected by RT-PCR (mean ± SD, n = 3).

D. ALP and Alizarin Red staining of miR-381-3p knock out hMSCs treated with CTD-2555A7.2 siRNA.

E-F. *Alp* and *Runx2* expression levels of miR-381-3p knock out hMSCs treated with CTD-2555A7.2 siRNA (mean ± SD, n = 3). ***P* < 0.01, ****P* < 0.001

**
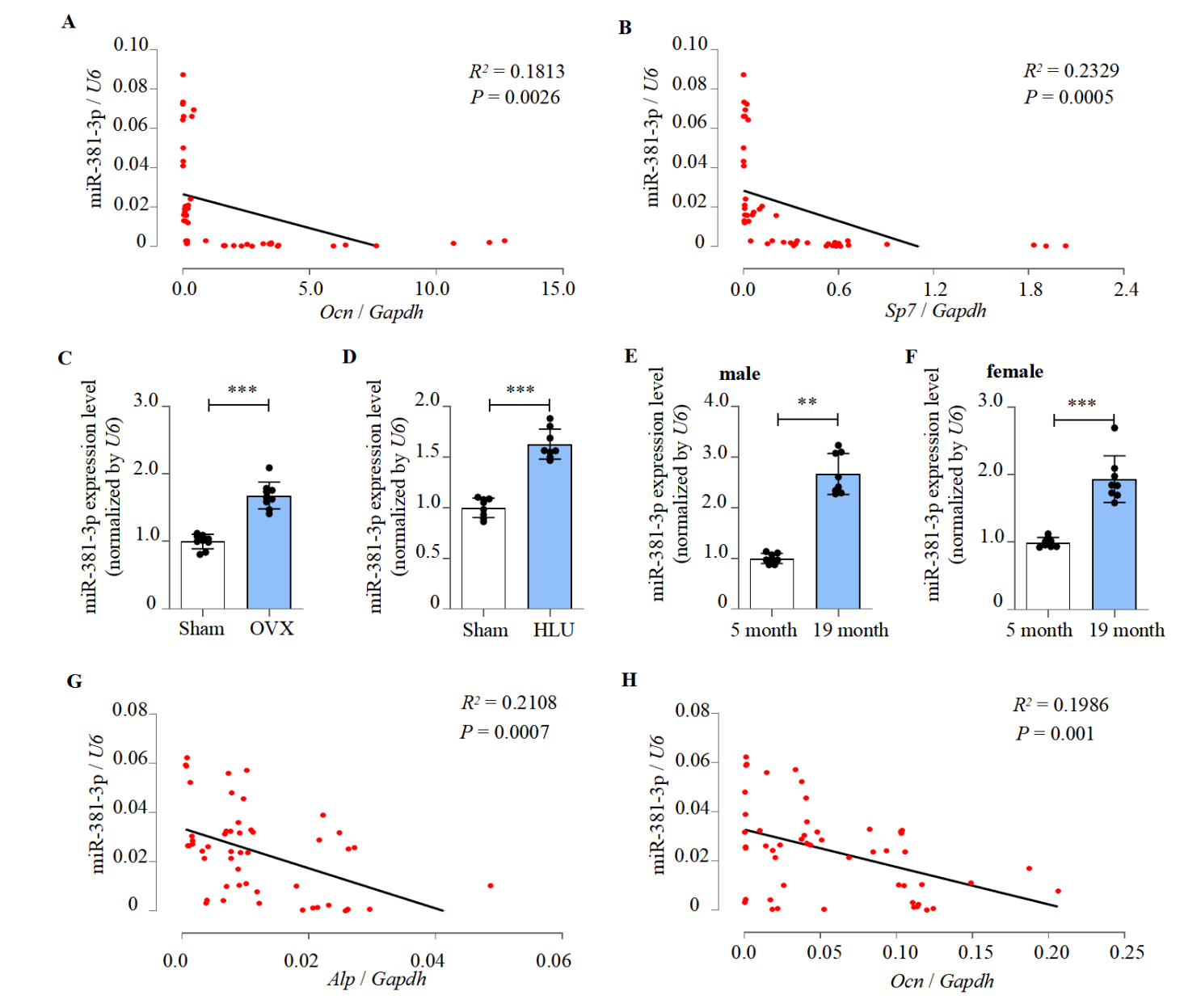
**

**Supplementary Figure 8. miR-381-3p is positively correlated with osteoporosis**

A-B. Correlation analysis between miR-381-3p level and *Ocn*/*Sp7* level in bone tissues from osteoporosis patients, as detected by RT-PCR.

C. Expression levels of miR-381-3p in primary BMSCs of ovariectomized (OVX) and control C57BL/6 mice, as detected by RT-PCR (mean ± S.D., n = 9).

D. Expression levels of miR-381-3p in primary BMSCs of hind limb unloaded (HLU) and control C57BL/6 mice (mean ± S.D., n = 8).

E-F. Expression levels of miR-381-3p in primary BMSCs of 19 month old and 5 month old male (left) or female (right) C57BL/6 mice (mean ± S.D., n = 8).

G-H. Correlation analysis between miR-381-3p level and *Alp* or *Ocn* mRNA levels in C57BL/6 mice, as detected by RT-PCR. ***P* < 0.01, ****P* < 0.001

**
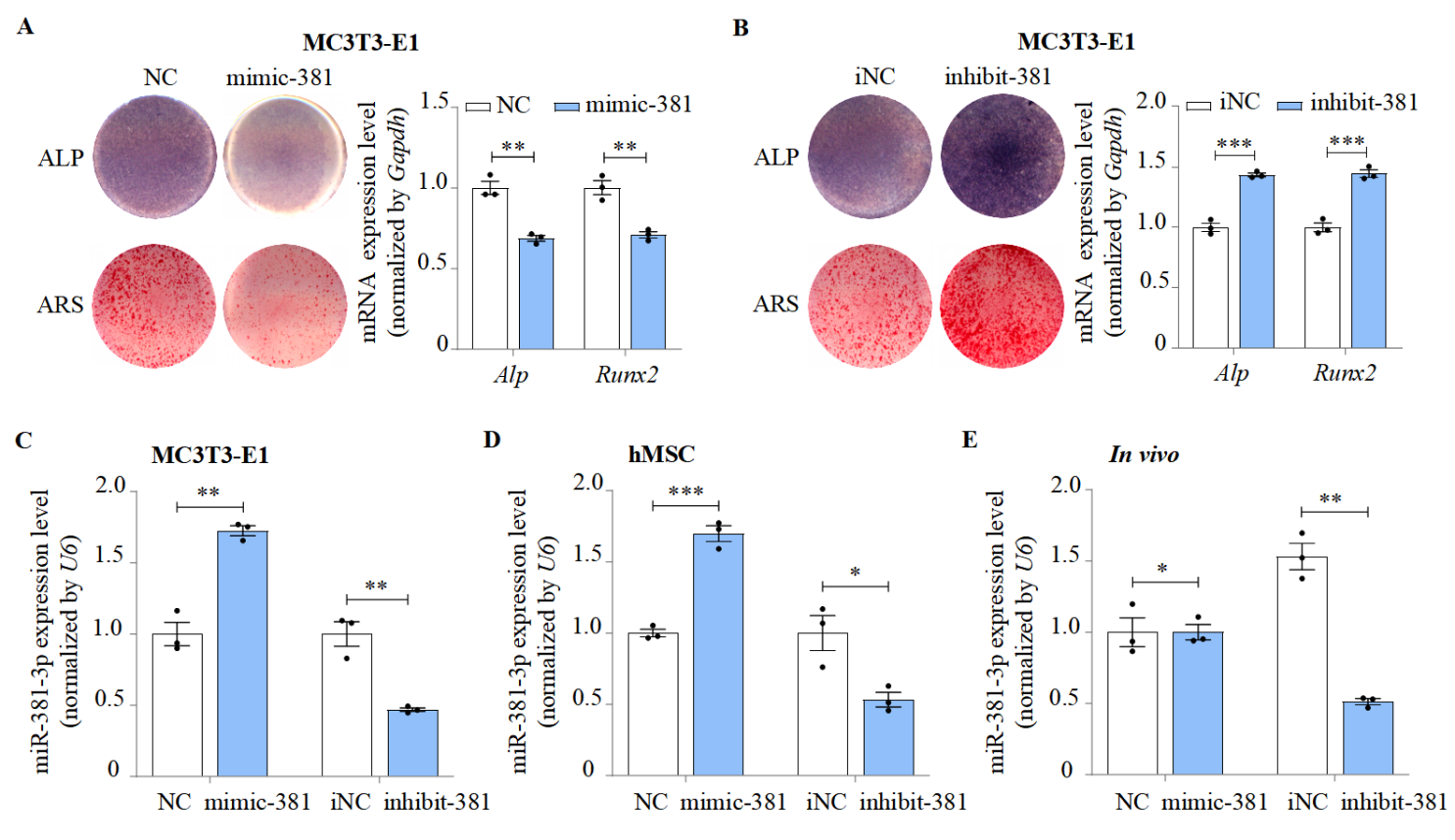
**

**Supplementary Figure 9. miR-381-3p inhibited osteogenic differentiation in MC3T3-E1 cells**

A. ALP and Alizarin Red staining of MC3T3-E1 cells treated with mimic-381-3p (compared to mimic-NC), as detected by ALP staining and Alizarin Red staining (left), and *Alp*/*Runx2* expression levels of MC3T3-E1 cells treated with mimic-381-3p, as detected by RT-PCR (right, mean ± S.D., n = 3).

B. ALP and Alizarin Red staining (left), and *Alp*/*Runx2* expression levels (right) of MC3T3-E1 cells treated with inhibitor-381-3p (mean ± S.D., n = 3).

C-E. *In vitro* and *in vivo* miR-381-3p expression levels following treatment with miR-381-3p mimic and inhibitor separately, as detected by RT-PCR (mean ± SD, n = 3). **P* < 0.05, ***P* < 0.01, ****P* < 0.001

**
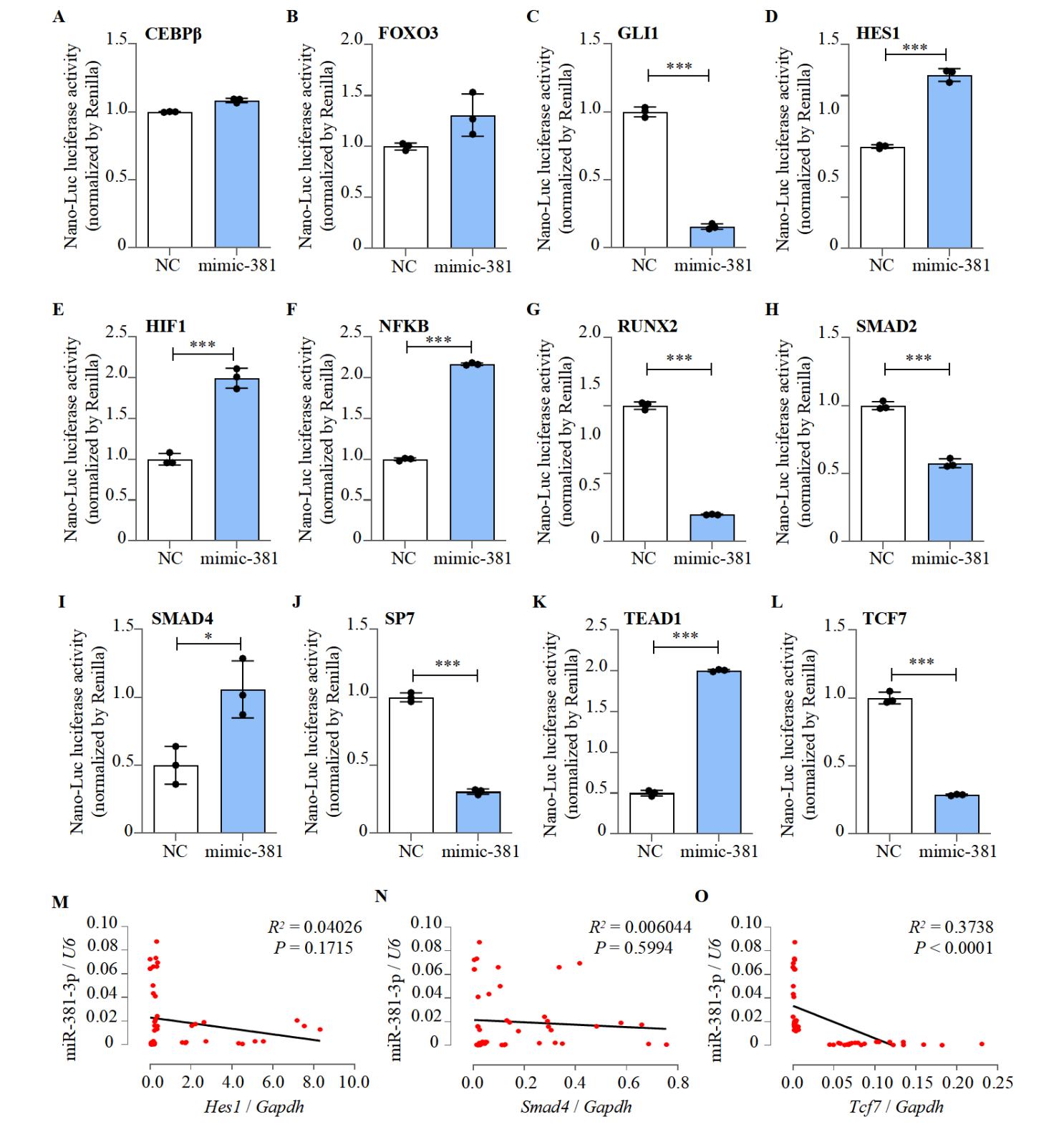
**

**Supplementary Figure 10. miR-381-3p inhibited Wnt signaling pathway**

A-L. CEBPβ/ FOXO3/ GLI1/ HES1/ HIF1/ NFKB/ RUNX2/ SMAD2/ SMAD4/ SP7/ TEAD1/ TCF7 activities of hMSCs treated with mimic-381-3p (compared with mimic-NC), as detected by luciferase reporter assay (mean ± S.D., **P* < 0.05, ****P* < 0.001, n = 3).

M-O. Correlation analysis between miR-381-3p level and *Hes1*, *Smad4* or *Tcf7* mRNA levels in femur tissues from C57BL/6 mice, as detected by RT-PCR.

**
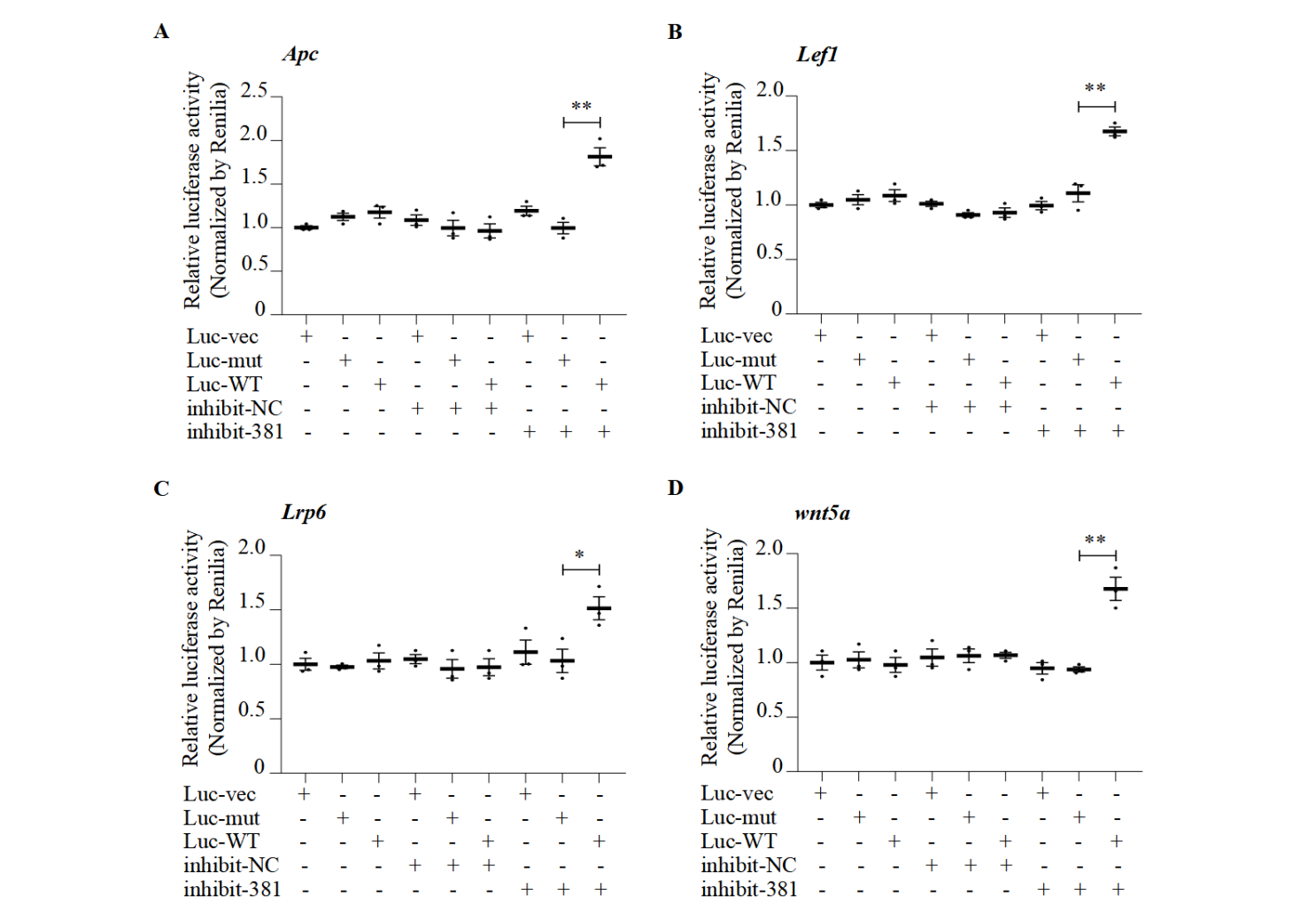
**

**Supplementary Figure 11. miR-381-3p targeted four genes of Wnt signaling pathway**

A-D. Binding effect of miR-381-3p and 3′UTR sequences of *Apc*, *Lef1*, *Lrp6*, and *wnt5a*, as detected by luciferase reporter assay (mean ± S.D., n = 3). Luc-vec: empty luciferase reporter plasmid. Luc-mut: luciferase reporter plasmid containing mutant 3′UTR. Luc-WT: luciferase reporter plasmid containing wild-type 3′UTR. inhibit-NC: inhibitor-NC. inhibit-381: inhibitor-381-3p.

E-F. Quantification results corresponding to Figure4 A-B (mean ± S.D., n > 3).

**P* < 0.05, ***P* < 0.01, ****P* < 0.001

**
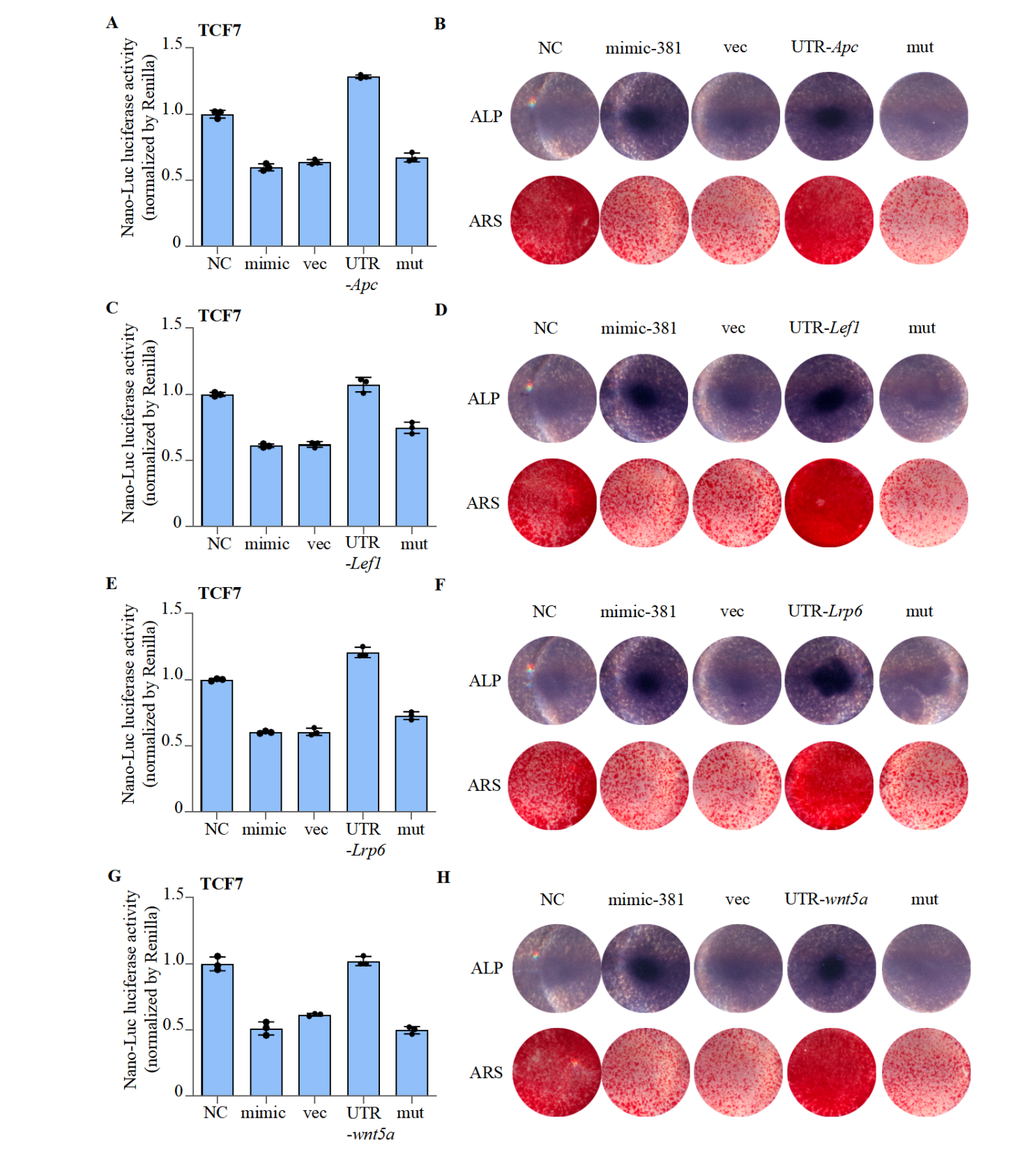
**

**Supplementary Figure 12. miR-381-3p regulated four target genes by combining with their 3′UTR sequence**

A, C, E, G. TCF7 activities of hMSCs after transfected with TCF7 luciferase reporter plasmid and treated with mimic-381-3p and 3′UTR sequences of *Apc*, *Lef1*, *Lrp6*, and *wnt5a*, respectively, as detected by luciferase reporter assay (n = 3). NC: mimic-NC. mimic: mimic-381-3p. vec: cells treated with mimic-381-3p and empty luciferase reporter plasmid. UTR: cells treated with mimic-381-3p and luciferase reporter plasmid containing 3′UTR sequences of *Apc*, *Lef1*, *Lrp6*, and *wnt5a*, respectively. mut: cells treated with mimic-381-3p and luciferase reporter plasmid containing mutant 3′UTR sequences of *Apc*, *Lef1*, *Lrp6*, and *wnt5a*, respectively.

B, D, F, H. ALP and Alizarin Red staining of hMSCs treated with mimic-381-3p and 3′UTR sequences of *Apc*, *Lef1*, *Lrp6*, and *wnt5a*, respectively, as detected by ALP staining and Alizarin Red staining.

**
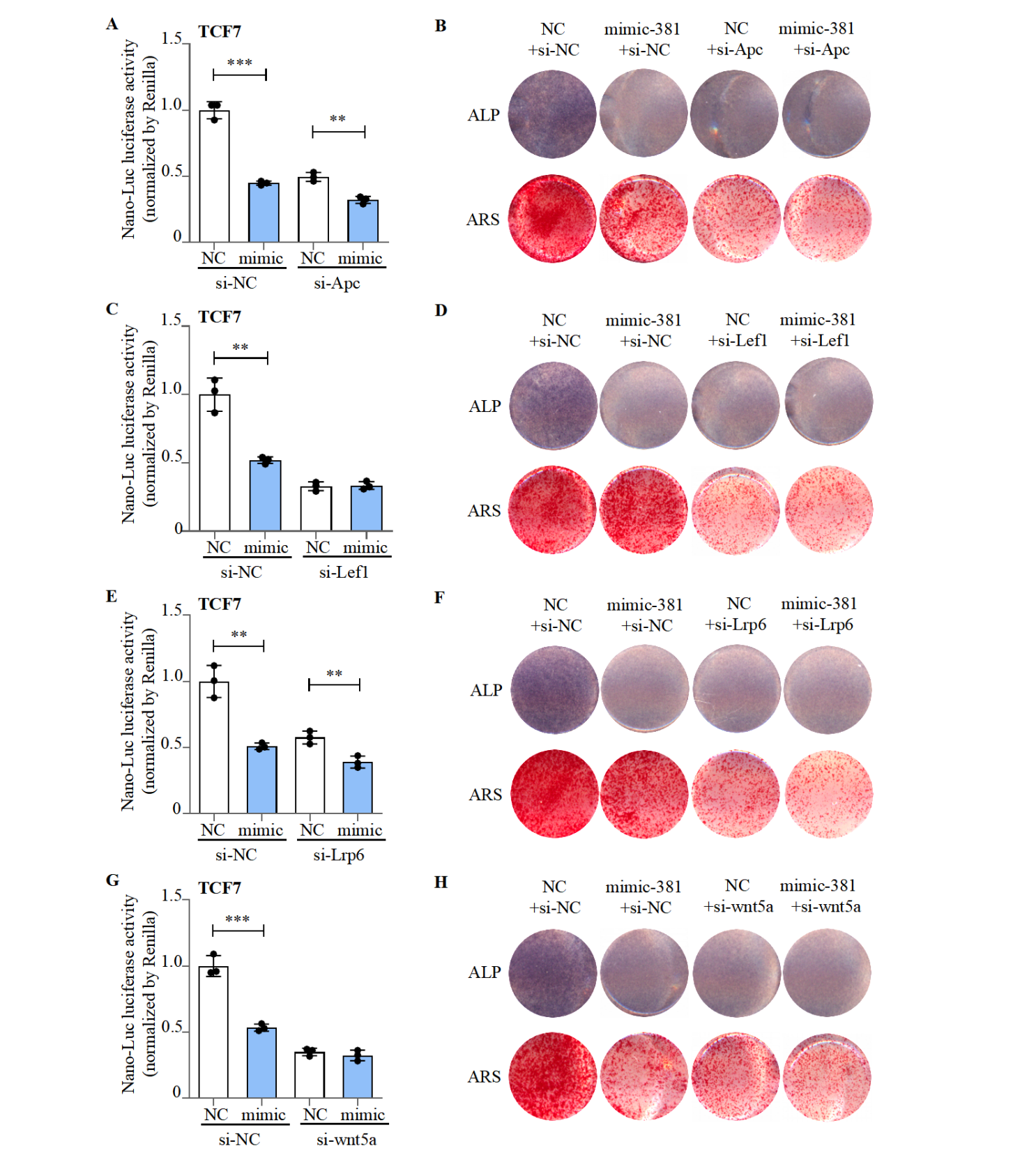
**

**Supplementary Figure 13. Inhibiting the target genes of miR-381-3p can depress the sensitivity of osteogenic differentiation to miR-381-3p**

A, C, E, G. TCF7 activities of hMSCs after transfected with TCF7 luciferase reporter plasmid and treated with mimic-381-3p and siRNA of *Apc*, *Lef1*, *Lrp6*, and *wnt5a*, respectively, as detected by luciferase reporter assay (mean ± S.D., ***P* < 0.01, ****P* < 0.001, n = 3).

B, D, F, H. ALP and Alizarin Red staining of hMSCs treated with mimic-381-3p and siRNA of *Apc*, *Lef1*, *Lrp6*, and *wnt5a*, respectively, as detected by ALP staining and Alizarin Red staining.

**
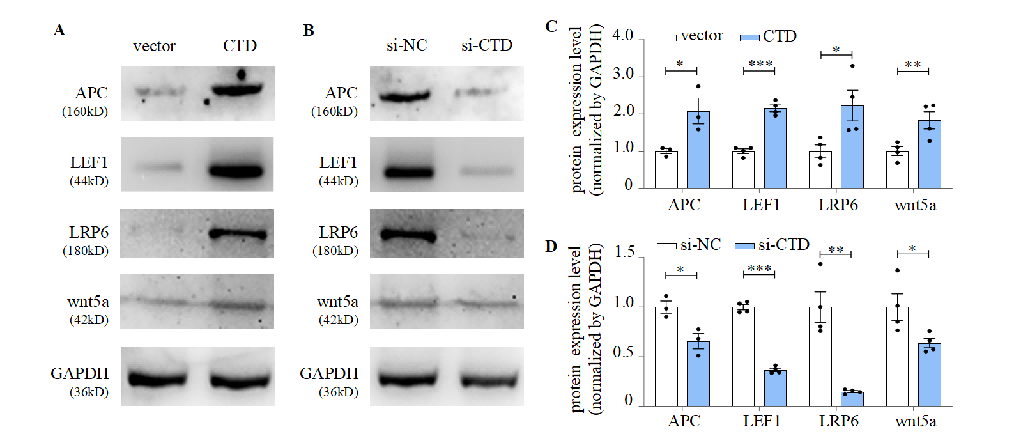
**

**Supplementary Figure 14. CTD-2555A7.2 promoted four target genes of Wnt signaling pathway**

A, C. APC, LEF1, LRP6 and wnt5a protein levels of hMSCs treated with CTD-2555A7.2 over-expression plasmid (compared with pCDNA3.1 plasmid), as detected by western blot. The quantification results with GAPDH as internal control (mean ± S.D., **P* < 0.05, ***P* < 0.01, ****P* < 0.001, n > 3).

B, D. APC, LEF1, LRP6 and wnt5a protein levels of hMSCs treated with CTD-2555A7.2 siRNA (mean ± S.D., **P* < 0.05, ***P* < 0.01, ****P* < 0.001, n > 3).

**
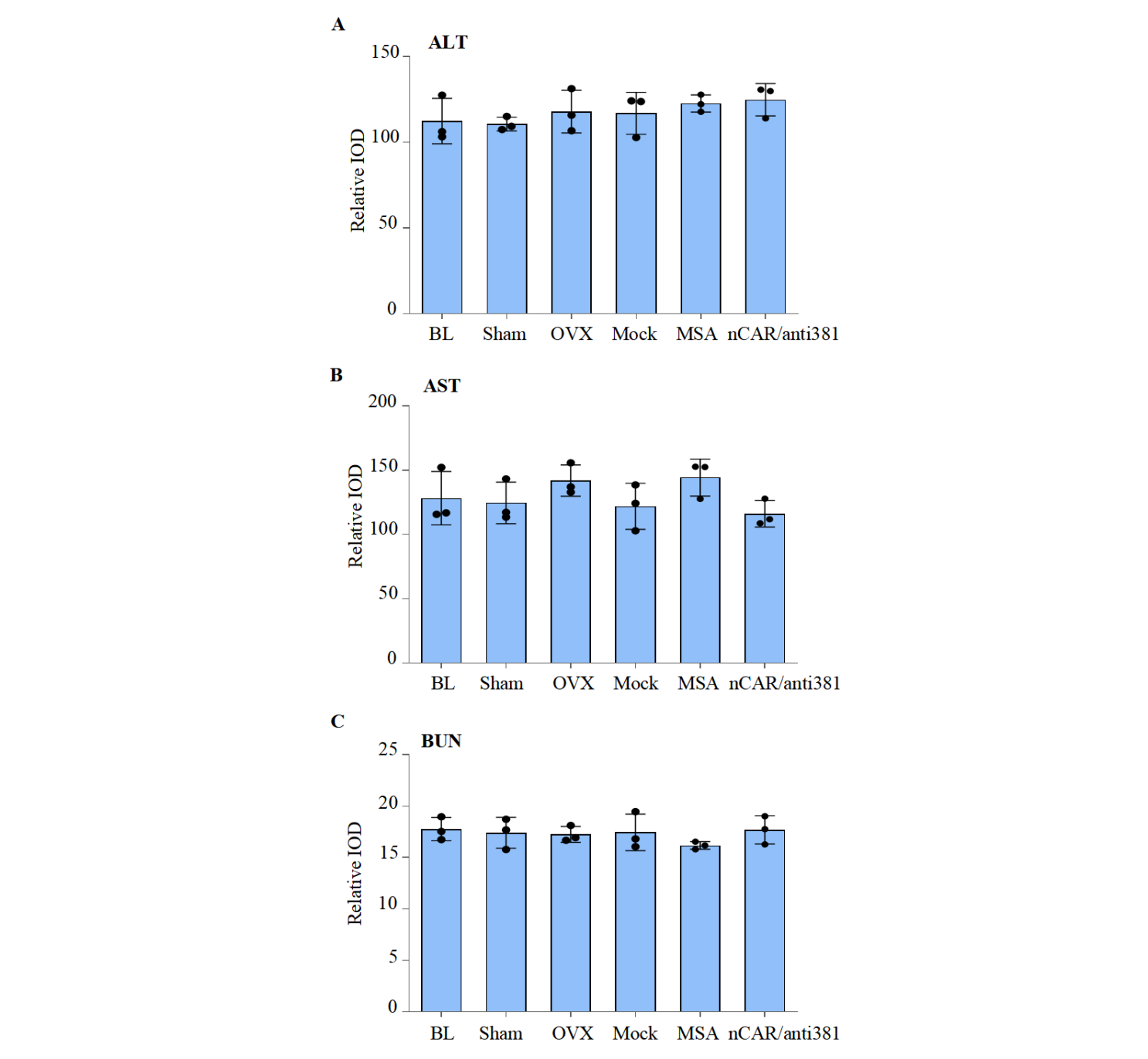
**

**Supplementary Figure 15. Biochemical assays in blood serum of OVX mice after recombinant miR-381-3p inhibitor treatment**

A-C. Alanine transaminase (ALT, A), Aspertate Aminotransferase (AST, B), and Blood Urea Nitrogen (BUN, C) levels in blood serum of OVX mice after recombinant miR-381-3p inhibitor treatment, as detected by ELISA (n = 3). BL (Baseline): sacrifice before RNA treatment. Sham: sham OVX operation group. OVX: OVX group. Mock: transfection reagent control group. MSA: empty recombinant tRNA treated group. nCAR/anti381: novel recombinant miR-381-3p inhibitor treated group.

**
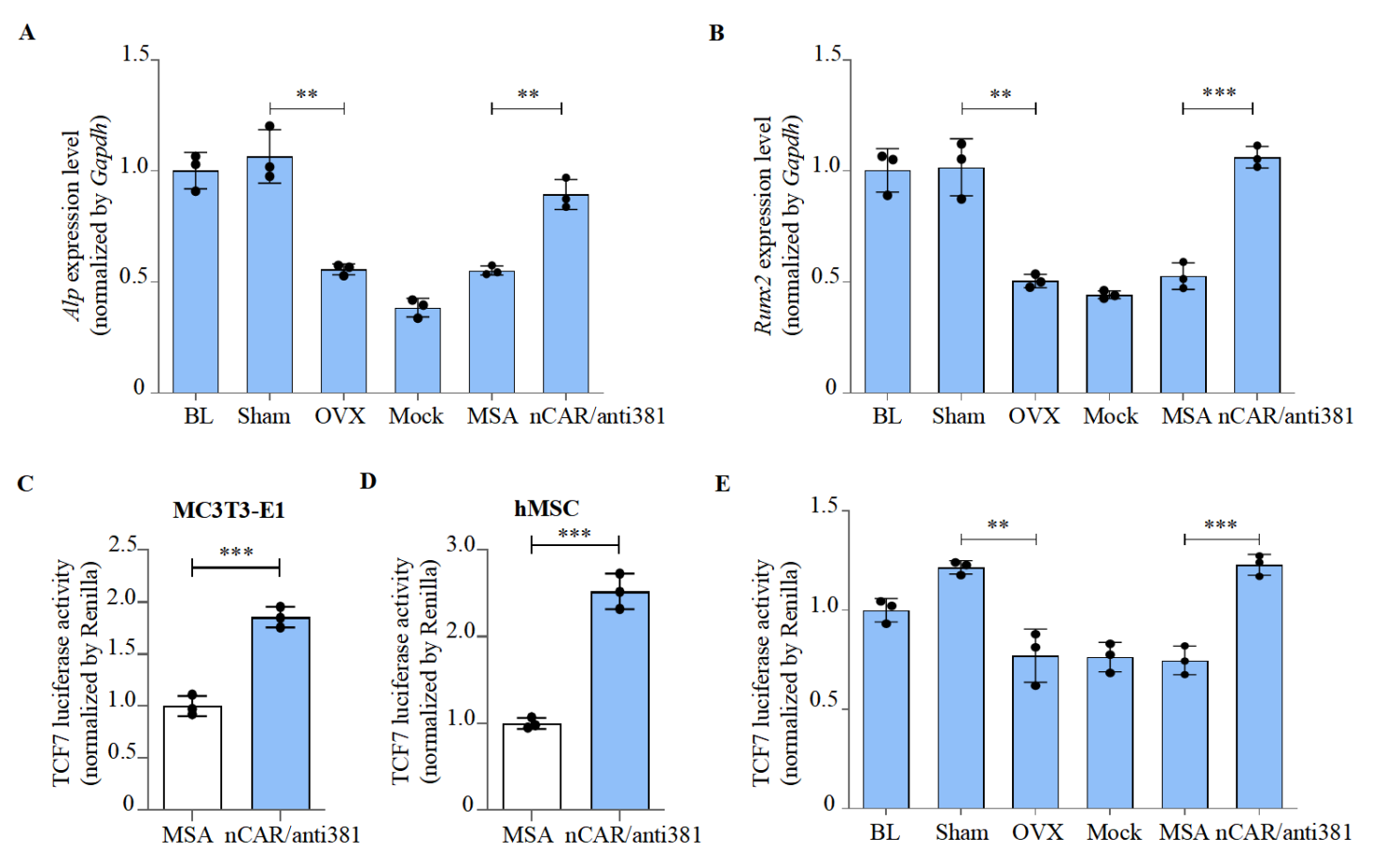
**

**Supplementary Figure 16. Bioengineered recombinant miR-381-3p inhibitor enhanced osteogenic differentiation via Wnt signaling pathway**

A-B. *Alp* or *Runx2* expression levels of primary BMSCs of OVX mice treated with recombinant miR-381-3p inhibitor, as detected by RT-PCR (mean ± S.D., ***P* < 0.01 , ****P* < 0.001, n = 3).

C-D. TCF7 activities of MC3T3-E1 (left) and hMSCs (right) cells treated with recombinant miR-381-3p inhibitor, as detected by luciferase reporter assay (mean ± S.D., ****P* < 0.001, n = 3).

E. TCF7 activities of primary BMSCs of OVX mice treated with recombinant miR-381-3p inhibitor, as detected by luciferase reporter assay (mean ± S.D., ***P* < 0.01, ****P* < 0.001, n = 3).

**
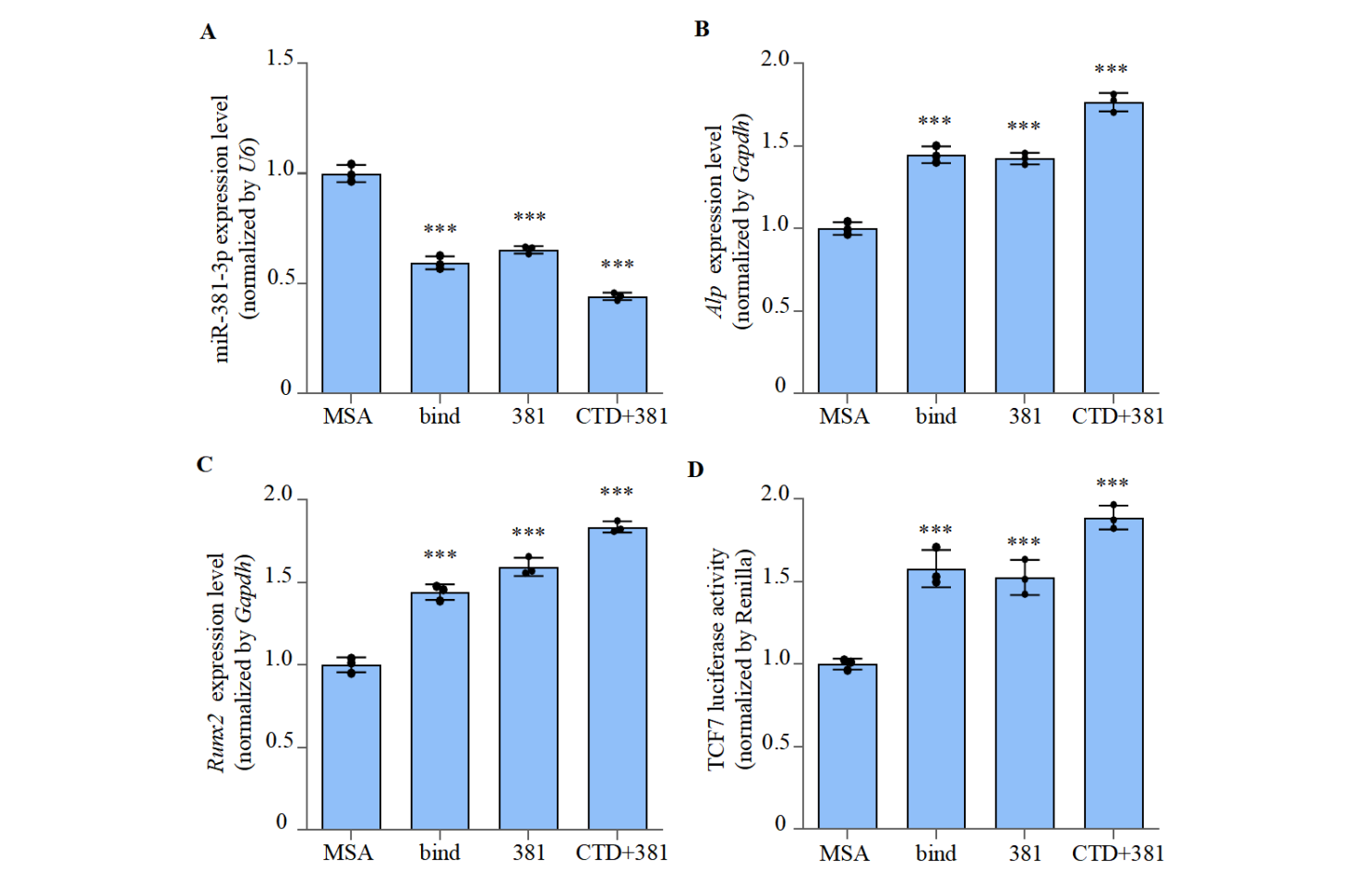
**

**Supplementary Figure 17. Combination effect of CTD-2555A7.2 binding sequence and recombinant miR-381-3p inhibitor on hMSCs**

A-C. miR-381-3p, *Alp* and *Runx2* expression levels of hMSCs treated with CTD-2555A7.2 binding sequence and recombinant miR-381-3p inhibitor, as detected by RT-PCR (mean ± SD, ****P* < 0.001, n = 3). MSA: empty recombinant tRNA as a negative control RNA sequence. bind: CTD-2555A7.2 binding sequence. 381: novel recombinant miR-381-3p inhibitor. CTD+381: Combination of CTD-2555A7.2 binding sequence and recombinant miR-381-3p inhibitor.

D. TCF7 activities of hMSCs treated with CTD-2555A7.2 binding sequence and recombinant miR-381-3p inhibitor, as detected by luciferase reporter assay (mean ± S.D., ****P* < 0.001, n = 3).

**
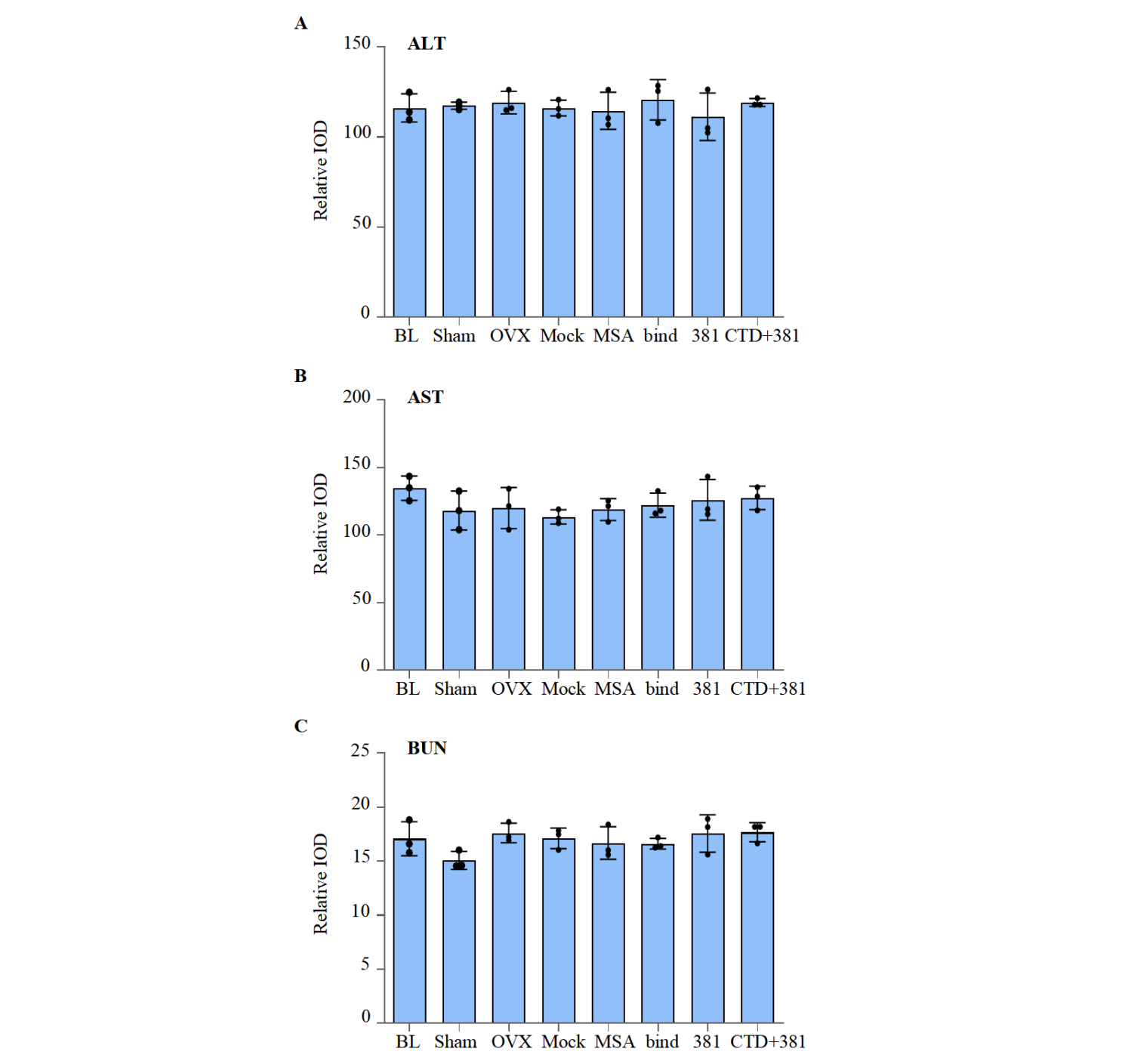
**

**Supplementary Figure 18. Biochemical assays in blood serum of OVX mice after CTD-2555A7.2 binding sequence and recombinant miR-381-3p inhibitor treatment**

1. C. Alanine transaminase (ALT, A), Aspertate Aminotransferase (AST, B), and Blood Urea Nitrogen (BUN, C) levels in blood serum of OVX mice after CTD-2555A7.2 binding sequence and recombinant miR-381-3p inhibitor treatment, as detected by ELISA (n = 3). BL (Baseline): sacrifice before RNA treatment. Sham: sham OVX operation group. OVX: OVX group. Mock: transfection reagent control group. MSA: empty recombinant tRNA treated group. bind: CTD-2555A7.2 binding sequence treated group. 381: novel recombinant miR-381-3p inhibitor treated group. CTD+381: CTD-2555A7.2 binding sequence and recombinant miR-381-3p inhibitor combined treated group.

**
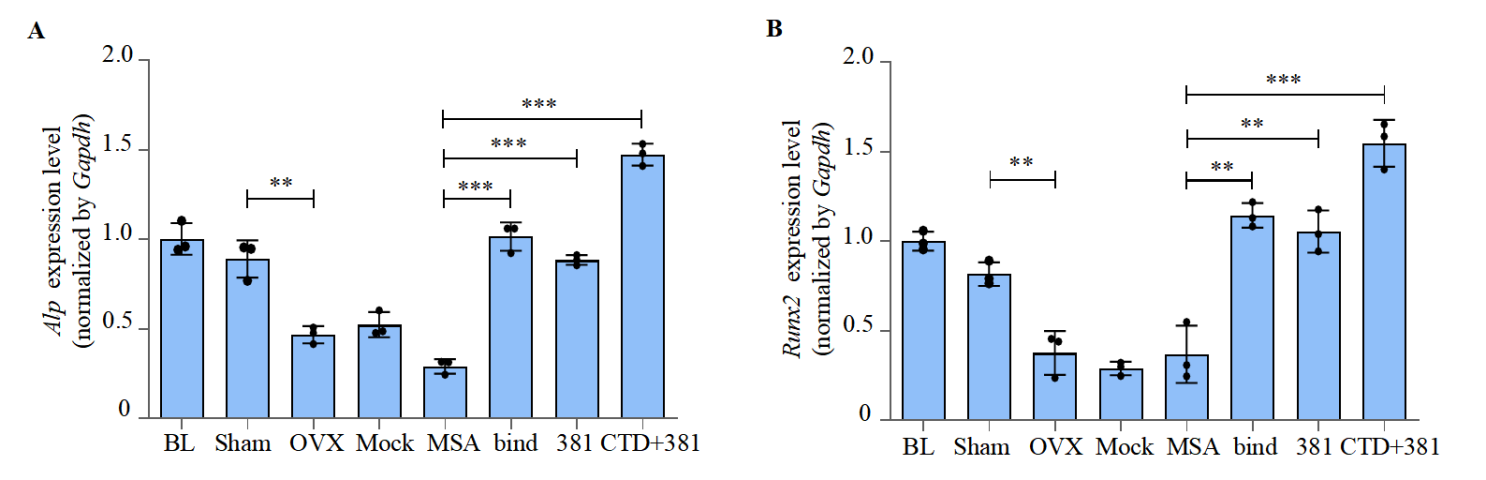
**

**Supplementary Figure 19. Combination of CTD-2555A7.2 binding sequence and bioengineered recombinant miR-381-3p inhibitor enhanced osteogenic differentiation of OVX mice**

A-B. Expression levels of *Alp* and *Runx2* in primary BMSCs of OVX mice treated with CTD-2555A7.2 binding sequence and recombinant miR-381-3p inhibitor, as detected by RT-PCR (mean ± S.D., ***P* < 0.01 , ****P* < 0.001, n = 3).


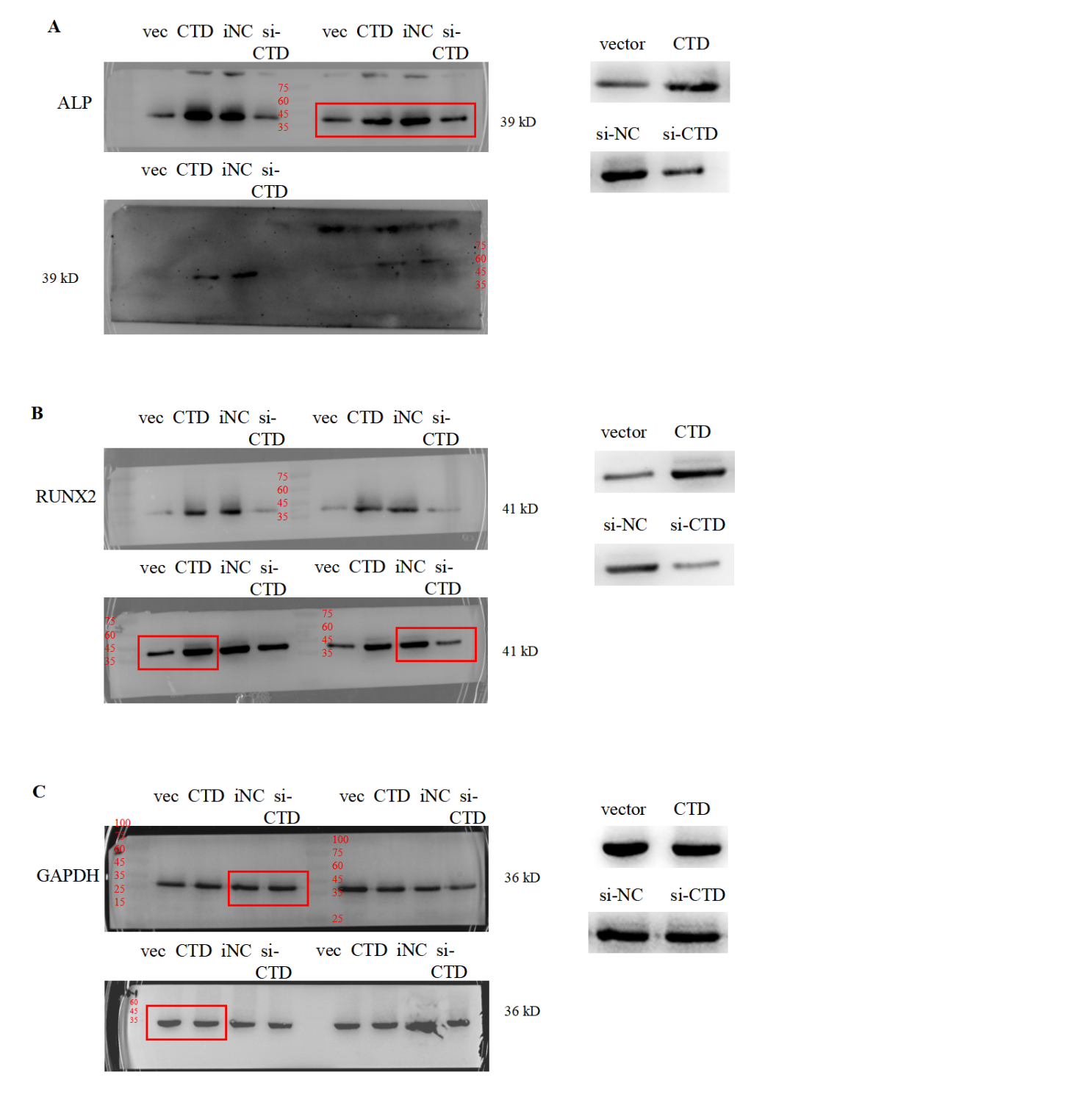


**Supplemental Figure 20. Uncropped images of the original western blots in Figure 1 (H-I)**

**
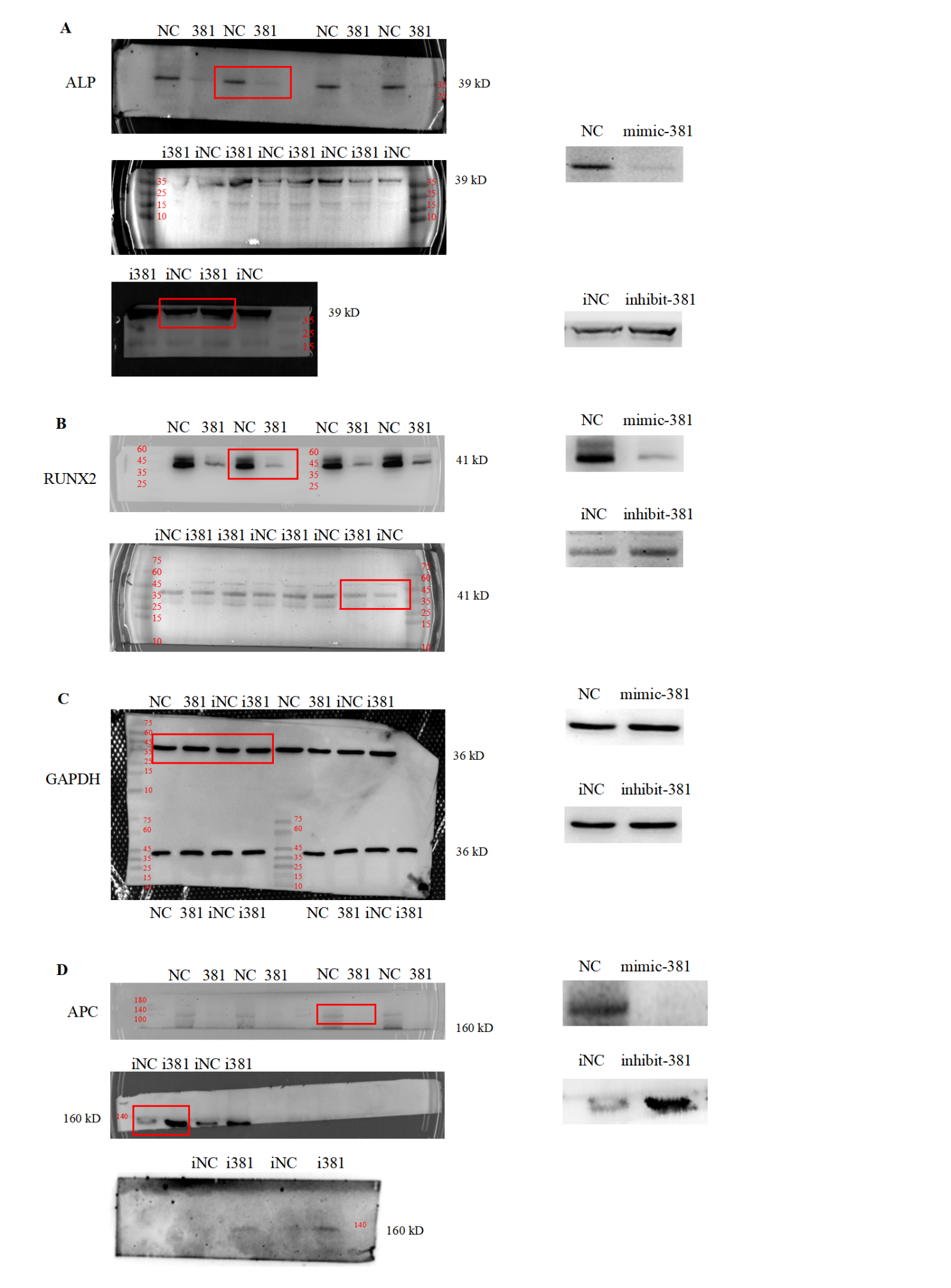
**

**Supplemental Figure 21. Uncropped images of the original western blots in Figure 3 (F, G) and 4 (A, C)**

**
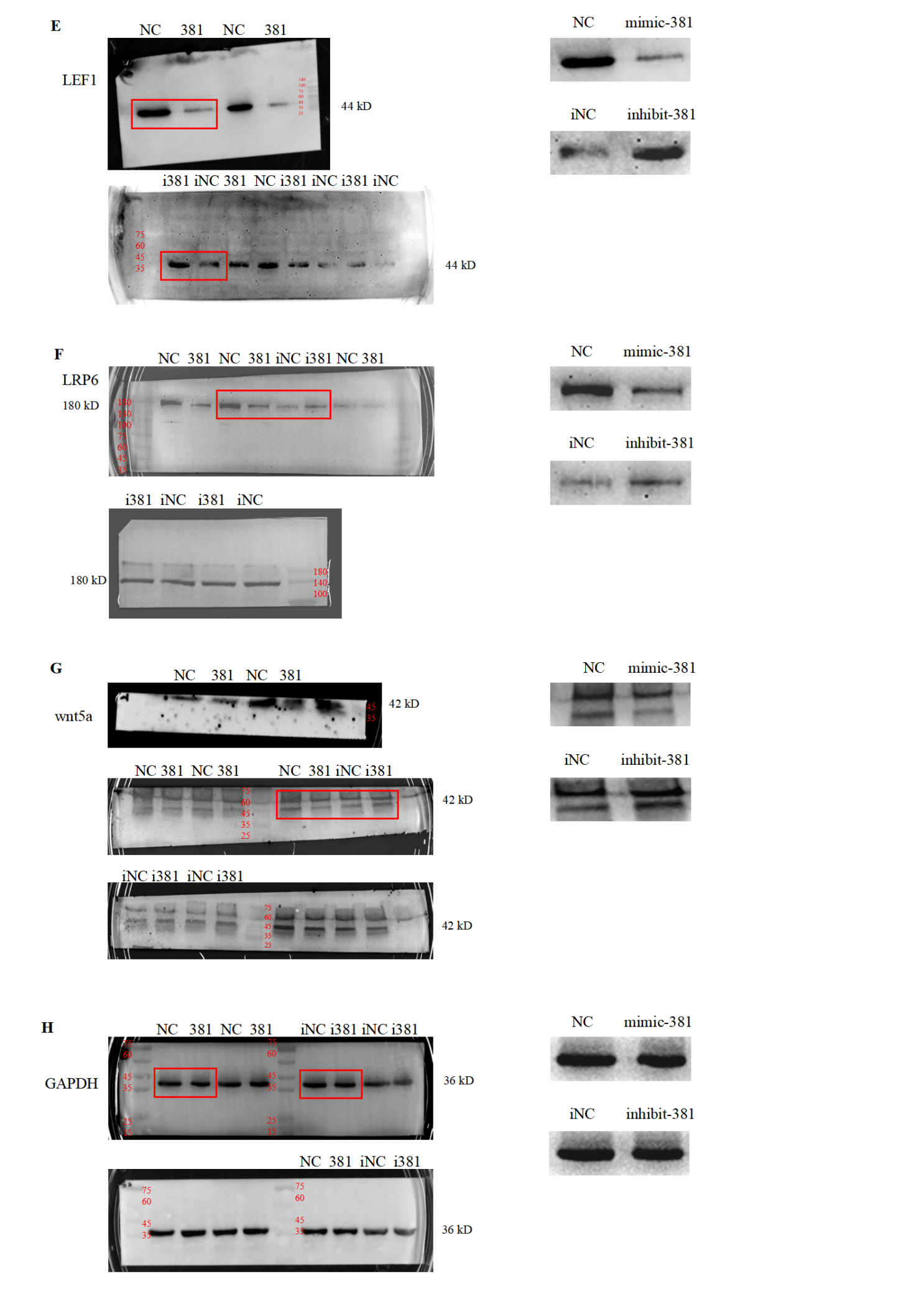
**

**Supplemental Figure 22. Uncropped images of the original western blots in Figure 4 (A, C)**

**
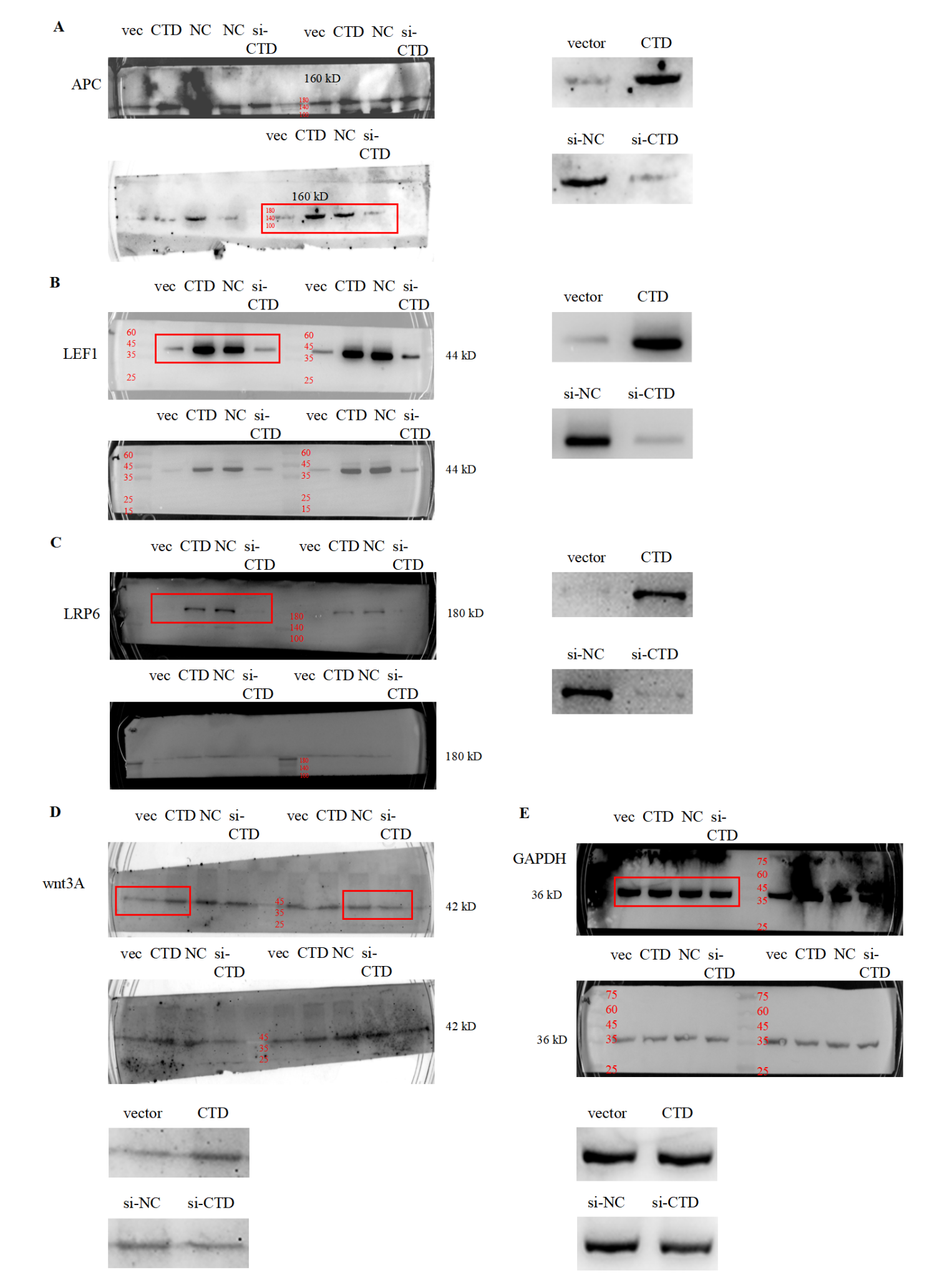
**

**Supplemental Figure 23. Uncropped images of the original western blots in Figure S14 (A, C)**
