# Supplementary figures and images for: LncRNA CTD-2555A7.2 promotes bone formation with LncRNA-specific cascade amplification strategy
Source: Sci Rep. 2025 Jul 1;15:20687. doi: 10.1038/s41598-025-05826-z (PMC12217230; doi:10.1038/s41598-025-05826-z)

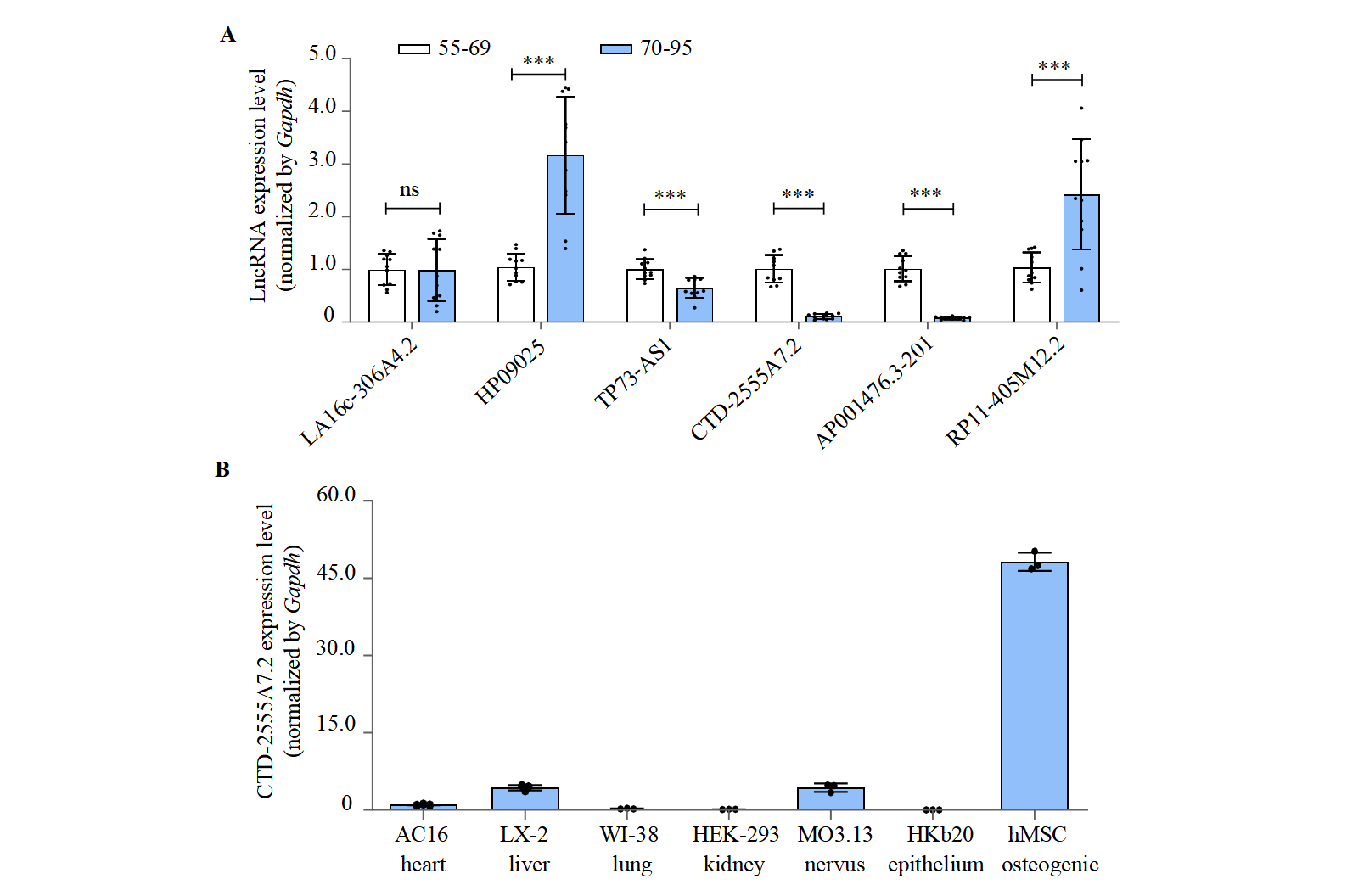

Supplement: Supplementary file 2 — Supplementary Material 2 [file 41598_2025_5826_MOESM2_ESM.tif]

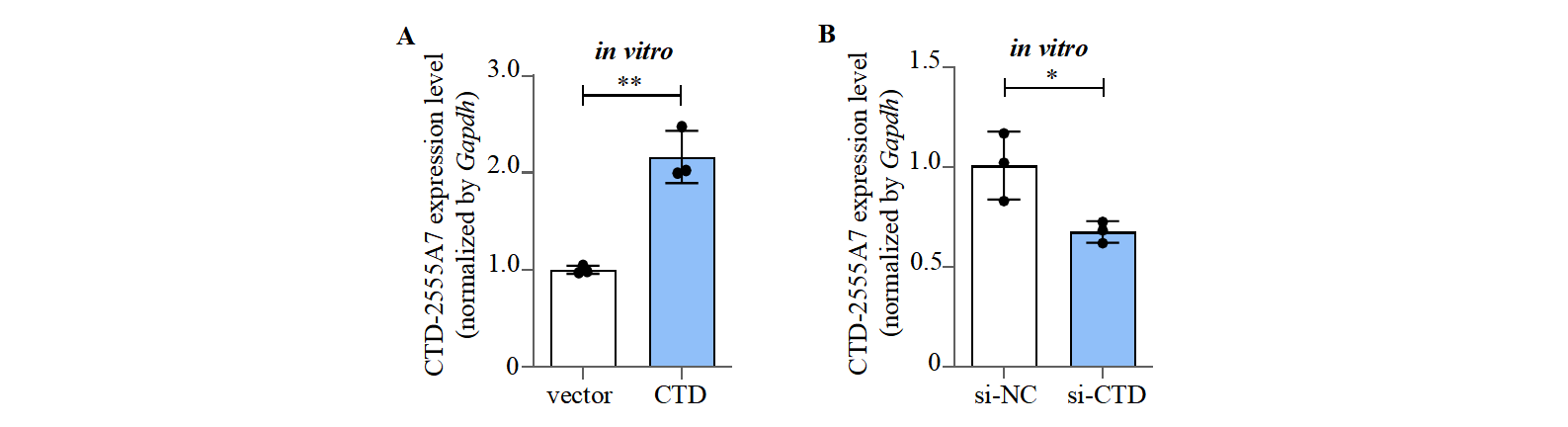

Supplement: Supplementary file 3 — Supplementary Material 3 [file 41598_2025_5826_MOESM3_ESM.tif]

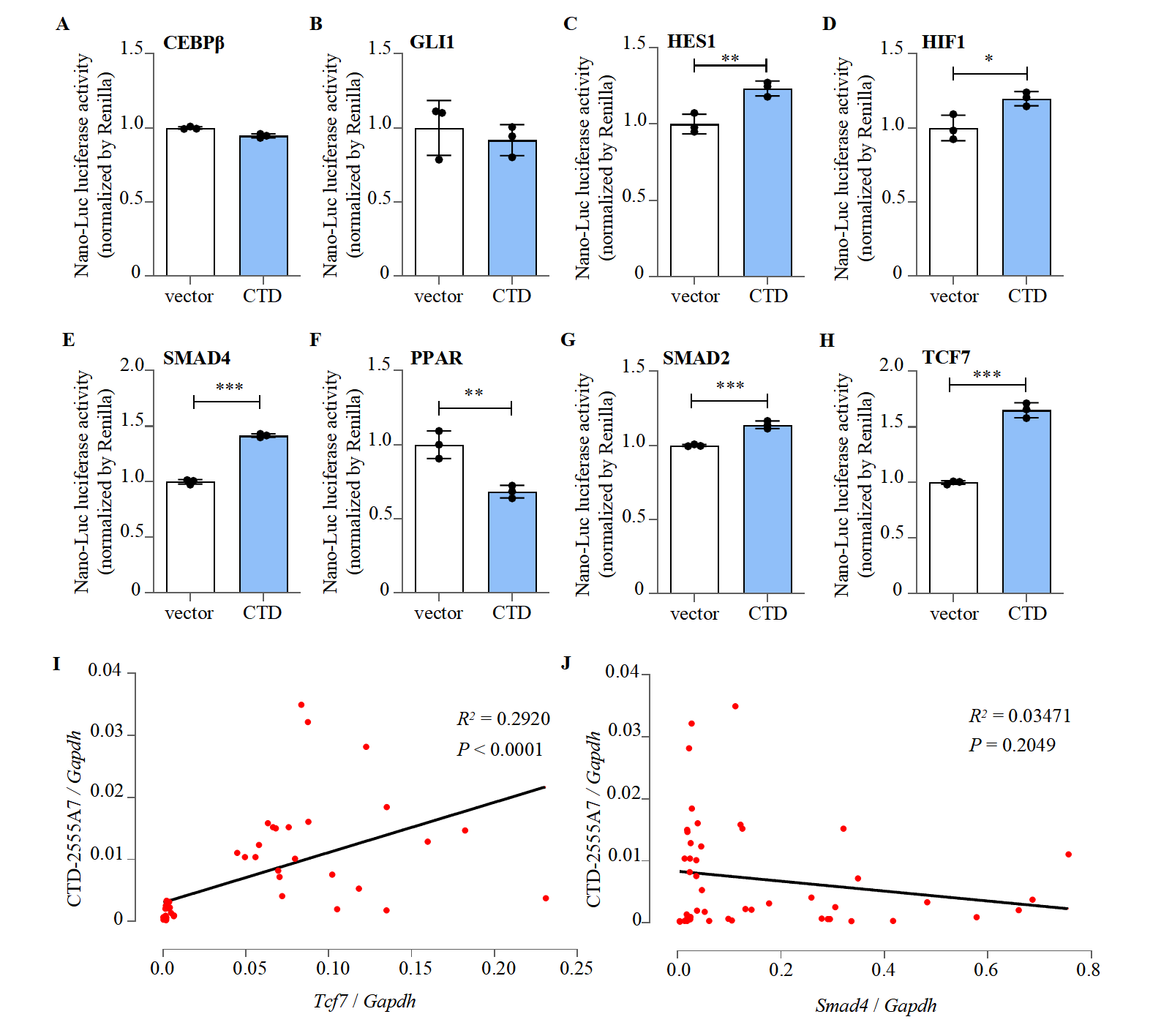

Supplement: Supplementary file 4 — Supplementary Material 4 [file 41598_2025_5826_MOESM4_ESM.tif]

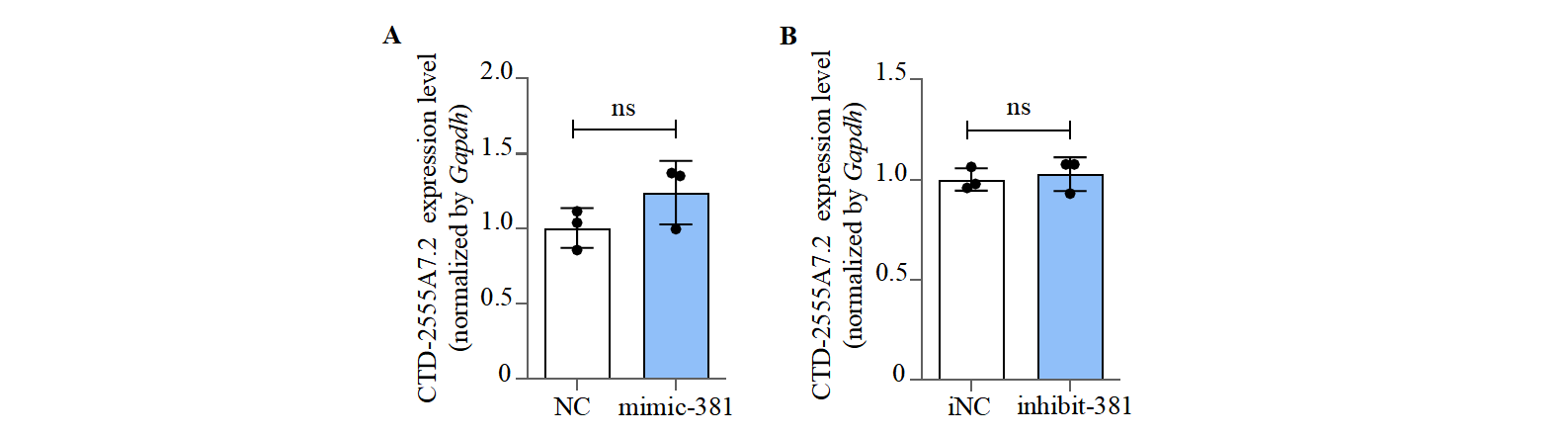

Supplement: Supplementary file 5 — Supplementary Material 5 [file 41598_2025_5826_MOESM5_ESM.tif]

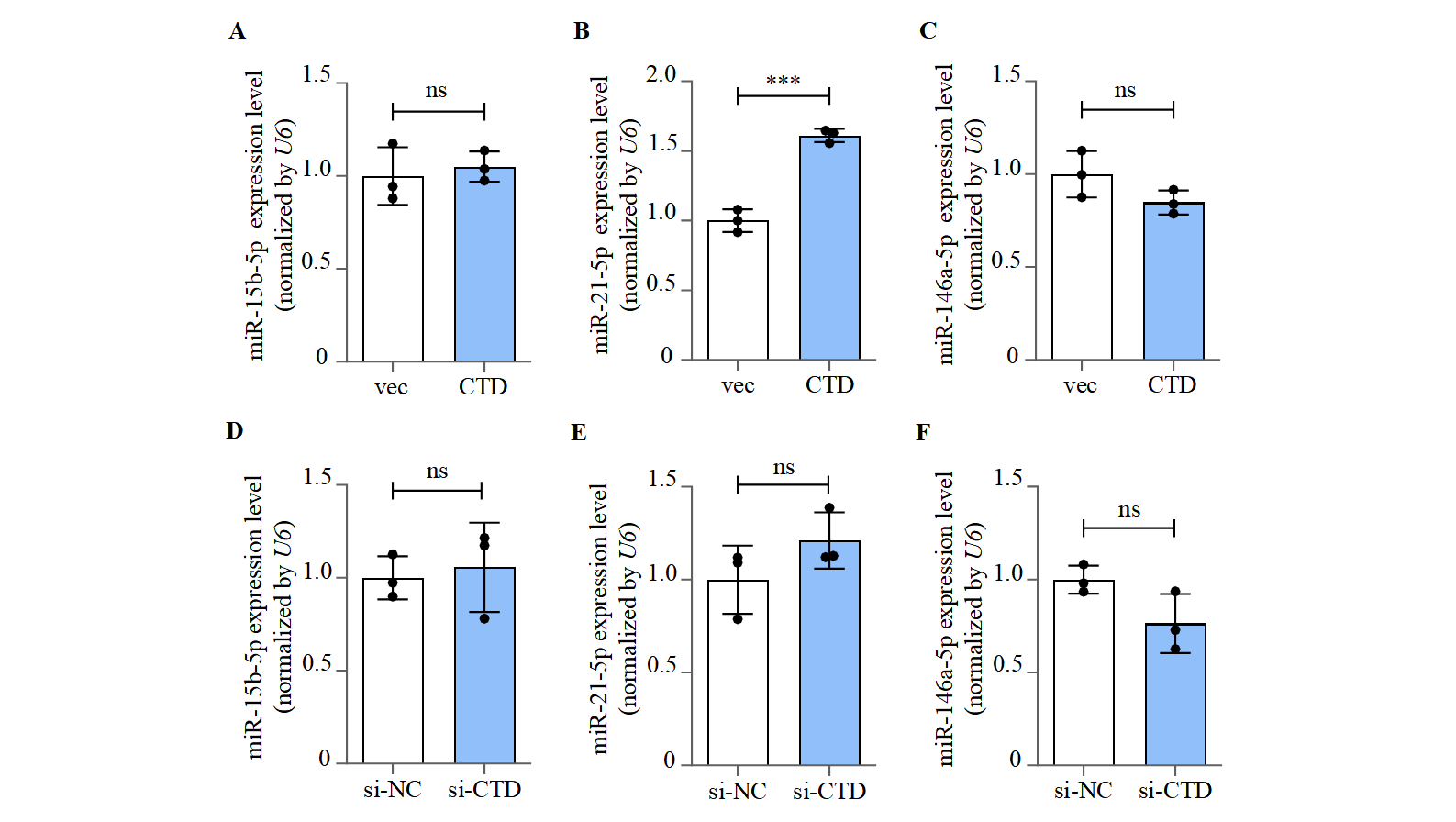

Supplement: Supplementary file 6 — Supplementary Material 6 [file 41598_2025_5826_MOESM6_ESM.tif]

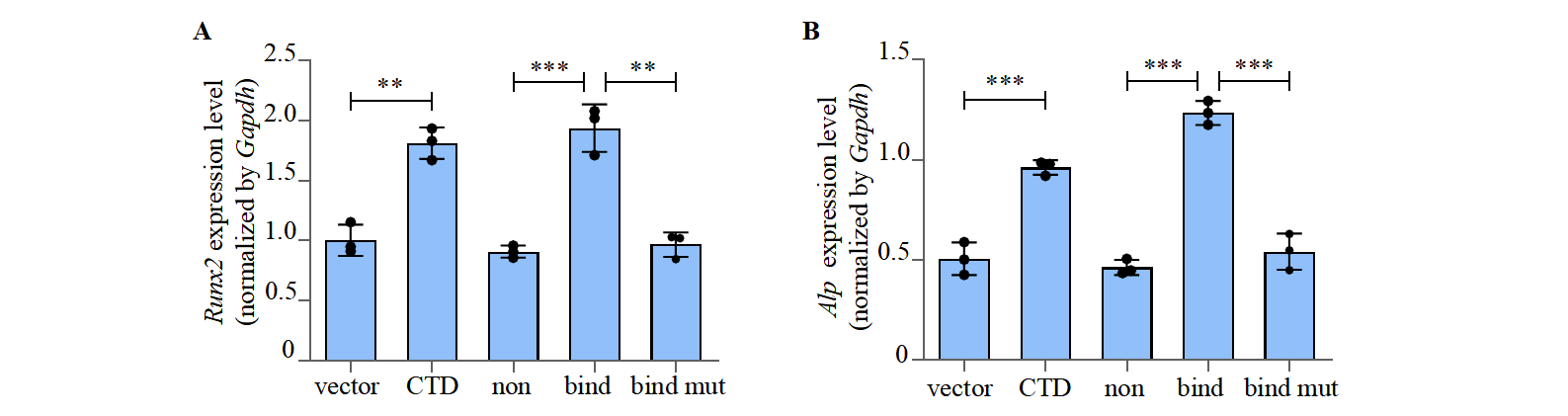

Supplement: Supplementary file 7 — Supplementary Material 7 [file 41598_2025_5826_MOESM7_ESM.tif]

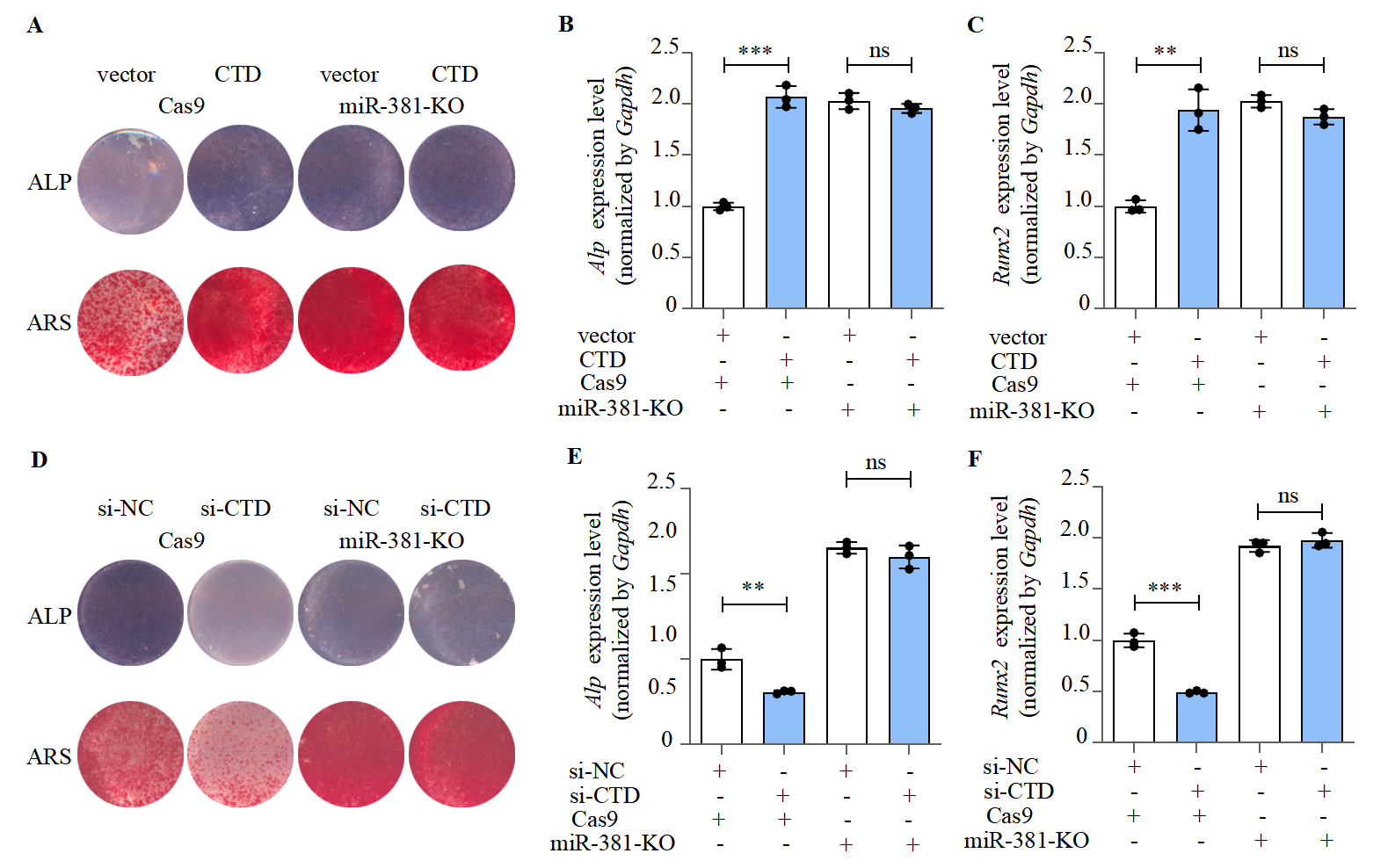

Supplement: Supplementary file 8 — Supplementary Material 8 [file 41598_2025_5826_MOESM8_ESM.tif]

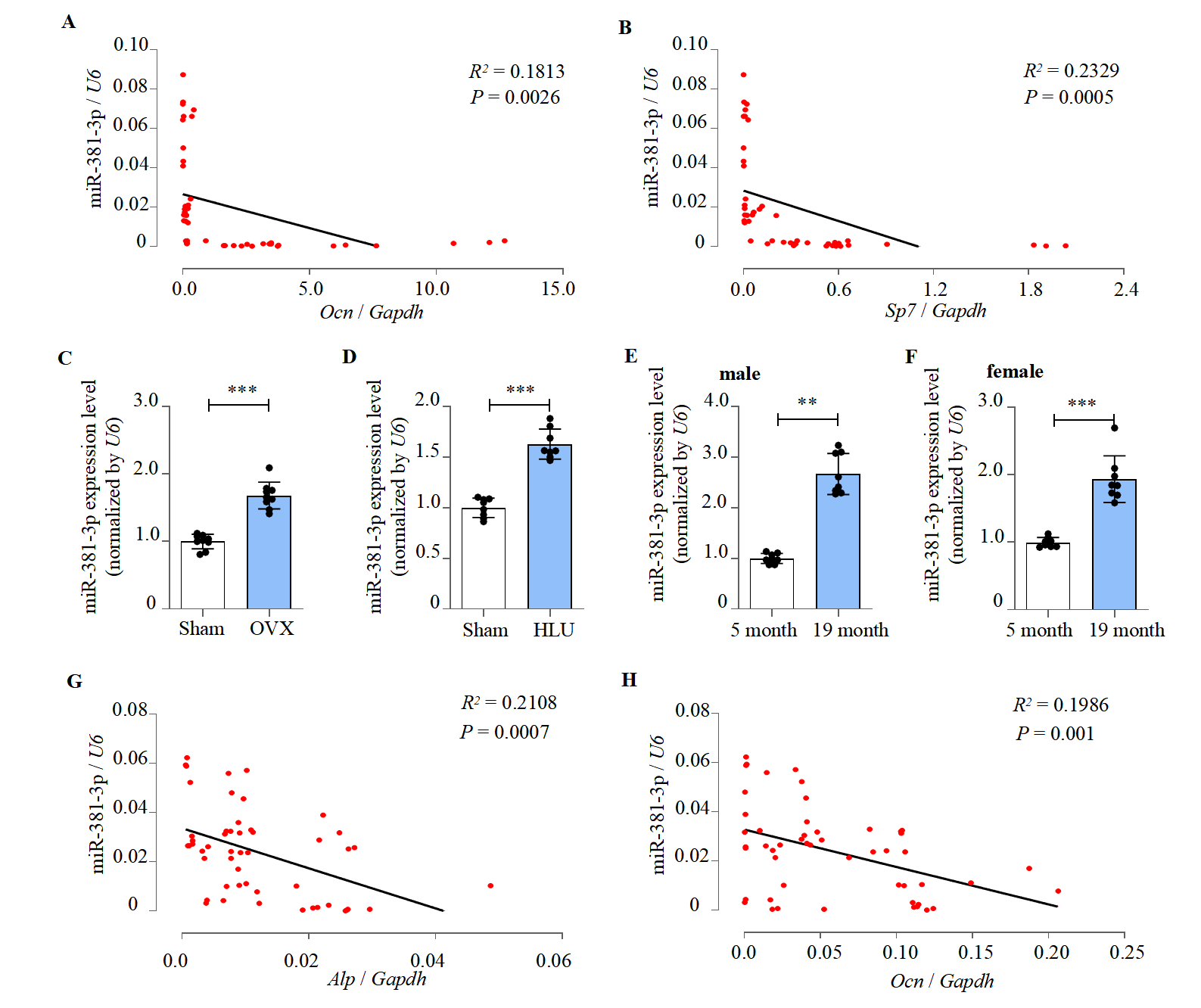

Supplement: Supplementary file 9 — Supplementary Material 9 [file 41598_2025_5826_MOESM9_ESM.tif]

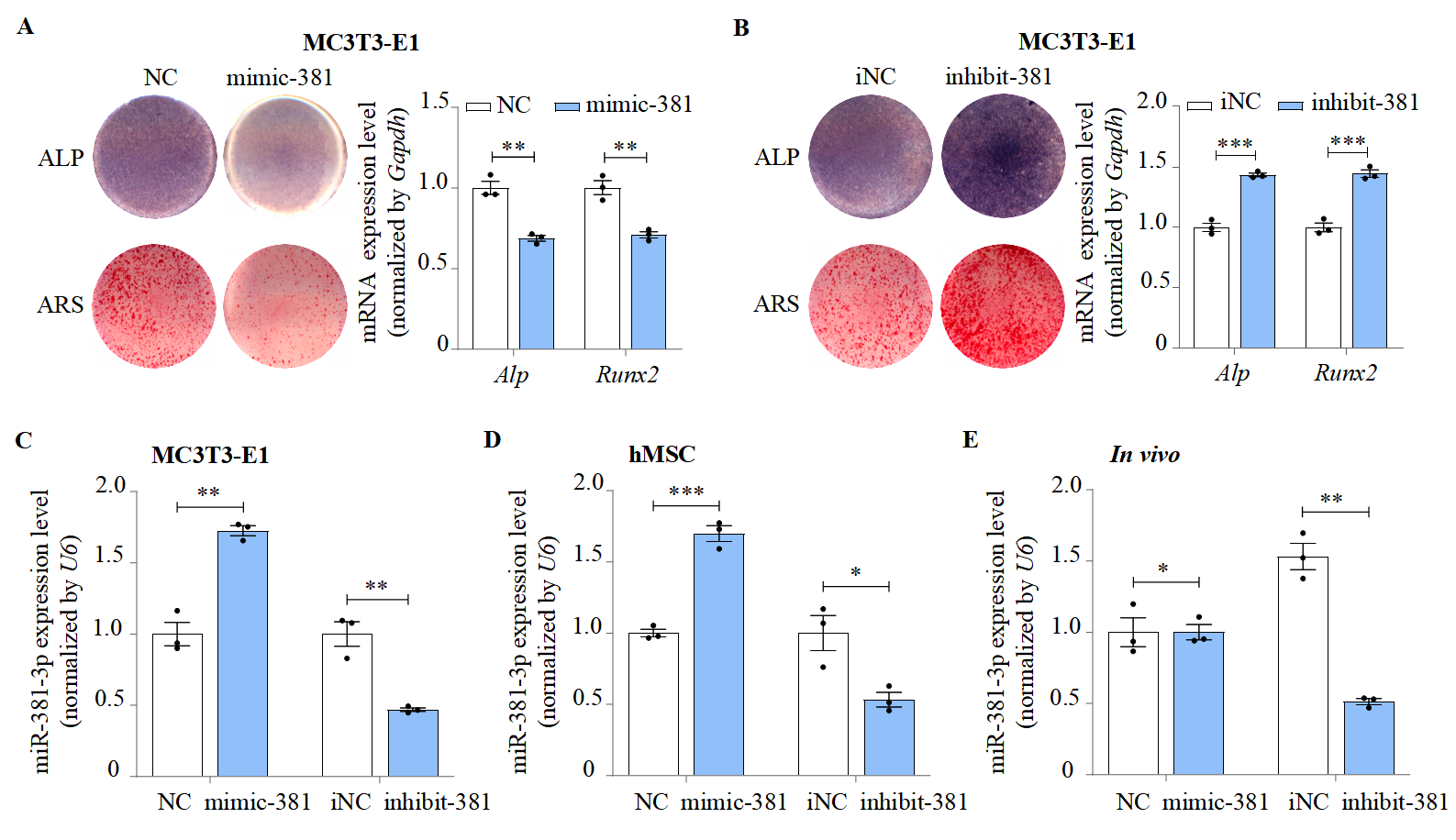

Supplement: Supplementary file 10 — Supplementary Material 10 [file 41598_2025_5826_MOESM10_ESM.tif]

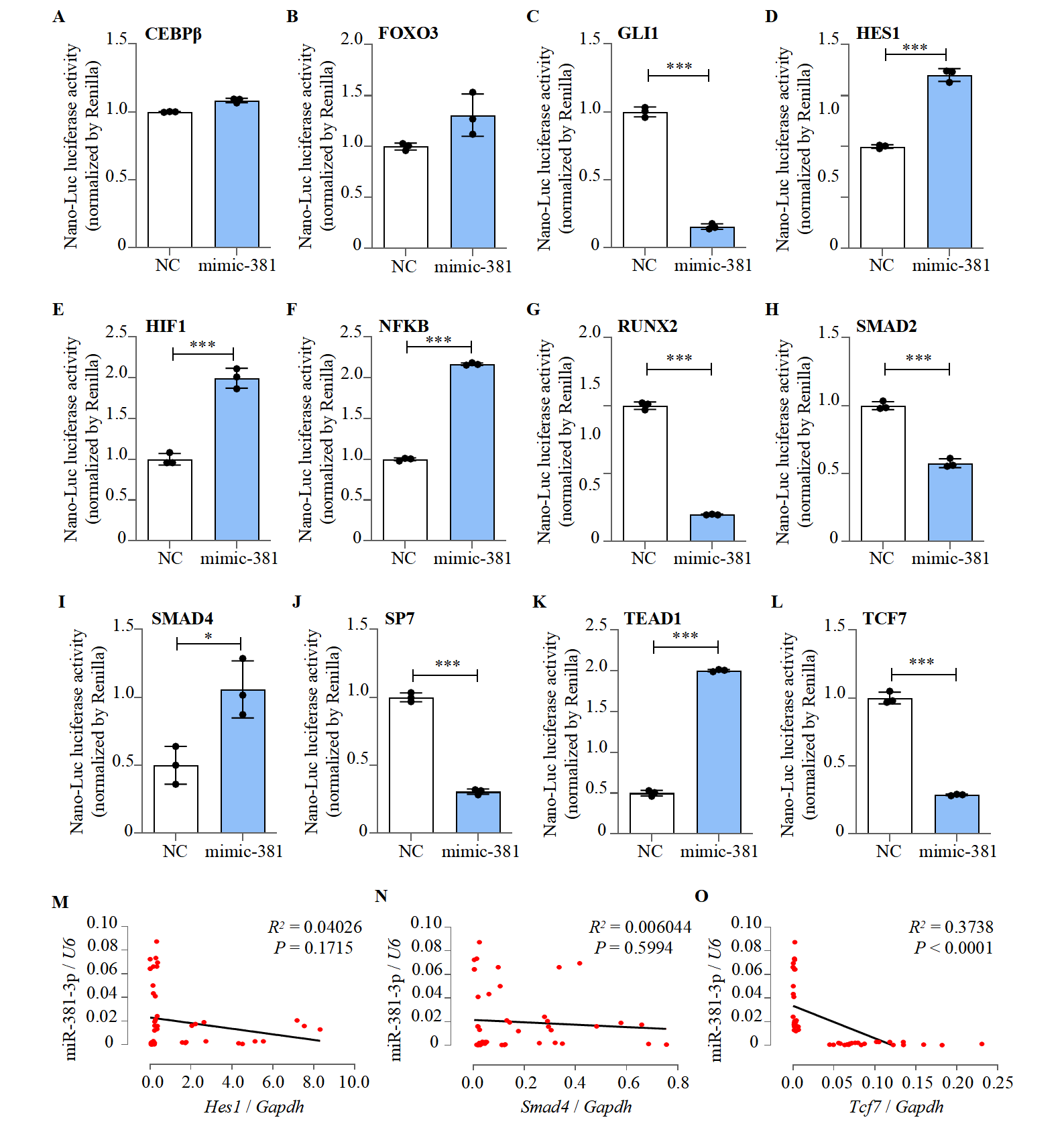

Supplement: Supplementary file 11 — Supplementary Material 11 [file 41598_2025_5826_MOESM11_ESM.tif]

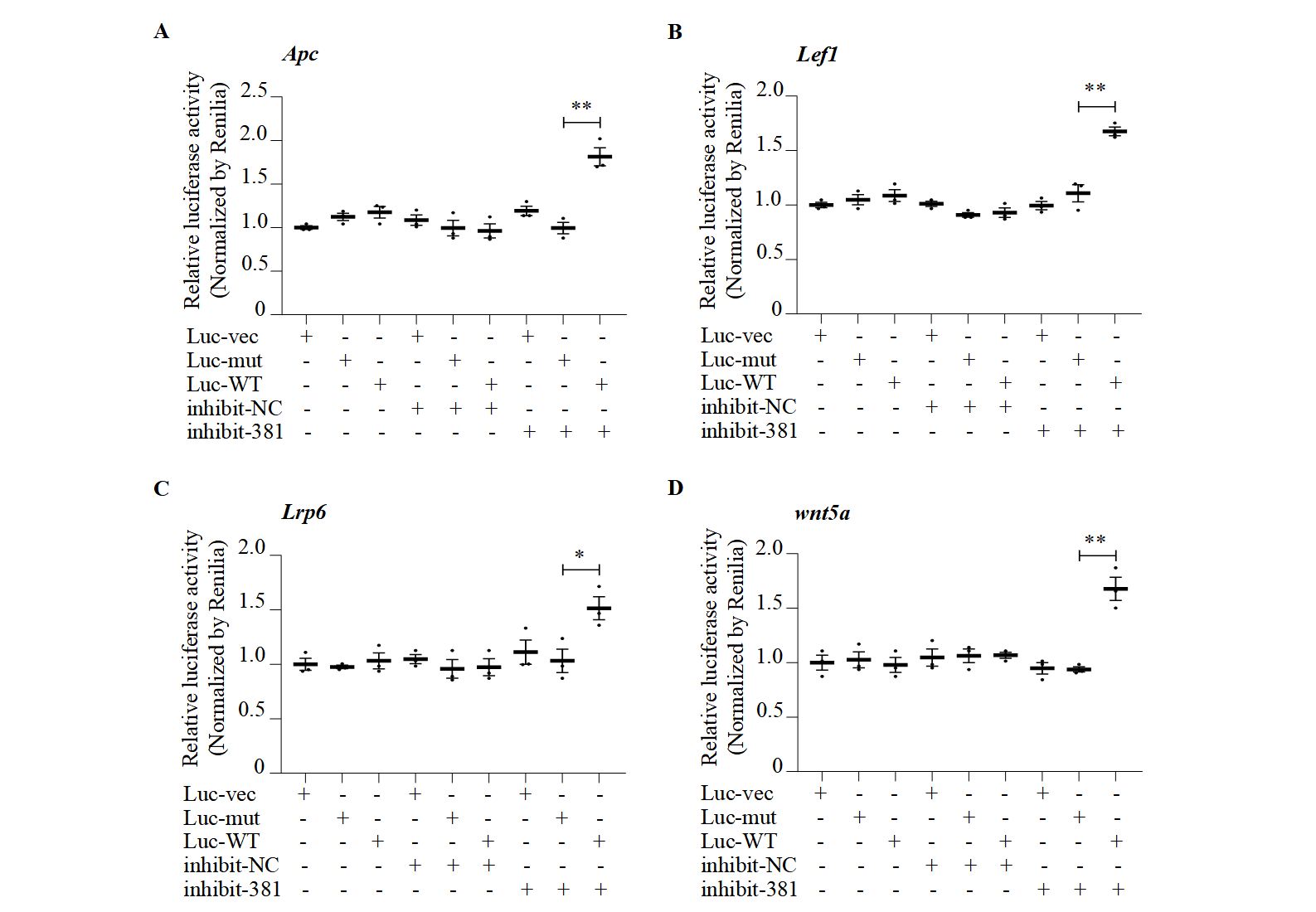

Supplement: Supplementary file 12 — Supplementary Material 12 [file 41598_2025_5826_MOESM12_ESM.tif]

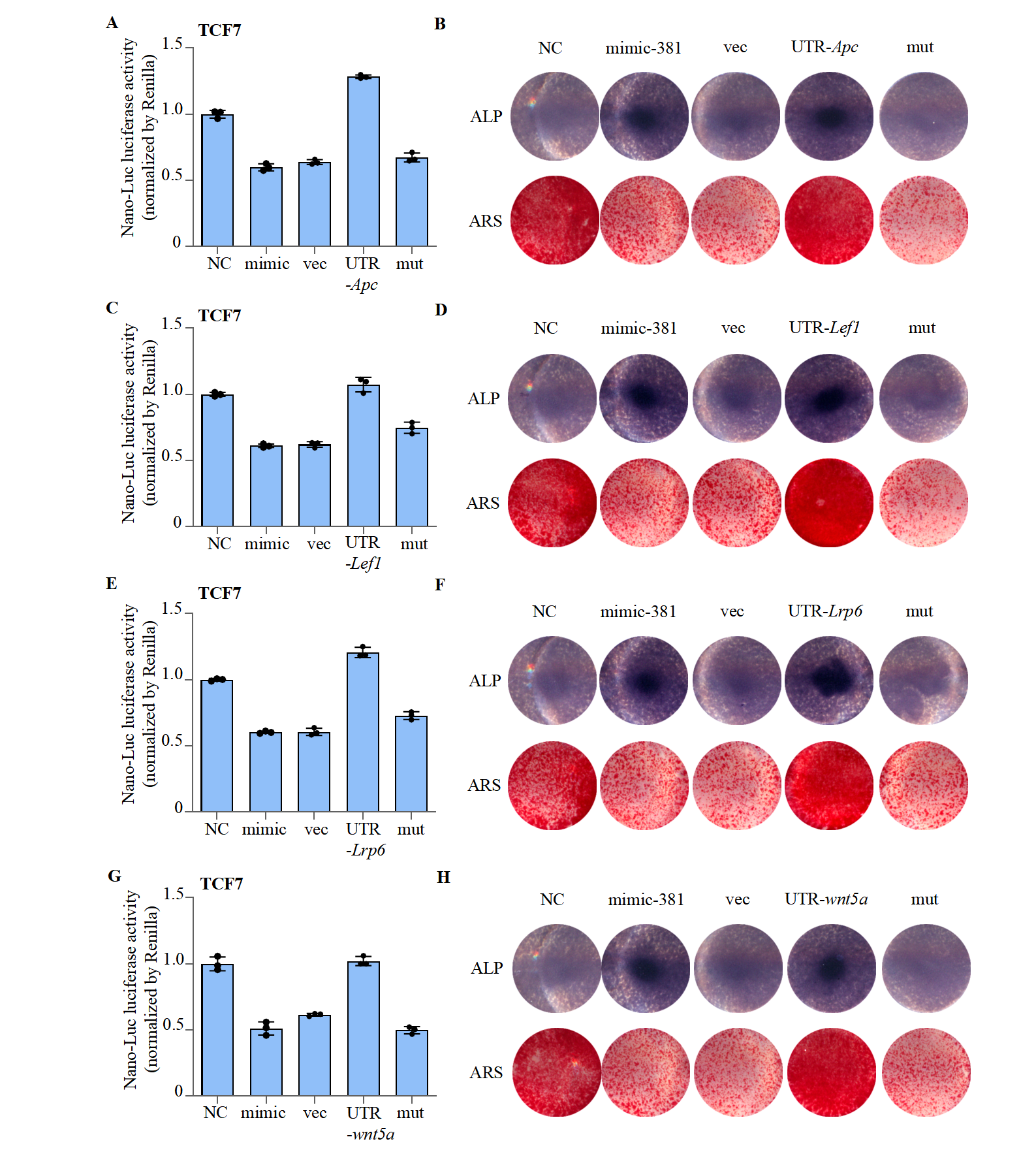

Supplement: Supplementary file 13 — Supplementary Material 13 [file 41598_2025_5826_MOESM13_ESM.tif]

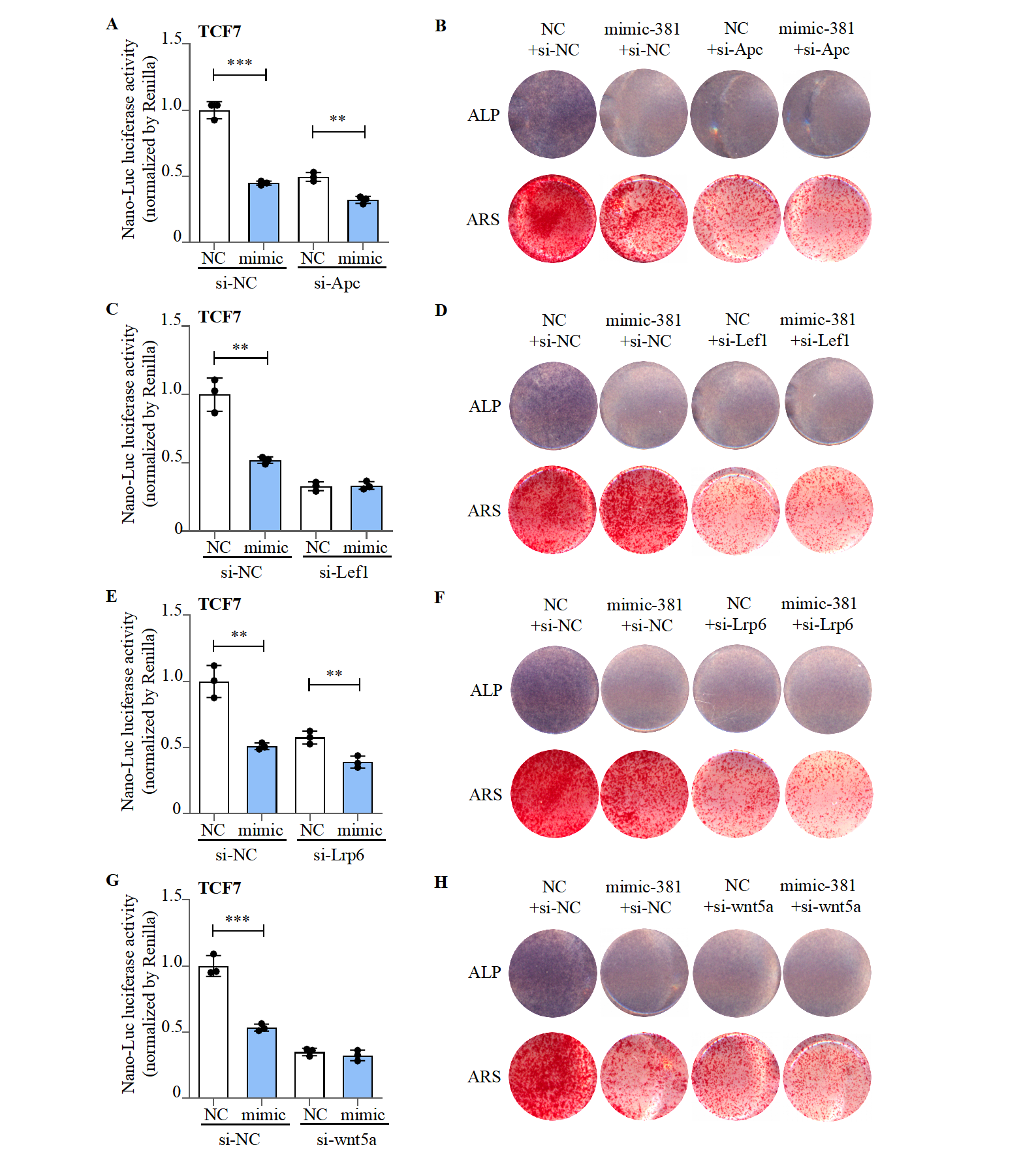

Supplement: Supplementary file 14 — Supplementary Material 14 [file 41598_2025_5826_MOESM14_ESM.tif]

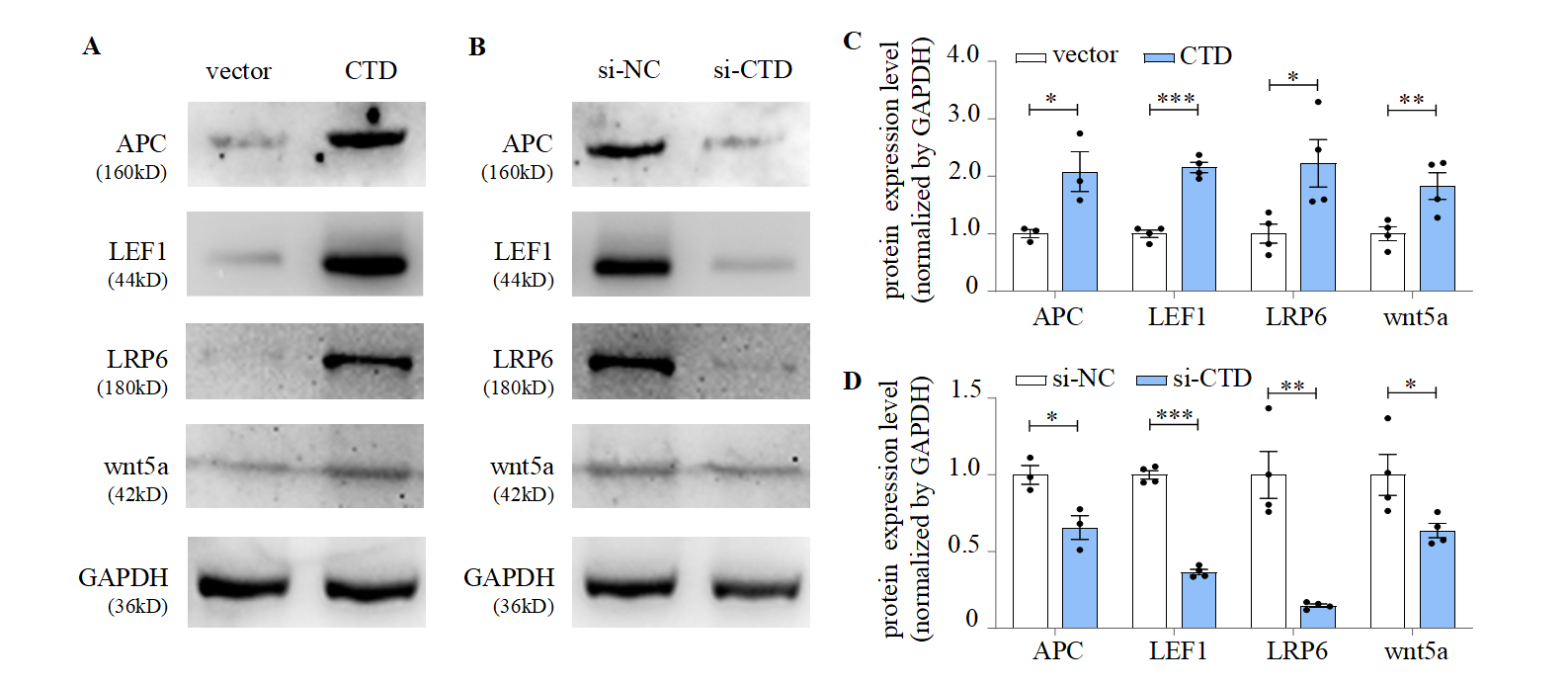

Supplement: Supplementary file 15 — Supplementary Material 15 [file 41598_2025_5826_MOESM15_ESM.tif]

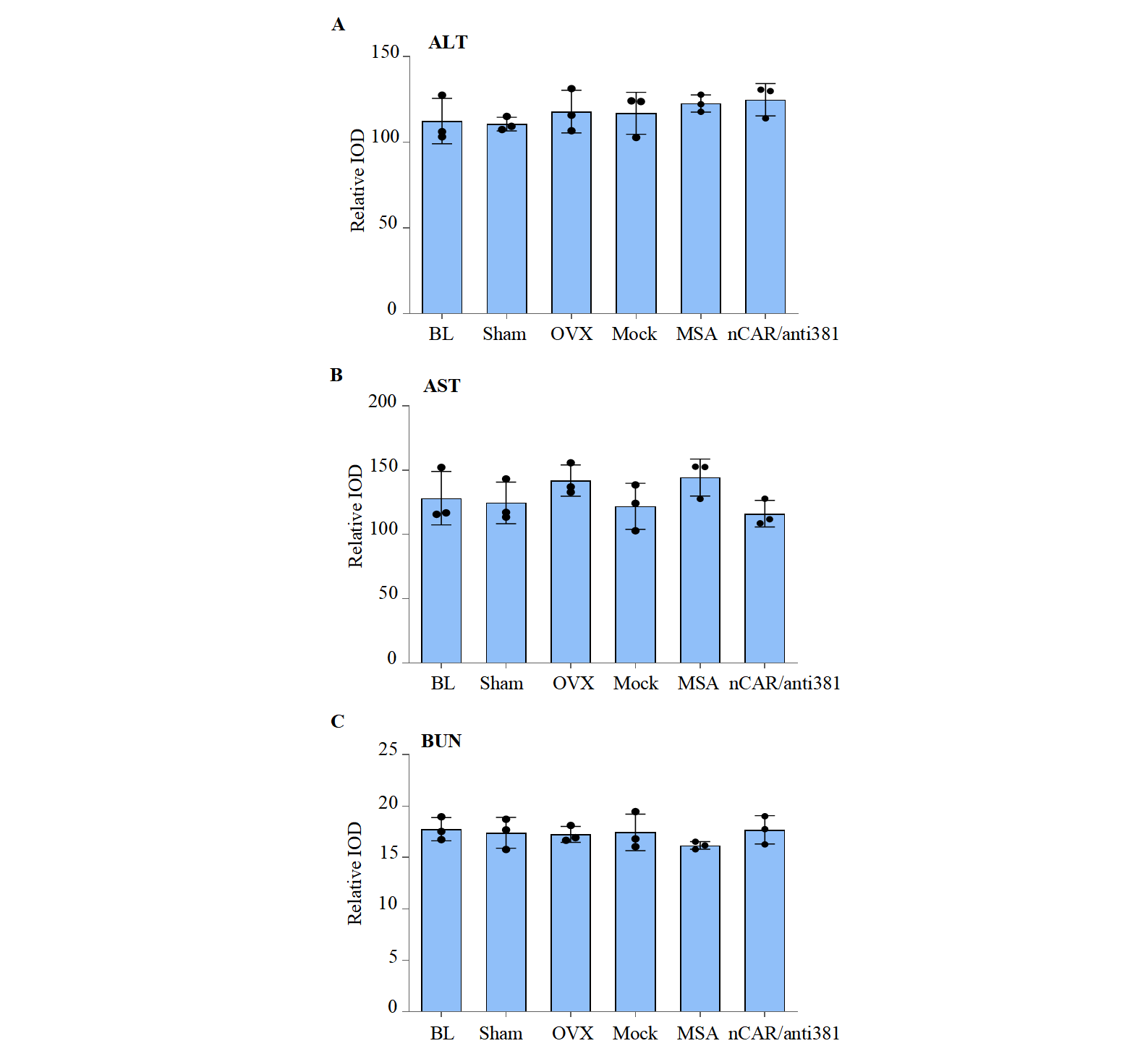

Supplement: Supplementary file 16 — Supplementary Material 16 [file 41598_2025_5826_MOESM16_ESM.tif]

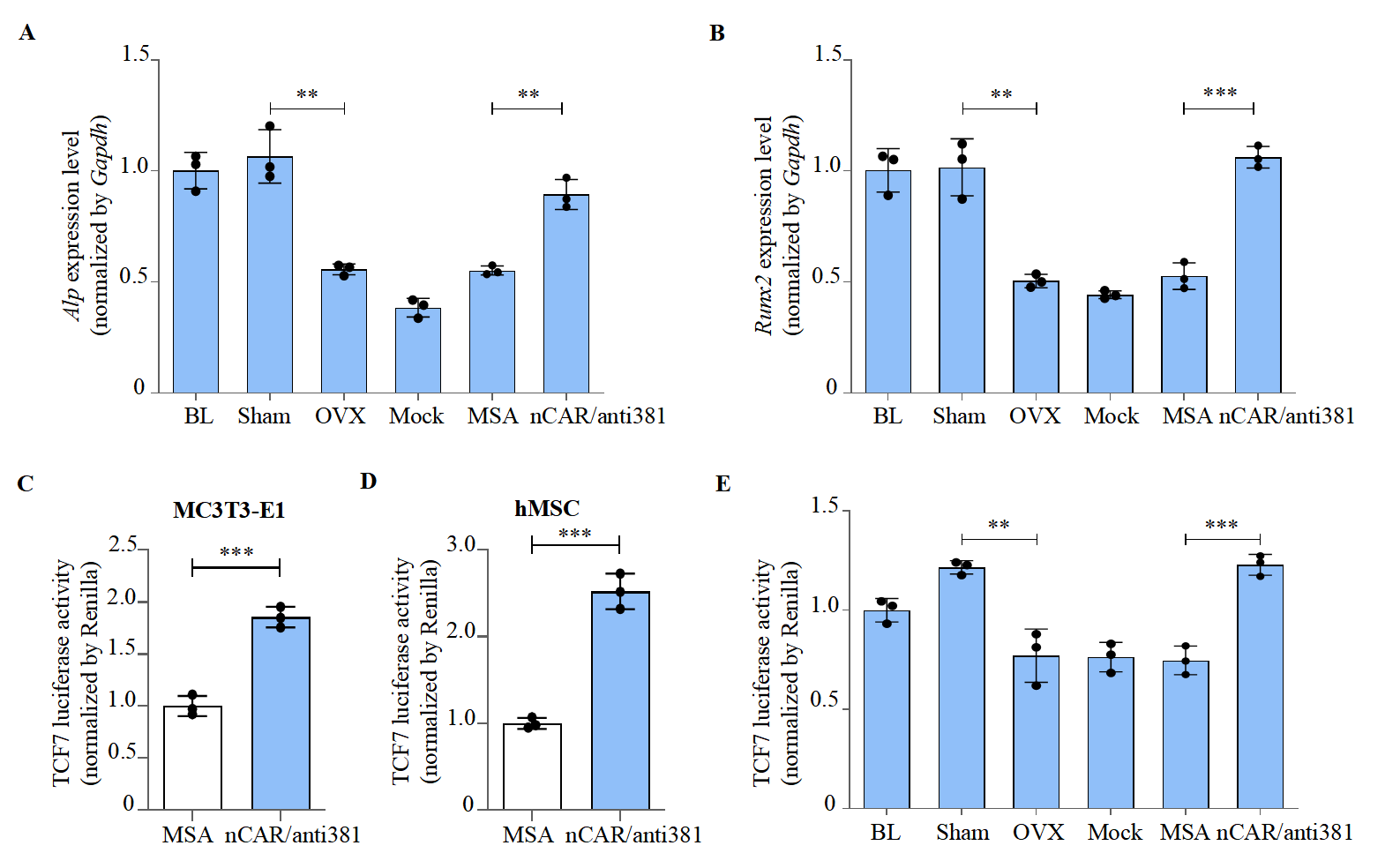

Supplement: Supplementary file 17 — Supplementary Material 17 [file 41598_2025_5826_MOESM17_ESM.tif]

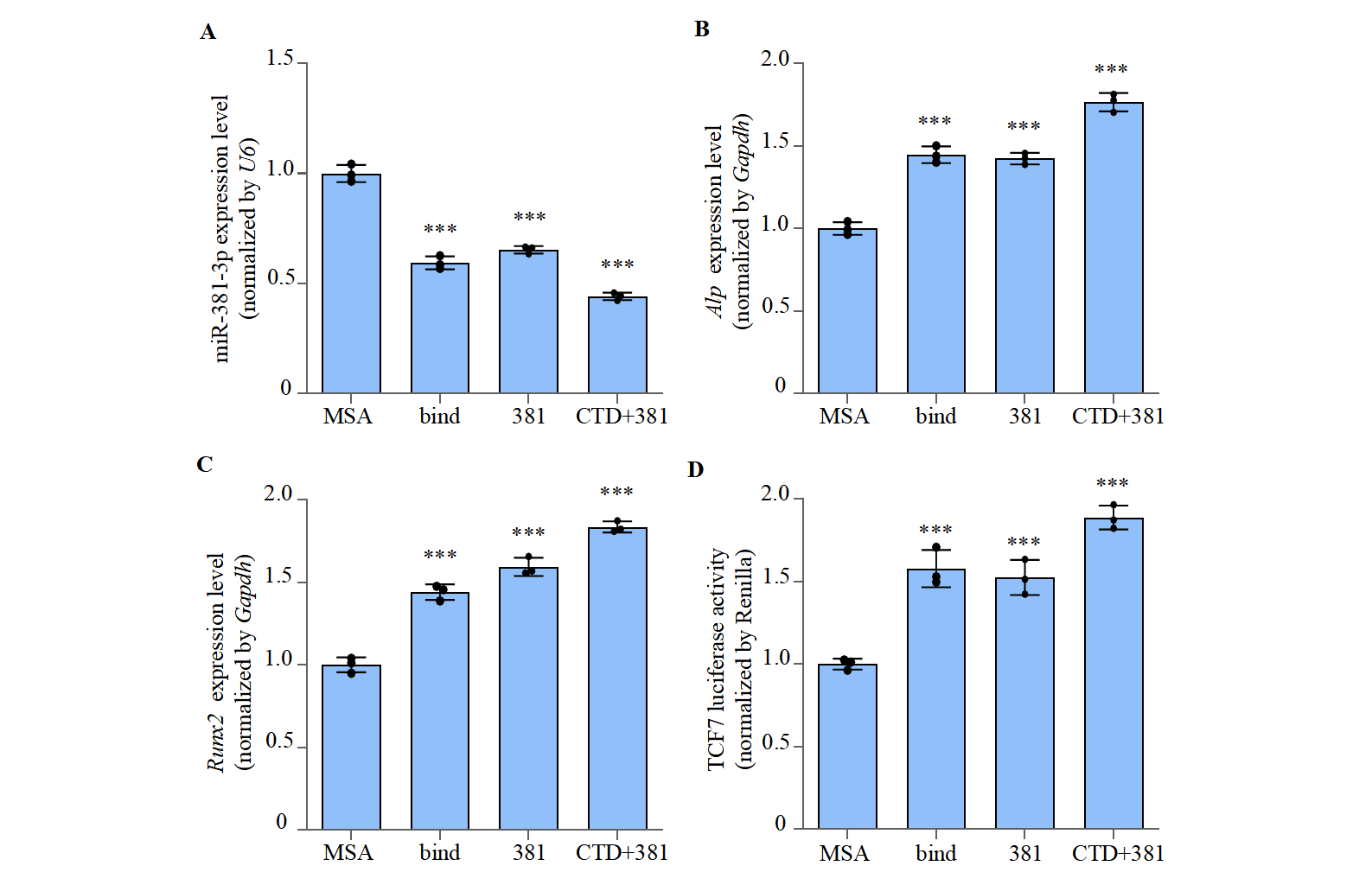

Supplement: Supplementary file 18 — Supplementary Material 18 [file 41598_2025_5826_MOESM18_ESM.tif]

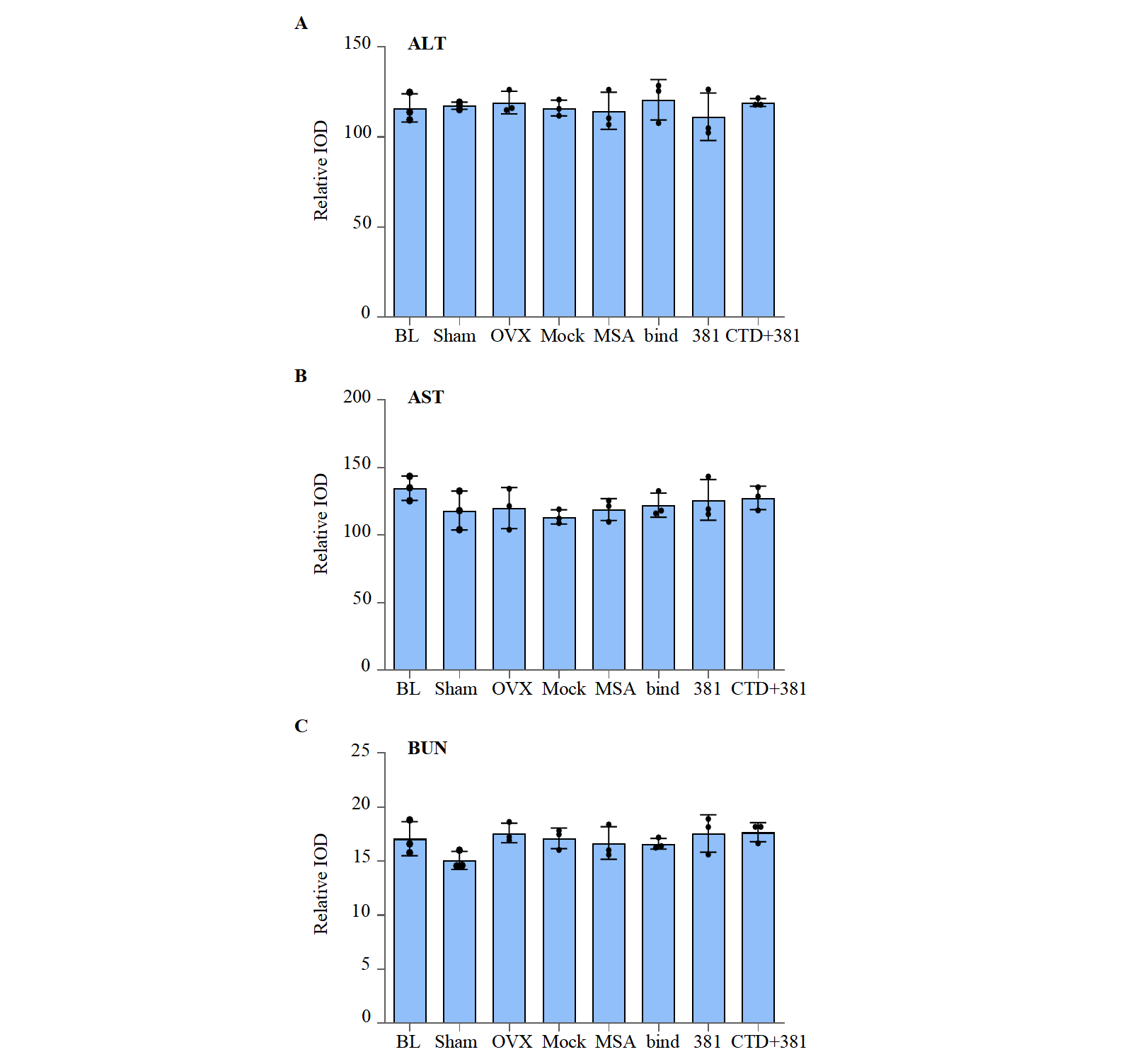

Supplement: Supplementary file 19 — Supplementary Material 19 [file 41598_2025_5826_MOESM19_ESM.tif]

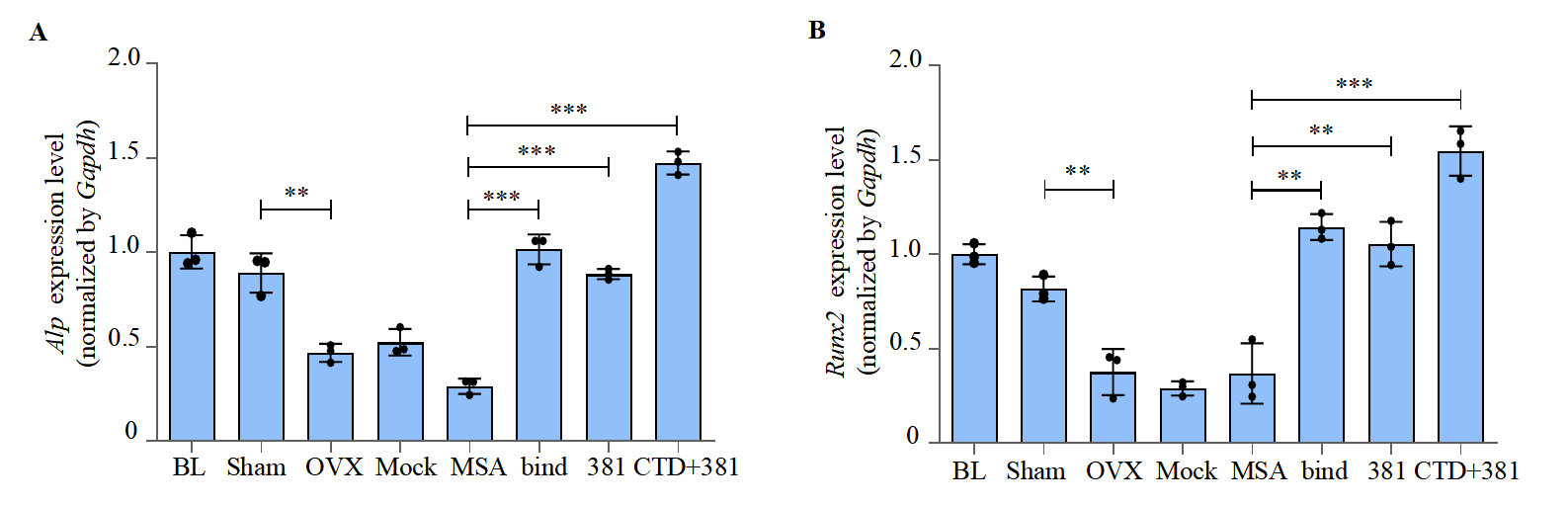

Supplement: Supplementary file 20 — Supplementary Material 20 [file 41598_2025_5826_MOESM20_ESM.tif]

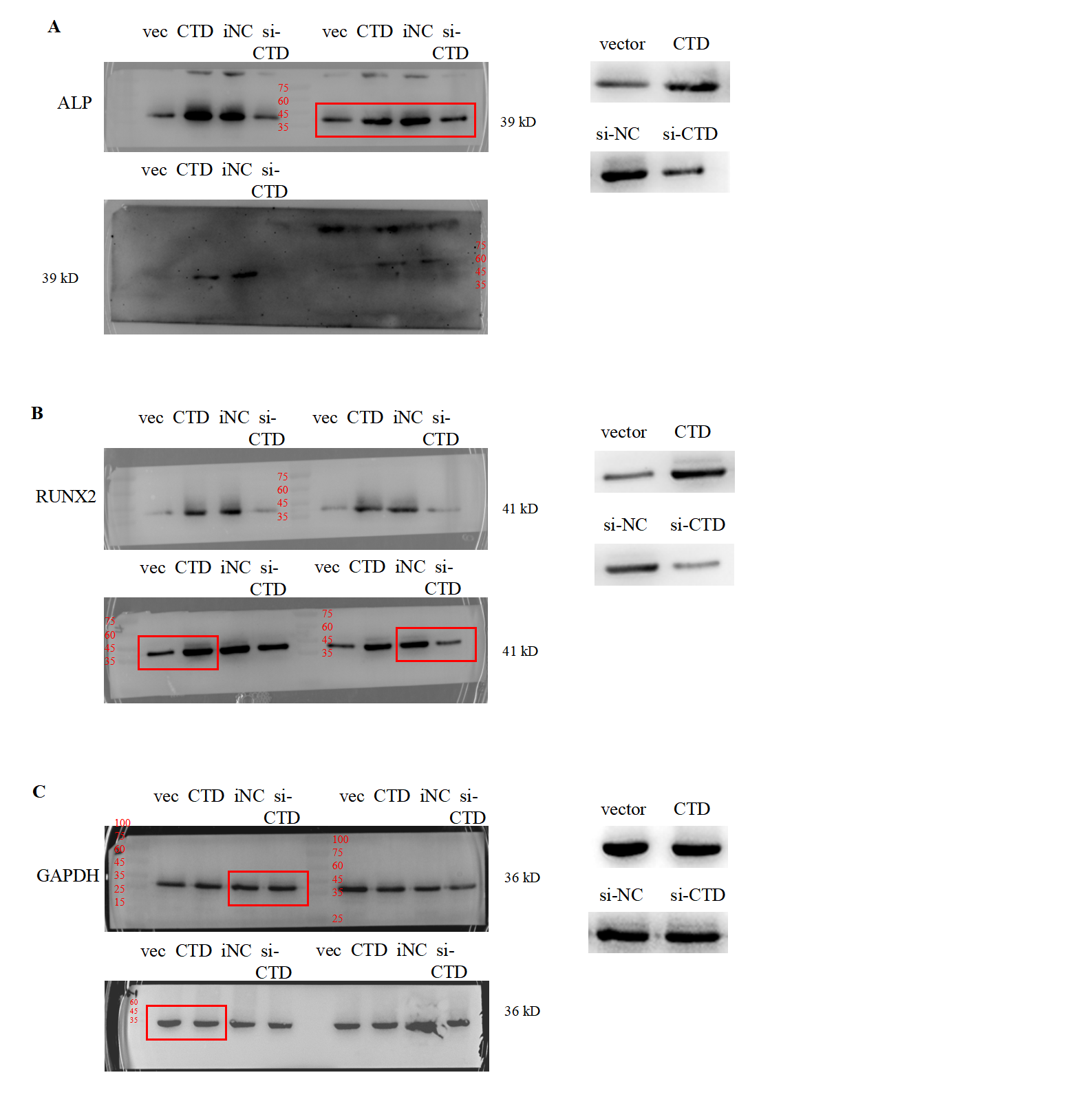

Supplement: Supplementary file 21 — Supplementary Material 21 [file 41598_2025_5826_MOESM21_ESM.tif]

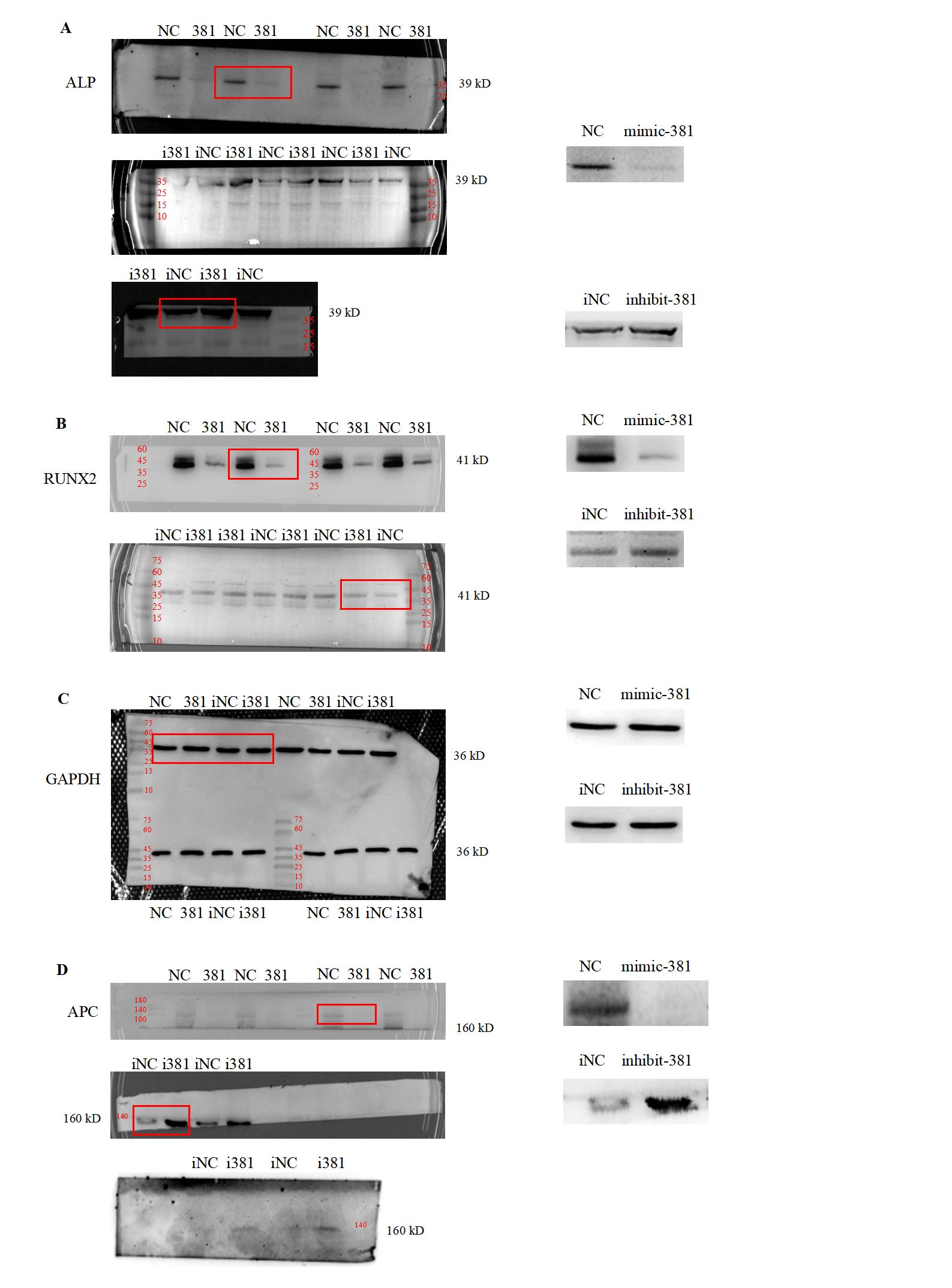

Supplement: Supplementary file 22 — Supplementary Material 22 [file 41598_2025_5826_MOESM22_ESM.tif]

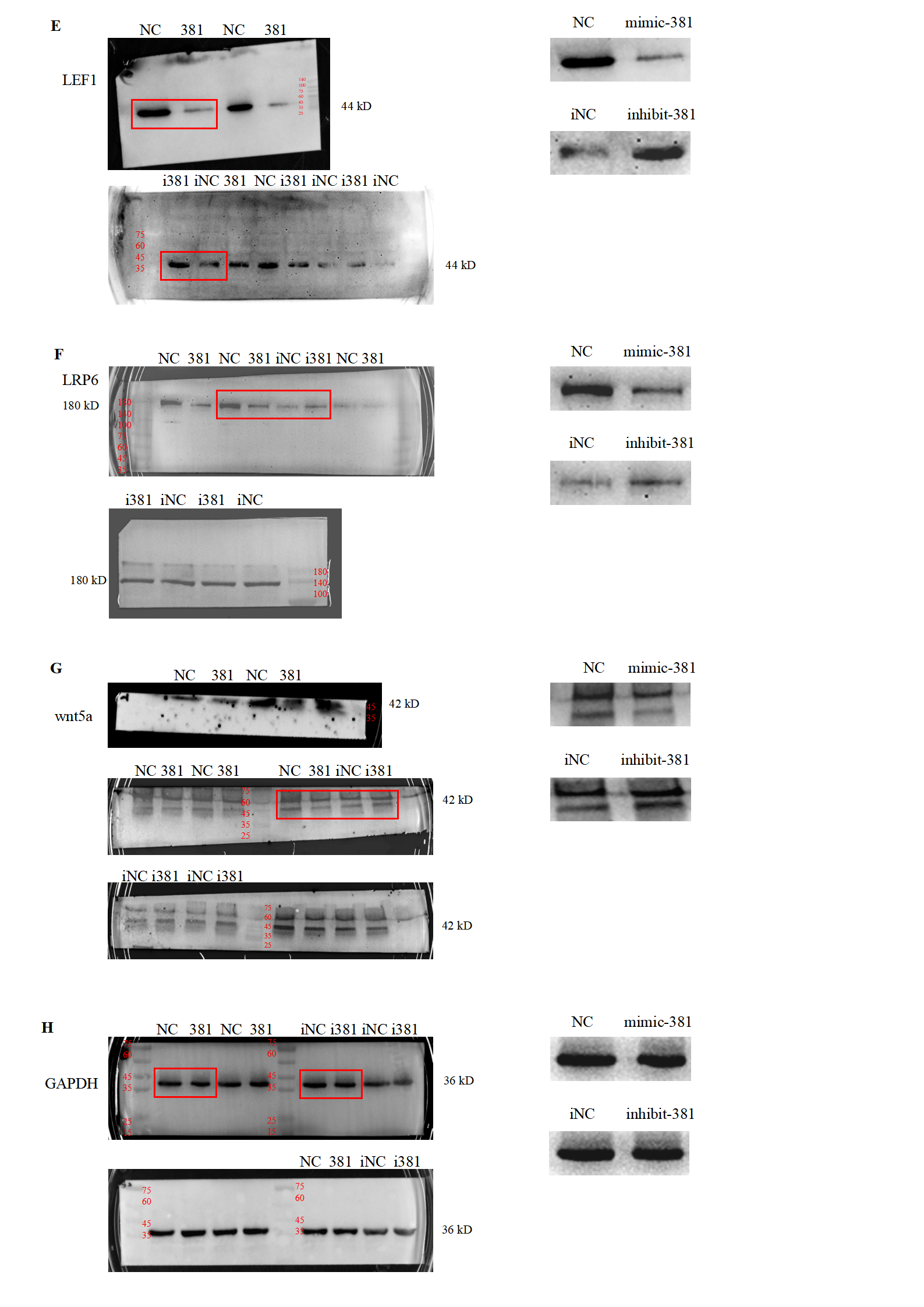

Supplement: Supplementary file 23 — Supplementary Material 23 [file 41598_2025_5826_MOESM23_ESM.tif]

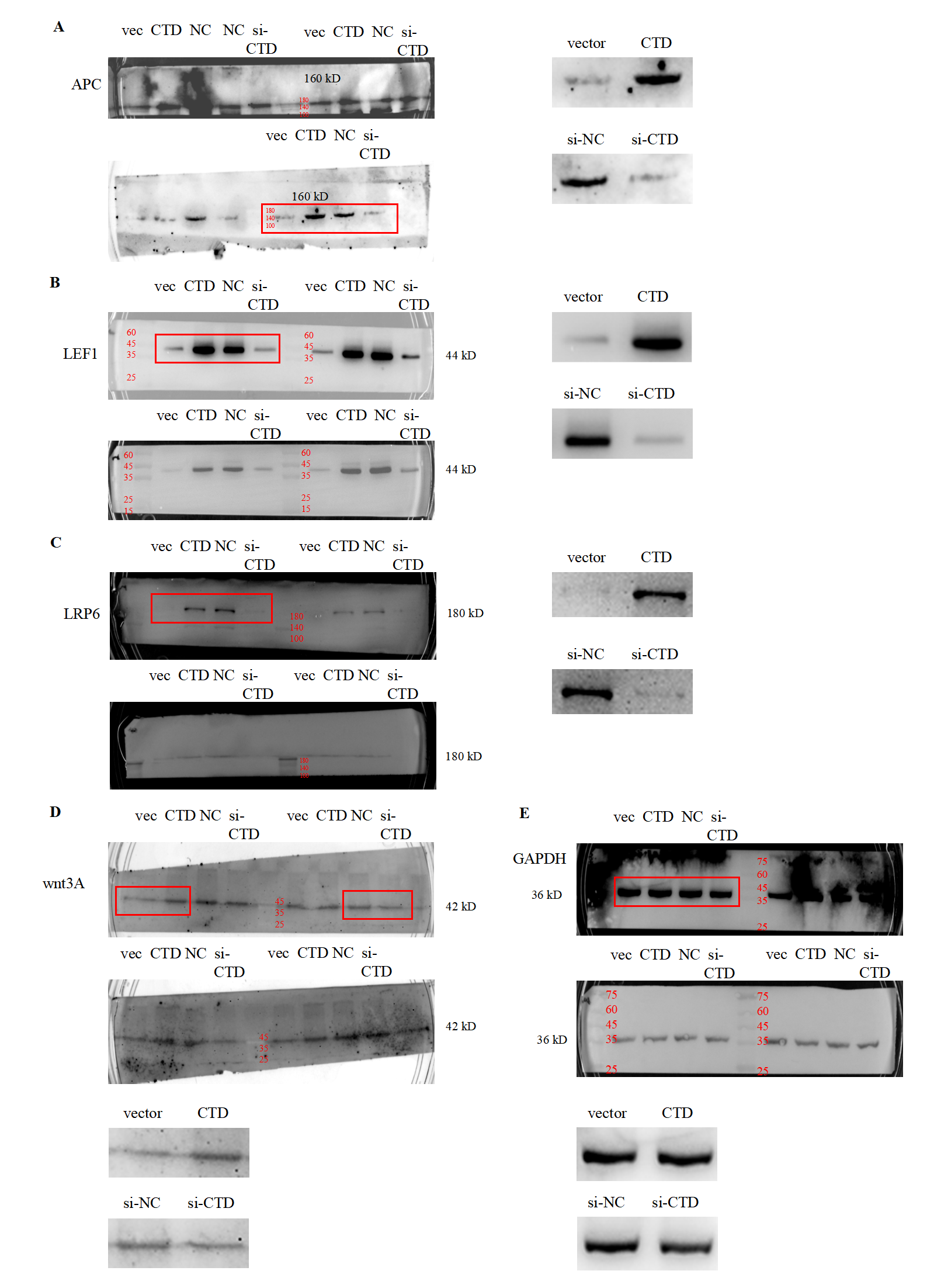

Supplement: Supplementary file 24 — Supplementary Material 24 [file 41598_2025_5826_MOESM24_ESM.tif]
